# Externally validated and clinically useful machine learning algorithms to support patient-related decision-making in oncology: a scoping review

**DOI:** 10.1186/s12874-025-02463-y

**Published:** 2025-02-21

**Authors:** Catarina Sousa Santos, Mário Amorim-Lopes

**Affiliations:** Institute for Systems and Computer Engineering, Technology and Science (INESC TEC), Porto, Portugal

**Keywords:** Cancer, Clinical decision support, Clinical utility, Clinical validity, Decision-making, Deep learning, Digital twins, External validation, Machine learning, Prediction models

## Abstract

**Background:**

This scoping review systematically maps externally validated machine learning (ML)-based models in cancer patient care, quantifying their performance, and clinical utility, and examining relationships between models, cancer types, and clinical decisions. By synthesizing evidence, this study identifies, strengths, limitations, and areas requiring further research.

**Methods:**

The review followed the Joanna Briggs Institute's methodology, Preferred Reporting Items for Systematic Reviews and Meta-Analyses extension for Scoping Reviews guidelines, and the Population, Concept, and Context mnemonic. Searches were conducted across Embase, IEEE Xplore, PubMed, Scopus, and Web of Science (January 2014-September 2022), targeting English-language quantitative studies in Q1 journals (SciMago Journal and Country Ranking > 1) that used ML to evaluate clinical outcomes for human cancer patients with commonly available data. Eligible models required external validation, clinical utility assessment, and performance metric reporting. Studies involving genetics, synthetic patients, plants, or animals were excluded. Results were presented in tabular, graphical, and descriptive form.

**Results:**

From 4023 deduplicated abstracts and 636 full-text reviews, 56 studies (2018–2022) met the inclusion criteria, covering diverse cancer types and applications. Convolutional neural networks were most prevalent, demonstrating high performance, followed by gradient- and decision tree-based algorithms. Other algorithms, though underrepresented, showed promise. Lung and digestive system cancers were most frequently studied, focusing on diagnosis and outcome predictions. Most studies were retrospective and multi-institutional, primarily using image-based data, followed by text-based and hybrid approaches. Clinical utility assessments involved 499 clinicians and 12 tools, indicating improved clinician performance with AI assistance and superior performance to standard clinical systems.

**Discussion:**

Interest in ML-based clinical decision-making has grown in recent years alongside increased multi-institutional collaboration. However, small sample sizes likely impacted data quality and generalizability. Persistent challenges include limited international validation across ethnicities, inconsistent data sharing, disparities in validation metrics, and insufficient calibration reporting, hindering model comparison reliability.

**Conclusion:**

Successful integration of ML in oncology decision-making requires standardized data and methodologies, larger sample sizes, greater transparency, and robust validation and clinical utility assessments.

**Other:**

Financed by FCT—Fundação para a Ciência e a Tecnologia (Portugal, project LA/P/0063/2020, grant 2021.09040.BD) as part of CSS’s Ph.D. This work was not registered.

**Graphical Abstract:**

A visual summary (graphical abstract) encapsulating the core findings and future directions of ML applications in oncology patient care. 
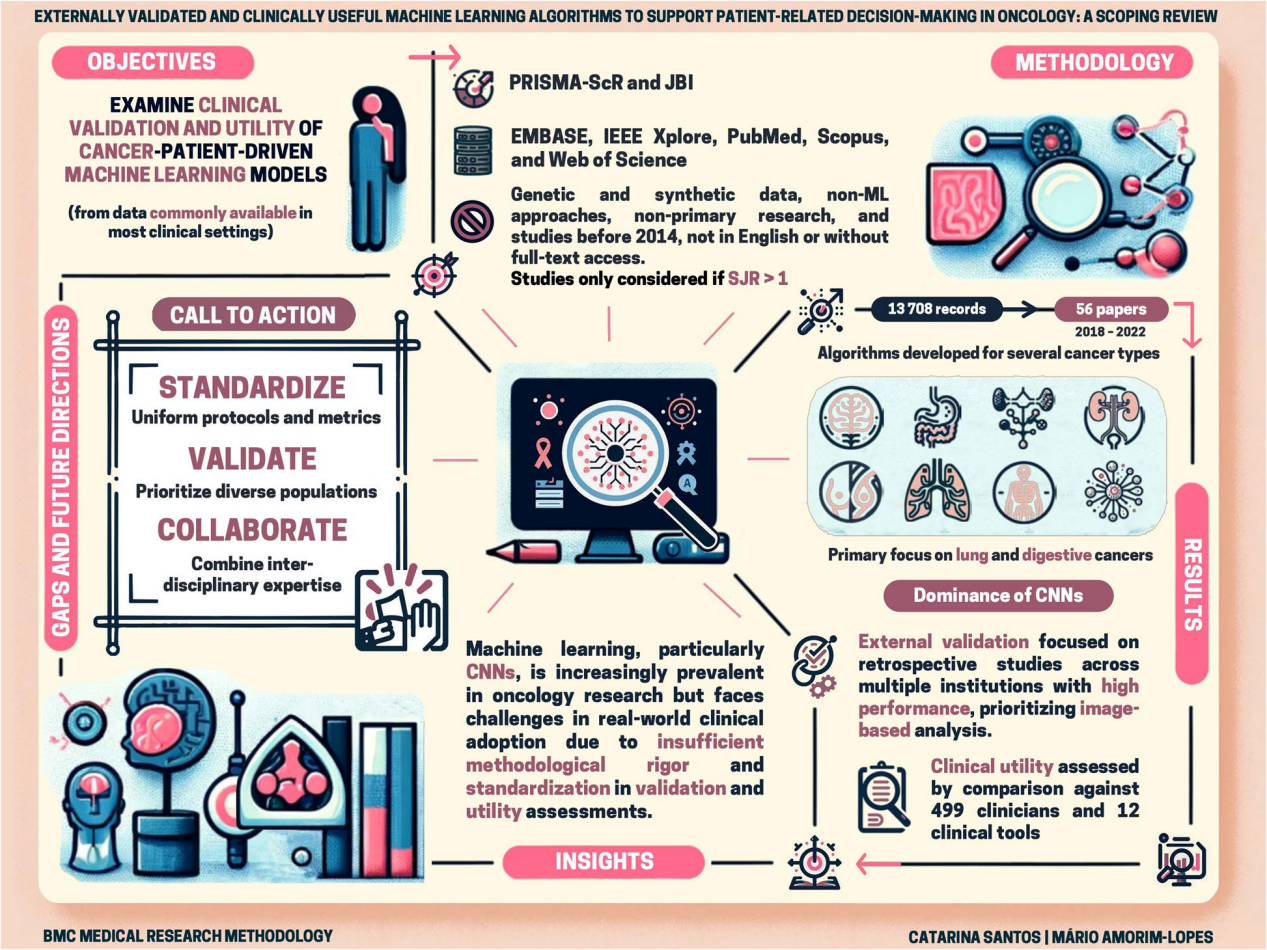

**Supplementary Information:**

The online version contains supplementary material available at 10.1186/s12874-025-02463-y.

## Introduction

Finding a cure for cancer, in its many forms, is still a tremendously complex problem. Despite continuous advances in understanding its biological foundations and the emergence of new treatment possibilities, this disease is still the world's second-leading cause of death [1, 2], causing an enormous socioeconomic burden [3] and an immense workload for physicians [4, 5].

As part of the procedures to diagnose and treat patients with cancer, practitioners collect massive amounts of data, including clinical notes, previous conditions, diagnoses, treatments, prescriptions, laboratory test results, radiological images, and phenotypic and genotypic features. Along with any prior patient-specific knowledge in the same or other healthcare contexts, this information is increasingly stored in virtual collections – electronic health records (EHRs) [6–8]. Notwithstanding the potential of this digitization, the resulting exponential, ever-increasing data expansion – both in volume and complexity – has inevitably shortened the time for clinicians to learn, follow emerging clinical guidelines, and gather all relevant information for proper care [4, 5, 9]. Indeed, with a single patient estimated to generate up to 8 Gb of raw input [6] ranging from unstructured clinical narratives [7, 10] to scanned documents [8], automated techniques have undeniably become required to distill insight from EHRs and assist in decision-making.

In that vein, machine learning (ML) – a branch of Artificial Intelligence (AI) with the ability to learn from and identify patterns in the available data [11–13] – is increasingly used in healthcare to model patient-specific predictive, prognostic, or prescriptive assessments at the point of decision-making [7, 12, 14, 15]. In this context, ML models can be deployed as standalone applications or fall into several technologies, such as clinical decision support (CDS) and computer-aided detection (CADe) or diagnosis (CADx) systems [11, 16]. The main difference between these tools concerns the type of data used for model development: while CADx and CADe approaches rely on imaging, CDS systems usually involve text-based information, such as test results, comorbidities, patient history, and other relevant clinical variables [17].

Machine learning can be divided into two subtypes, supervised and unsupervised learning, separated by the use of labeled or unlabeled datasets [6, 7]. On the one hand, supervised learning models – e.g., support vector machines (SVMs), gradient boosting (GB), random forests (RF), and logistic regression [18] – correlate previously organized features (such as unique patient characteristics) with known outcomes [13, 14, 18, 19]. This approach deals with two types of problems: (i) classification, to produce discrete outputs (or classes), for example, to predict tumor malignancy [14, 20]; and (ii) regression, to estimate continuous values [13, 20]. In healthcare, regression algorithms can be used, for instance, to determine the risk of developing lesions or sequelae over time [14, 15]; or to establish an adequate dose of medication to administer to a specific patient [7, 20]. On the other hand, unsupervised learning methods focus on finding natural patterns in unlabeled data [6, 11, 13, 14]. These models – including principal component analysis (PCA), k-means, gaussian mixture models, density-based spatial clustering of applications with noise (DBSCAN), and balanced iterative reducing and clustering (BIRCH) – are used to find relationships between variables, assign them to different groups according to their similarities (clustering), and to prioritize and reduce the number of features in the dataset (dimensionality reduction) [10, 14, 20].

A specific set of methods, artificial neural networks (ANNs), has even been the basis for a subcategory of machine learning termed deep learning (DL) [6, 13, 21]. Designed to (partially) emulate human neuronal processing, ANNs are composed of artificial neurons (or nodes), interconnected and stacked into three types of layers [6, 22]: (i) *input layer*, containing the original dataset variables; (ii) *hidden layer(s)*, where the data is processed at a certain level of representation [21]; and (iii) *output layer*, with the attained results. In contrast to standard ANNs, usually limited to one hidden layer and still requiring labeled features [22, 23], deep neural networks (DNNs) can derive knowledge from two or more increasing levels of abstraction, with their depth growing along with the number of hidden layers [6, 21]. DNNs (e.g., convolutional and recurrent neural networks) can accurately detect and classify patterns in complex labeled or unlabeled datasets [21, 24, 25], having produced ground-breaking results in numerous areas, including image, pattern, and language recognition [13, 21, 25].

Over the years, several ML- and DL-based tools have been developed to support clinical decision-making in oncology, with many reported benefits. First, these methods can accurately predict cancer susceptibility, recurrence, survival, and risk of complications according to multiple constraints and therapeutic paths [4, 19]. Second, these can be employed in gene expression analysis to predict mutations, proving useful in targeted gene therapy [24, 26]. Third, artificially intelligent approaches in imaging analysis are usually used for tumor monitoring, detection (CADe), segmentation, diagnosis (CADx), and staging [26]. In particular, ML can be paired with radiomics, a quantitative imaging approach that deconstructs medical images into mineable features. ML-based radiomic pipelines, most commonly applied in oncology [27], are usually composed of four sequential stages [26–29]: (i) image retrieval and segmentation, to delineate regions or volumes of interest (for two- or three-dimensional images, respectively); (ii) high-dimensional quantitative feature extraction, to unravel tumor pathophysiology into measurable biomarkers, such as size, volume, texture, shape, and intensity; (iii) feature reduction, to explore relationships between variables to remove redundant or correlated features; and (iv) prognostic/predictive, to link specific features with possible outcomes. By mapping the whole tumor and its adjacent tissues [28], this technique allows performing dynamic virtual biopsies, which can be used to capture spatial and temporal intra-tumoral heterogeneity [26, 28, 29], a key factor linked with tumor aggressiveness and poor treatment responses and survival [28, 30]. These results can be integrated with other available sources of clinical, pathological, and genomic data and leveraged for individualized decision-making to, for example, determine chemo- or radiotherapy doses, treatment-resistant regions, or the best sites to perform an actual biopsy [28].

Finally, efforts have recently been shown to develop digital twins (DTs) for cancer patients [31]. In a medical context, DTs can be described as dynamic virtual replicas modeled intelligently after each physical patient's medical, behavioral, and environmental variables, used for real-time simulations [32–34]. Here, DTs provide clinical support by non-invasively anticipating treatment responses, predicting drug effectiveness, monitoring health indicators, and detecting abnormalities, thus easing decision-making and avoiding unnecessary costs and ineffective procedures [33, 35, 36].

Because of these unique capabilities, ML-and DL-based approaches have unleashed the potential to revolutionize standard healthcare, especially when made available to practitioners at the point of care. Nonetheless, the overwhelming majority of algorithms developed for cancer-related decisions have yet to reach oncology practice [4, 37], mainly due to subpar methodological reporting and validation standards [23, 37–39]. Showing performance on the patients used for development (internal validation) is insufficient, particularly for small sample sizes [40]; as predictions are modeled after that specific cohort, results can be misleading (e.g., biased or overfitted) and non-generalizable to new case mixes [38, 41–43].

Thus, before a new or updated artificially intelligent method can be adopted in clinical practice, it must undergo a thorough evaluation process, which usually consists of external (ideally, clinical) validation and the assessment of clinical utility. First, to ensure model reproducibility (or external validity) and increase confidence in its predictions or estimates, its performance should be evaluated in separate, independent, and comprehensive patient datasets representing the intended target setting(s) [23, 38]. Specifically, the following metrics should be reported [29, 38, 40, 42]: (i) calibration, i.e., the ratio between predicted and observed outcomes, ideally revealed graphically in a calibration plot (to depict the whole range of predictions); and (ii) discrimination, that is, the ability to separate individuals with or without the event of interest. For regression models (continuous outcomes), discrimination is usually shown via concordance (C) index or mean absolute or squared error [38, 43]. For classification tasks (discrete/binary outcomes), discrimination metrics can include the area under the ROC curve (AUC), accuracy, sensitivity, specificity, precision (or positive predictive value), or F-score (i.e., Dice similarity) [38, 40, 43]. Second, this information should always be complemented by evaluating clinical utility, i.e., quantifying the impact of the developed tool on decision-making – and, subsequently, on patient outcomes – through comparative analyses [38, 40, 43]. These comparisons include, for example, clinicians performing the same task with or without assistance, patient outcomes before and after implementation, or, although less informative, direct comparison between well-established models developed for the same end [23, 38, 40, 43]. If possible, this evaluation should preferably be carried out in randomized clinical trials (to minimize confounding variables) or, at least, prospective observational studies so that impact may be assessed over time [42]. Lastly, to ensure clinical validity, this process should involve real-world data (RWD), that is, information routinely collected from actual patients (for example, from EHRs and wearable or mobile devices) [42, 43].

In the context of this scoping review, we conducted a preliminary search of the PubMed database regarding externally validated ML models developed for patient-related decision-making in oncology. Firstly, although several decision support-focused publications do continue to emerge, they are either: (i) focused on context-specific applications, such as evaluation for a specific type of tumor or field [44]; (ii) not approached from a machine learning perspective, that is, not stating which algorithms were used, their performance or their clinical validity [45, 46]; or (iii) outdated [19]. Secondly, only two reports concerning external validation in oncology were found, focused on shortcomings, lack of reporting standards, and risk of bias [37, 39]. However, none of these reviews report: (i) which were the externally validated algorithms; (ii) how the validation studies were designed and their target populations; (ii) if performance was compared against expert clinicians or gold standards and using real-world data; and (iii) if any links can be made between specific algorithms and cancer variants. Since processing mechanisms for different cancer types can be contrasting, addressing the last issue is particularly relevant. Stellar examples are melanomas and other tumors requiring imaging analysis, which have proven to be accurately identified with neural networks (see [47], for example). Accordingly, further connections could and should be made between different types of cancer and frameworks with specific ML techniques.

For the reasons abovementioned, we conducted a scoping review to systematically map externally validated and clinically useful ML-based models developed for patient-related decision-making in the broad scope of oncology practice. Namely, we aimed to report on their validation and impact on decision-making (clinical utility), attempt to associate specific models with particular types of cancer and decisions to make by quantifying their performance, and unveil research gaps in this field. We hope that our findings can be translated into efficient implementations. The ultimate goal is to simplify the decision process and reduce misprescriptions, thus lowering clinicians' workload, increasing confidence, and avoiding misuse and malapplied AI, potentially leading to better healthcare.

The remainder of this paper is organized as follows. Section "Methods" details the methodological approach for the scoping review, including the databases and search terms used and the inclusion and exclusion criteria for data analyses. Section "Results" provides a critical qualitative and quantitative synthesis of external validation and clinical utility assessment. Finally, Section "Discussion" concerns the discussion, where feasible associations between particular types of cancer and ML algorithms are established, the limitations currently faced by ML are outlined, research gaps are presented, conclusions are drawn, and guidance is provided for further work. 

## Methods

This scoping review was conducted according to the updated Joanna Briggs Institute (JBI) methodology for scoping reviews [48, 49], which we also used to develop our protocol (see Additional file 1). In addition, it followed the Preferred Reporting Items for Systematic Reviews and Meta-Analysis extension for Scoping Reviews (PRISMA-ScR) [50] checklist, adapted to encompass the PRISMA 2020 statement [51] (the filled PRISMA2020 Checklist is provided in Additional file 2). Furthermore, as recommended by JBI [48, 49], the Population/Concept/Context (PCC) mnemonic guided the identification of the main concepts, research questions, and search strategy in this review. Here, the population consists of cancer patients (with no restrictions). The concept is externally validated and clinically useful machine or deep learning algorithms to assist decision-making regarding clinical outcomes for cancer patients. The context is oncology care in any setting. Our methodology (and PCC elements) is described in detail in our protocol and summarily presented below.

### Research questions

The research questions and sub-questions were outlined as follows:

- What externally validated machine learning algorithms have been developed to assist patient-related decision-making in oncology practice?
  - ◦ For what types of cancer variants and clinical outcomes were these models developed?
  - ◦ How were the validation studies designed?
  - ◦ Which populations and types of inputs were used?
  - ◦ Have these methods been tested on real-world data?
  - ◦ Have the models been implemented in clinical practice?
  - ◦ How was performance assessed during external validation?
- How was clinical utility established for these methods?
  - ◦ Which comparators and metrics have been used?
- Which machine learning algorithms show the best performance depending on the type of cancer, clinical modalities, and the decision(s) to be made?
  - ◦ What are the reported effects of these ML-based models on decision-making and outcomes?
- What are the research gaps in this field?

### Types of sources and search strategy

This scoping review considered quantitative experimental, quasi-experimental, and observational study designs, including randomized and non-randomized controlled trials, before and after studies, prospective and retrospective cohort studies, and any additional relevant quantitative and comparative research frameworks. Conference abstracts, qualitative studies, and secondary research designs (such as reviews, editorials, letters, and book chapters) were not considered due to not typically reporting individual (if any) performance metrics, thus impeding quantitative analyses. Grey literature was also not included. To limit the scope of this review and increase reproducibility, it only encompassed peer-reviewed journal articles with institutional or open full-text access. Furthermore, to ensure quality and reliable reporting, papers were only assessed for eligibility if published in journals whose Scimago Journal and Country Rank (SJR, 2021), an indicator of scientific journal prestige, is higher than one and whose best quartile is Q1 [52].

The search strategy aimed to locate primary research papers published in peer-reviewed journals. As suggested by JBI, a 3-step search strategy was executed. First, the first author undertook a limited search of PubMed to identify articles on the topic. As a result of this search, keywords were divided into three categories: machine-learning-based decision-making (*"*machine learning*"* OR *"*deep learning*"* OR *"*classification*"* OR *"*regression*"* OR *"*clinical decision support*"* OR *"*computer-aided diagnosis*"* OR *"*computer-aided detection*"* OR *"*digital twin(s)*"* OR *"*decision-making*"*), cancer (*"*cancer*"* OR *"*oncology*"* OR *"*tumor(s)*"* OR *"*neoplasm(s)*" OR "*malignancy*"*), and evaluation (*"*comparison*"* OR *"*performance*"* OR *"*valid*"). This search strategy and the inclusion criteria were deliberately designed without imposing limitations on ML, patient profiles, or specific cancer-related settings, ensuring the inclusion of a wide range of relevant papers and maximizing the comprehensiveness of the review. Second, these keywords were used to develop a complete search strategy for the EMBASE, IEEE Xplore, PubMed, Scopus, and Web of Science databases. The search terms were adapted to each database (see Additional file 3). This study selected IEEE Xplore to address computing articles, PubMed and EMBASE to include biomedical literature, and Scopus and Web of Science to cover multidisciplinary reports. Only publications written in English were considered for inclusion. Studies published from January 1, 2014 were searched, as this year aligns with when deep learning became mainstream [23]. Third, the reference list of all included sources of evidence was screened for additional studies.

### Eligibility criteria

This review included new or updated externally validated machine or deep learning algorithms to assist decision-making regarding clinical outcomes for cancer patients, with no restrictions regarding cancer types or specific demographics. Samples could consist of human patients or lesions (for image analysis), provided that the focus was on cancer patient outcomes and data routinely available in clinical settings were used. All commonly known machine learning algorithms and digital twin approaches were considered, as these align with clinical prediction models. Although not universally qualified as an external assessment [38], papers reporting model performance on temporally different datasets (temporal validation) were also included. The assessment of clinical utility was mandatory, but all clinical comparators were included (e.g., comparison against standard care, before-after studies, and clinician performance with and without the tool, among many others).

Studies were discarded if they:

- Were not primary research articles published in peer-reviewed journals whose SJR was equal to or higher than one. This criterion was established to ensure the inclusion of research from sources recognized for their quality and impact, thereby enhancing the reliability and relevance of the synthesized evidence.
- Used synthetic patients or animals. This restriction was imposed to prioritize real-world applicability in clinical settings, where outcomes and decisions are based on authentic human patient data. Although an instrumental resource, synthetic data may not fully encapsulate the complexity and variability inherent in clinical practice [53].
- Concerned sequencing, omics, and molecular biomarker discovery. These studies were excluded due to the specialized and currently less accessible nature of omic information in routine clinical settings, a challenge particularly pronounced for proteomics and metabolomics [54]. This review centers on algorithms ready for immediate use in clinical decision-making, aligning with the immediate needs of healthcare practices.
- Used non-machine learning approaches (for traditional statistical algorithms such as logistic regression and naïve Bayes, these were excluded unless explicitly described as machine learning models);
- Developed algorithms for anything other than patient care (such as medical education, structured data collection, text classification, cohort-specific assessments, or EHR dashboards);
- Were not primarily focused on oncology;
- Did not present performance metrics for external validation (either in the current or previous papers). These metrics are required to verify the algorithms' reliability and generalizability beyond the development environment, a key indicator of their readiness for clinical application.
- Had not assessed clinical utility. This assessment is critical for demonstrating an algorithm's palpable benefit in improving patient care, an essential aspect of its value to the medical community.
- Were not written in English. This requirement ensures wide accessibility and comprehension of the review's findings within the global scientific community.
- Did not have full-text access (inaccessible or inexistent), as this limitation prevents an in-depth analysis of the studies’ methodologies and outcomes.

### Study selection

**Fig. 1:**
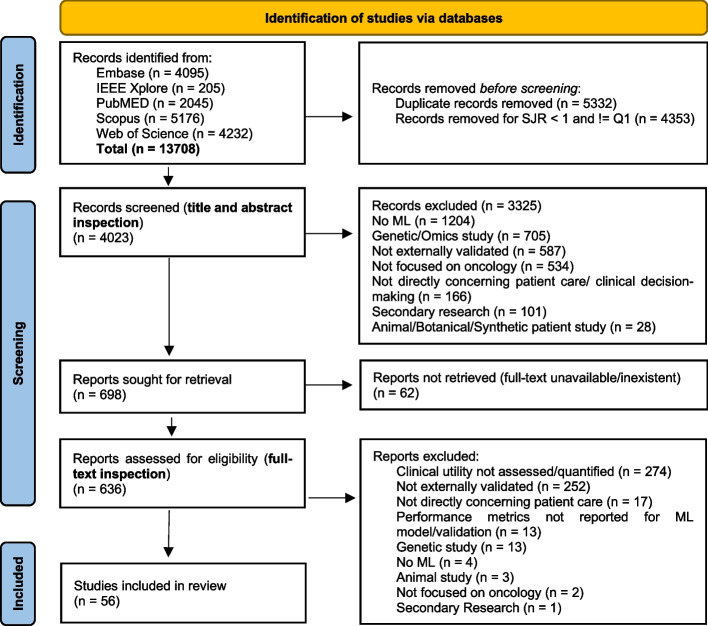
PRISMA flow diagram

Following the search, all identified citations were collated in RIS format, uploaded into EndNote 20.4.1 /2022 (Clarivate Analytics, PA, USA), and deduplicated (first electronically, followed by a manual sweep). A Python script was then used to filter publications by SJR ranking (available at Additional file 4). The remaining citations were imported into a spreadsheet, and titles and abstracts were screened for assessment against the inclusion criteria for the review. Next, a full-text inspection of the potentially relevant sources was carried out. Disagreements at each stage of the selection process were resolved through discussion among the authors. The search and study inclusion process results are presented in a Preferred Reporting Items for Systematic Reviews and Meta-analyses extension for scoping review (PRISMA-ScR) flow diagram [50] updated per the PRISMA 2020 statement [51] (see Fig. 1 in *Results*).

### Data charting

Data were extracted using a data extraction form (available in our protocol – see Additional file 1). These data were stored in Excel spreadsheets and included general information and specific details about the participants, concept, context, study methods, and critical findings relevant to the review questions. No modifications were made to the original form. General study characteristics included the first author, title, year of publication, journal, SJR ranking, and whether limitations were reported and any reporting guidelines were followed. The following information was charted from each source: development design (development and validation or validation only), study design (retrospective versus prospective), care type (primary, secondary, tertiary, or quaternary), general and specific cancer type, the study's focus (e.g., survival or diagnosis), best-performing machine learning method(s), task (classification, regression, or both), type of implementation, interface, system classification (e.g., CADx, CDSS), processing time, software, number of institutions in validation, data availability, validation type, data source (i.e., the country from which the data were obtained), population details (age group, number of patients, number of female and male patients, sample type, and sample size), whether independent validation was performed and real-world data were used, which discrimination and calibration metrics were used to evaluate validation performance, and which comparators and metrics were used to assess the models’ clinical utility. The data is presented in tabular and graphical form, accompanied by a narrative summary. All statistical analyses and graphic illustrations were performed using Pandas 1.3.4 and Matplotlib 3.4.3 (Python 3.9.7).

### Critical appraisal and risk of bias

Besides discarding publications whose SJR was lower than one, no other evaluations concerning data quality were carried out, which aligns with the JBI's protocol for scoping reviews [49].

## Results

### Study selection

A total of 13 708 records were identified in our search, which was last updated on September 30, 2022. As shown in Fig. 1, after duplicate removal and filtering by SJR ranking, the titles and abstracts of 4023 citations from Embase, IEEE Xplore, PubMed, Scopus, and Web of Science were assessed. In this stage, 3325 papers were excluded for not being machine learning-based (*n* = 1204, 29.9%), using genetic variables or omics (*n* = 705, 17.5%), not being externally validated (clearly mentioning performance evaluation by cross-validation or hold-out sampling, *n* = 587, 14.6%), not being focused on oncology (*n* = 534, 13.3%), not regarding patient care or clinical decision-making (e.g., creation of data infrastructures or organizing EHRs, *n* = 166, 4.1%), not being primary research articles (*n* = 101, 2.5%), and not including human patients (*n* = 28, 0.7%). This left 698 papers eligible for full-text inspection, of which 62 were excluded for unavailability. From the remaining 636 reports, 274 (43.1%) were discarded for not assessing or quantifying clinical utility, 252 (39.6%) for not being externally validated, 17 (2.7%) for not directly concerning patient care, 13 (2%) for not reporting performance metrics, 13 (2%) for focusing on gene expression or omics, 4 (0.6%) for not containing machine learning models, 2 (0.3%) for not focusing on oncology and 1 (0.2%) secondary research paper. For example, although seemingly relevant, that is, describing external validation and comparison of diagnostic competence against pathologists, other than reporting intraclass correlation coefficients, Yang et al.'s study [55] did not quantify clinicians' performance, which led to its exclusion. No additional relevant documents were found by screening the included studies. Finally, 56 articles were included in this scoping review. The completed form for the included studies can be found in Additional file 5.

### Study overview

Table 1 summarizes key findings from the 56 studies on patient-centered ML applications in oncology, providing an overview of algorithms, clinical applications, data types, and evaluation methods for clinical utility. The following subsections offer insights into different aspects of the data.

**Table 1:** Summary of machine learning applications in the 56 studies

Organ System	Cancer Type	Number of Studies	ML Algorithm	Clinical Application	Data Type	Clinical Utility Assessment	Key Findings	References
**Central Nervous System**	Brain	3	CNNs, RF, AdaBoost	Detection and Diagnosis	MRI images	Comparative analysis against radiologists	High performance, surpassing the radiologists’ diagnostic accuracy	[56–58]
**Digestive System**	Colorectal	7	CNNs, SVM, LightGBM	Diagnosis, Survival, Outcome Prediction	Clinical data,MRI images, and endoscopy images and videos	Evaluation against radiologists, endoscopists, and two established clinical guidelines (TNM-7 and RECIST)	Effective diagnostics and predictions, performing similarly to clinicians and exceeding routinely used guidelines	[59–65]
	Gastric	5	CNNs, RNN, MLP, SVC	Survival, Recurrence, Diagnosis, Detection, Outcome Prediction	WSIs, CTs, Endoscopic images	Assessment against pathologists, physicians, endoscopists, and RECIST	High accuracy in all tested tasks. Outperformed RECIST and improved clinician performance while aided by the ML tools	[66–70]
	Esophagus	3	CNNs, MLP	Detection, Location, Survival	Clinical data, endoscopic images	Comparison with endoscopists, pathologists, and TNM-8	Overperforms TNM-8. Accurate detection and location, surpassing endoscopists and pathologists	[71–73]
	Liver	2	XGBoost + RF + GBDT, CNNs	Diagnosis, Survival	WSIs, clinical data	Validation against existing prognostic models (TNM-8 and LCSGJ) and pathologists	Assistance by models increases clinicians’ diagnostic performance. Survival predictions more accurate than TNM-8 and LCSGJ	[74, 75]
**Endocrine System**	Pancreas	2	ANN + logistic regression + RF + GB + SVM + CNNs, Random Survival Forest	Survival	Clinical data, CTs	Benchmarking against TNM-8 and mGPS	Outperforms both TNM-8 and mGPS, although with lower sensitivity than TNM-8	[76, 77]
	Thymus	1	SVM	Diagnosis	CT images	Direct comparison with three radiologists	Achieves a higher AUC than the radiologists, but is beaten in sensitivity and specificity	[78]
**Genitourinary System**	Bladder	1	CNN	Outcome prediction	CT images	Direct comparison with two radiologists	Predicts muscular invasiveness with higher accuracy than the radiologists, but with lower sensitivity and specificity	[79]
	Cervix	1	CNN + RNN	Diagnosis	WSIs	Direct comparison with three cytopathologists	Accurate in diagnosing cervical cancer, with higher performance than the cytopathologists	[80]
	Prostate	2	CNN, QSVM	Diagnosis, Risk Stratification	mpMRI scans	Direct comparison with an expert radiologist or PI-RADS v2	Outperforms radiologist. Surpasses PI-RADS v2 in all tested metrics except AUC	[81, 82]
	Uterus	2	LR, CNN	Diagnosis	WSIs, clinical data	Direct comparison against a radiologist and three pathologists	Effective in differentiating malignant from benign lesions. Higher performance than the radiologist and a junior pathologist. Performed similarly to a mid-level and a senior pathologist	[26, 83]
**Integumentary System**	Breast	3	CNNs, XGBoost, RF	Diagnosis, Outcome Prediction, Survival	Mammograms, Ultrasound images	Comparison against three commonly used clinical tools (FRAX, OSTA, and TNM-8) and radiologists assisted by the ML tools	Enhanced screening and outcome predictions, with radiologists’ performance increasing while aided by the ML systems. Increased outcome prediction accuracy compared to the three tools	[84–86]
	Skin	2	CNNs	Detection and Diagnosis	Photographic images	Direct comparison against physicians, dermatologists, and ophtalmologists	High accuracy in skin cancer detection and diagnosis, surpassing all clinicians	[87, 88]
**Respiratory System**	Lung	10	CNNs, XGBoost, kNN + NB + RF, MDA, MLP, N-MTLR	Detection, Diagnosis, Outcome Prediction, Survival, Risk Stratification	CT images, Clinical data	Assessment against clinical tools (Brock, PKU, Mayo, and VA models, PLCOm2012, TNM-8, WHO Performance Status) and radiologists and thoracic surgeons	Improvements in diagnostic accuracy and outcome and survival predictions, performing equally or higher than common prediction tools. Enhanced clinician performance when aided by the tools	[89–98]
	Larynx	1	CNN	Diagnosis	Endoscopic images	Direct comparison against three endoscopists	Improved laryngeal cancer diagnosis for four-way classification, but was outperformed in binary classification	[99]
	Mesothelium	1	CNN + RNN	Diagnosis	WSIs	Direct comparison against three pathologists	Effective in differentiating mesothelial proliferations. Although less specific, exceeds clinicians’ accuracy and specificity	[100]
	Nasopharynx	1	CNN	Diagnosis	Endoscopic images	Comparison against endoscopists and interns	Accurate in differenciating benign from malignant lesions, outperforming all clinicians (slightly less sensitive than interns)	[101]
**Skeletal System**	Bones	4	LogitBoost, ExtraTrees, CNNs, ANN	Diagnosis	PET-CT, X-ray, and MRI images	Direct comparison against radiologists of varying expertise	Accurate differentiation between benign and malignant bone tumors, although not achieving clinician performance	[102–105]
**Several** **(Secondary Cancers from Multiple Primary Sources)**	Organ metastases	3	GBDTs, XGBoost, CNN	Risk, Survival, and Mortality Predictions	Clinical Data, CT images	Comparison against radiologists, oncologists, and TNM-7	Surpassed TNM for survival predictions, but not risk stratification. Outperformed clinicians in almost all tests	[106–108]
	Metastasized Lymph Nodes	2	CNN, XGBoost	Diagnosis	Ultrasound images, CT images	Comparison against radiologists	Accurate in diagnosing metastasized nodes, surpassing clinician performance in direct comparisons and enhancing their performance when aided by AI systems	[109, 110]

*ANN* Artificial Neural Network, *AUC* Area Under the ROC Curve, *CNN* Convolutional neural network, *CT* Computed Tomography, *FRAX* Fracture Risk Assessment Tool, *ExtraTrees* Extremely Randomized Trees, *GBDT* Gradient-Boosted Decision Tree, *kNN* k-Nearest Neighbors, *LCSGJ* Liver Cancer Study Group of Japan, *LightGBM* Light Gradient-Boosting Machine, *LR* Logistic Regression, *MDA* Mixture Discriminant Analysis, *mGPS* Modified Glasgow Prognostic Score, *ML* Machine Learning, *MLP* Multi-layer Perceptron, *mpMRI* Multiparametric MRI, *MRI* Magnetic resonance imaging, *NB* Naïve Bayes, *N-MTLR* Neural Multitask Logistic Regression Model, *OSTA* Osteoporosis Self-Assessment Tool for Asians, *PET-CT* Positron Emission Tomography – Computed Tomography, *PI-RADS* Prostate Imaging Reporting and Data System, *PLCO* Prostate, Lung, Colorectal, and Ovarian Cancer Screening Trial, *QSVM* Quadratic Support Vector Machine, *RF* Random Forests, *RECIST* Response Evaluation Criteria in Solid Tumors, *ROC* Receiver Operating Characteristic, *SVC* Support Vector Classifier, *SVM* Support Vector Machine, *TNM* Tumor, Node, and Metastasis staging system, *TNM-7* Seventh edition of the TNM staging system, *TNM-8* Eighth edition of the TNM staging system, *WSI* Whole-Slide Images, *XGBoost* Extreme Gradient Boosting

#### Journals, years of publication and reporting guidelines

**Fig. 2:**
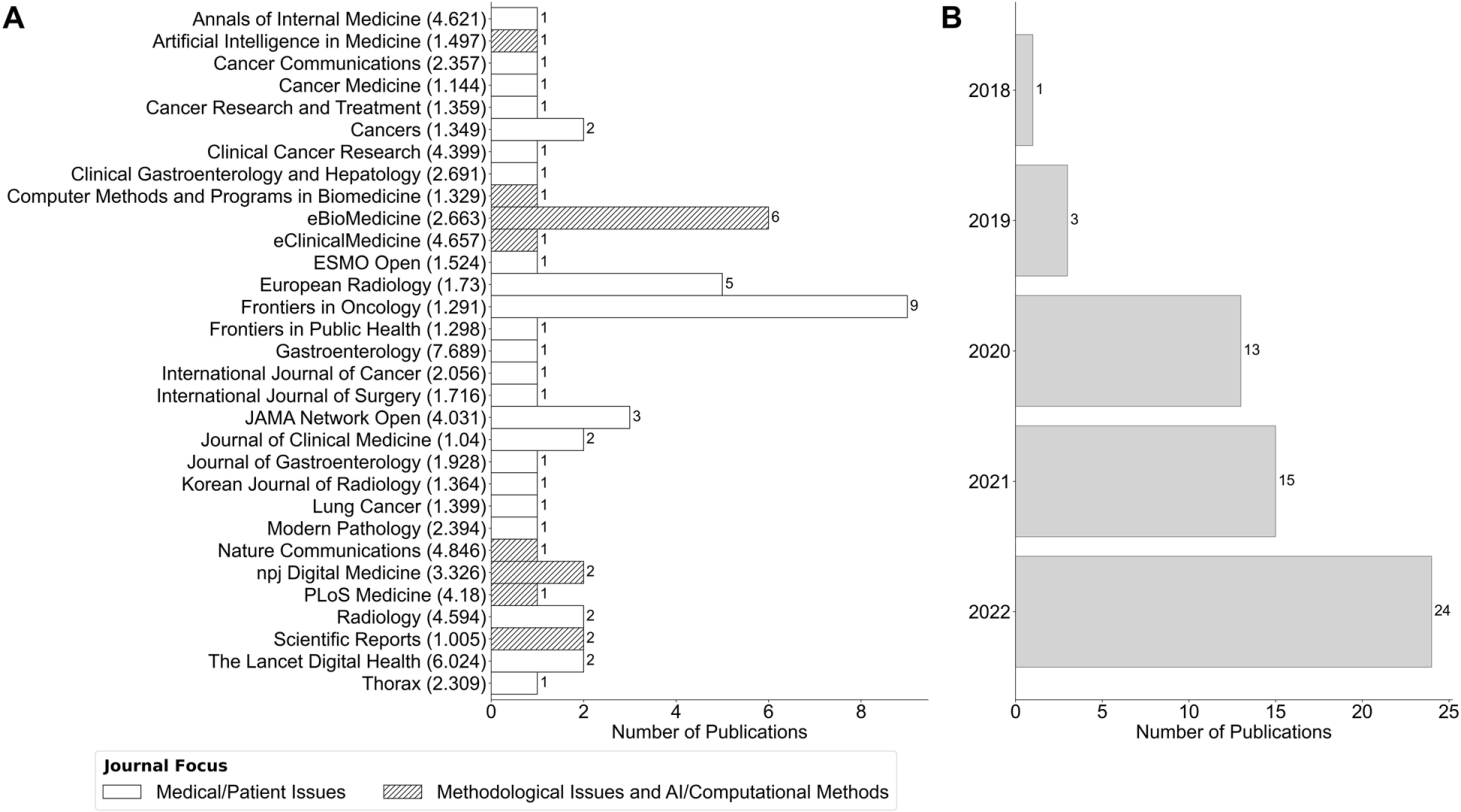
Number of publications per journal (**A**) and year (**B**)

As depicted in Fig. 2A, the included articles were retrieved from 31 journals with an average SJR (2021) of 2.496, from a minimum of 1.005 (*Scientific Reports* [81, 89]) and a maximum of 7.689 (*Gastroenterology* [71]). *Frontiers in Oncology* was the most common source (*n* = 9, 16.07%, SJR = 1.291), followed by *eBioMedicine* (*n* = 6, 10.71%, SJR = 2.9) and *European Radiology* (*n* = 5, 8.93%, SJR = 1.73)*.* Eight (25.8%) of these journals were primarily dedicated to methodological issues and computational methods within artificial intelligence (dashed bars in Fig. 2A), while the remaining twenty-three (74.2%) focused on medical applications and patient-related topics.

Concerning the year of publication, although citations since 2014 were screened, only papers from 2018 and onwards met the inclusion criteria. The number of reports increased substantially after 2020, with 23% (*n* = 13), 27% (*n* = 15), and 43% (*n* = 24) of the sources being from 2020, 2021, and 2022, respectively, versus 2% (*n* = 1) in 2018 [101] and 5% (*n* = 3) in 2019 [81, 90, 99] (Fig. 2B). While the majority did not adhere to any reporting guidelines (*n* = 48, 85.714%), 3 (5.357% [78, 91, 92]) used TRIPOD [111], 3 (5.357% [87, 102, 106]) followed STARD 2015 [112] (commonly used for diagnostic and prognostic studies) [112], and 2 used CONSORT-AI [113] and STROBE [114] (1 each, 1.786%, [88] and [93], respectively). Lastly, caveats were not reported for a small percentage of studies (7.14%, *n* = 4) [72, 80, 84, 107].

#### Algorithms, cancer types and clinical outcomes

The features of the machine learning algorithms found in the included articles are detailed in Table 2. Sixty-two models were described in the 56 documents, with 55.4% (31/56) of the authors explicitly mentioning which algorithms were used in the paper's abstract. Most developers opted for an ensemble approach (*n* = 27, 48.2%), 26 (46.4%) for single models, and three (5.4%) for both [66, 67, 92]. Of the selected studies, 50 (89.3%) were exclusively devoted to classification, 4 to regression (7.1%) [72, 74, 76, 93], and 2 developed both types of models (3.6%) [67, 68]. All models were supervised except in one study (semi-supervised) [73], and 50% of the researchers (*n* = 28) compared their systems against other ML algorithms. Apart from work developed in [106], where the model was silently integrated into the patients' EHRs, all models were deployed as standalone systems. Overall, 30 (53.6%) can be classified as CADx, 19 (33.9%) as CDSS, 2 (3.6%) as CADe [71, 73], and 5 as both CADe and CADx (8.9%) [80, 88, 90]. Regarding interfaces, most tools were desktop-based (*n* = 46, 82.1%), and 10 (17.9%) were deployed as web-based applications [59, 67, 75, 82, 84, 87, 91, 101, 107, 109]. All websites were reported, 43 articles (76.79%) disclosed which software was used, and codes were provided for 11 models (19.6%) [56, 60, 75, 80, 87, 88, 92–94, 103, 108].

**Table 2:** Characteristics, classification and objectives of the machine learning models used in the studies

First Author	General Focus and Models	Objectives and Cancer Types	Interface	System Classification	Software	URL	Explainability Details
Baldwin [96] ^a^	**Diagnosis** (classification): **CNN** (DenseNet)	Estimate the risk of malignancy in **pulmonary** nodules	Desktop-based	CADx	NR	NR	NR
Bi [115] ^b^	**Diagnosis** (classification): **logistic regression** (radiomics-based)	Differentiate stage IA **endometrial cancer** from benign endometrial lesions	Desktop-based	CADx	SuperLearner; Python 3.9.7	NR	NR
Chen [91] ^ab^	**Diagnosis** (classification): XGBoost	Estimate the malignant probability of multiple **pulmonary** nodules	Web-based	CADx	Python	https://mpn.pkuphmodel.com/	SHAP
Cheng [80] ^ab^	Detection, location, and diagnosis (classification): CNNs (ResNet50, pre-trained) + RNN	Automated screening of **cervical cancer** according to cervical slide characteristics from cervical biopsies	Desktop-based	CADe and CADx	C + + , Multi-Threading; TensorRt; Python: 3.6	https://github.com/ShenghuaCheng/Aided-Diagnosis-System-for-Cervical-Cancer-Screening	NR
Choi [89]	**Outcome prediction** (classification): **CNN** (unknown architecture)	Predict visceral pleural invasion in early-stage **lung cancer** patients	Desktop-based	CADx	Keras (V 2.2.4); TensorFlow (V 1.12.0)	NR	Grad-CAM
Choi [62] ^ab^	**Diagnosis** (classification): **CNNs** (Inception-v3 + ResNet-50 + DenseNet-161, pre-trained)	Predict histology of **colorectal** neoplasms in white light colonoscopic images	Desktop-based	CADx	NR	NR	Grad-CAM
de Groof [71] ^ab^	**Detection** (classification): **CNN** (ResNet-Unet, pre-trained)	Primary detection of neoplasia in patients with **Barrett's Esophagus**	Desktop-based	CADe	PyTorch	NR	NR
Deng [97] ^ab^	**Progression-free survival** (classification of survival benefit): **CNN** (EfficientNetV2, pre-trained)	Predict the survival benefits of EGFR-TKIs and ICIs in stage IV non-small-cell **lung** cancer patients using pre-therapy CT images	Desktop-based	CDSS	PyTorch (1.7.1; Python)	NR	NR
Feng [78] ^ab^	**Diagnosis** (classification): **SVM** (radiomics-based)	Simplified risk categorization of **thymic** epithelial tumors	Desktop-based	CADx	Deepwise Research Platform	NR	NR
Gao [58]	**Detection and diagnosis** (classification): **CNN** (2D U-Net (segmentation) + DenseNet2 (classification))	Automated identification and classification of 18 types of **brain** tumors from patient MRI data	Desktop-based	CDSS	Scikit-Learn (V 0.22, Python 3.2)	NR	NR
Gitto [104] ^a^	**Diagnosis (classification): LogitBoost** (radiomics-based)	Classify atypical **cartilaginous tumors** and **appendicular chondrosarcomas**	Desktop-based	CADx	Scikit-Learn	NR	NR
Gitto [103] ^a^	**Diagnosis** (classification): **ExtraTrees** (radiomics-based)	Determine the diagnostic performance of MRI radiomics-based machine learning in differentiating atypical **cartilaginous tumors** and grade II **chondrosarcomas** of long **bones**	Desktop-based	CADx	Scikit-Learn	https://github.com/rcuocolo/mri_act_cs2	SHAP
Han [87] ^a^	**Diagnosis** (classification): **CNN** (DenseNet-121; Faster R-CNN + deep classification network)	Distinguish cancerous from non-cancerous conditions of the **skin** (binary classification) and predict exact diagnosis (multiclass classification)	Web-based	CADx	Python (Unspecified Package)	https://rcnn.modelderm.com; https://github.com/whria78/modelderm_rcnn_api	NR
He [102]	**Diagnosis** (classification: **CNN** (EfficientNet-B0, pre-trained)	Binary classification: benign versus not-benign and malignant versus non-malignant triple-way classification: benign versus intermediate versus malignant (**bone** cancer)	Desktop-based	CADx	PyTorch 1.6 (Python 3.7)	NR	NR
Hindocha [92] ^b^	**Recurrence** (classification): **kNN + NB + RF** **Recurrence-free survival** (classification): **kNN** **Overall surviva**l (2Y, classification): **XGBoost + ANN + MDA**	Predict recurrence, recurrence-free survival, and overall survival at two years from treatment for non-small-cell **lung** cancer	Desktop-based	CDSS	R Packages Mda, Nnet, Xgboost	https://github.com/orgs/cancer-imaging-group/dashboard	NR
Huang [67]	**Diagnosis** (classification): **CNN** (ResNet) + **RNN** **Cancer-specific surviva**l (regression): **MLP**	Distinguish **gastric** cancer images from normal gastric tissue images and predict the prognosis of **gastric** cancer patients	Web-based	CDSS	Scikit-Learn	https://baigao.github.io/Pathologic-Prognostic-Analysis/	NR
Huang [72] ^b^	**Cancer-specific surviva**l (regression): deep learning survival neural network (DeepSurv, **MLP**)	Predict **esophageal** cancer-specific survival	Desktop-based	CDSS	Python (Version 3.6.7)	NR	NR
Ji [107] ^b^	**Outcome prediction and overall survival** (classification): **XGBoost**	Assess the risk of **kidney cancer bone metastasis** (diagnosis) and prognosis (three-year survival)	Web-based	CADx	Scikit-Learn	https://lryoxidkghwqls-survival-three-years-streamlit-app-f21wz1.streamlit.app/	SHAP
Ji [84] ^ab^	**Outcome prediction and survi**val (classification): XGBoost	Predict the risk of osteoporosis, relative fracture and prognosis for **breast cancer** patients	Web-based	CDSS	Python [Unknown Details]	https://share.streamlit.io/lry4000/survival_8/main	SHAP
Jiang [68] ^b^	**Recurrence** (classification) and **recurrence-free surviva**l (regression): CNN (unknown architecture)	simultaneous prediction of peritoneal recurrence (from gastric cancer) and disease-free survival using preoperative CT images	Desktop-based	CDSS	NR	NR	Grad-CAM
Keyl [76] ^a^	**Overall survival** (regression): **random survival forest**	Overall survival prediction in patients with unresectable advanced **pancreatic** ductal adenocarcinoma at the time of diagnosis	Desktop-based	CDSS	Scikit-SurvivaL (Python 3.8)	NR	SHAP
Kiani [75]	**Diagnosis** (classification): **CNN** (DenseNet)	Distinguish between hepatocellular carcinoma and cholangiocarcinoma	Web-based	CADx	TensorFlow	https://github.com/stanfordmlgroup/lca-code	Grad-CAM
Kim [66] ^a^	**Diagnosis** (binary classification): single **CNN** (unspecified architecture) **Diagnosis** (triple classification: double **CNN** (unspecified architecture)	Differentiate **gastrointestinal** stromal tumors (GISTs) from benign mesenchymal tumors such as leiomyomas and schwannomas observed during endoscopic ultrasonography	Desktop-based	CADx	NR	NR	NR
Kim [108]	**Detection and diagnosis** (classification): **CNN** (YOLOv3, pre-trained)	Detect and classify **liver** metastasis in cancer workup settings	Desktop-based	CADx	PyTorch	https://github.com/pjreddie/darknet	NR
Kudo [61]	**Diagnosis** (classification): **SVM**	Identify **colon** neoplasms from endoscopic images (distinguish neoplastic from non-neoplastic lesions)	Desktop-based	CADx	TensorFlow	NR	NR
Lee [109] ^ab^	**Diagnosis** (classification): **CNN** (Xception)	Diagnosis of cervical lymph node metastasis from **thyroid** cancer with CT imaging	Web-based	CADx	Keras; TensorFlow (Python)	http://ct.cdss.co.kr/	Grad-CAM
Lee [77]	**Overall and recurrence-free survival** (classification): preoperative clinical information models (**ANN + logistic regression + RF + gradient boosting + SVM**) + CT-based models (**CNNs**: 3D ResNet-18, R(2 + 1)D-18, 3D ResNeXt-50, and 3D DenseNet-121)	Predict the **median 2-year overall survival and 1-year recurrence-free survival** using routinely acquired preoperative data for **pancreatic** ductal adenocarcinoma	Desktop-based	CDSS	NR	NR	Grad-CAM
Leibig [85]	**Diagnosis** (classification): **CNN** (unknown architecture, pre-trained)	Identify whether a mammogram image contains signs of **breast** cancer	Desktop-based	CADx	Python (V 3.8.10)	NR	NR
Li [101] ^a^	**Diagnosis** (classification): **CNN** (unknown architecture, pre-trained)	Detect **nasopharyngeal** malignancies	Web-based	CADx	SPSS 22.0	http://nasoai.sysucc.org.cn/	NR
Li [88] ^ab^	**Diagnosis** (classification): **CNN** (DenseNet-121; Faster R-CNN + deep classification network, pre-trained)	Locate **eyelid** tumors and then distinguish between malignant and benign eyelid tumors	Desktop-based	CADe and CADx	PyTorch (1.6.0)	https://github.com/jiangjiewei/EyelidTumors-Source	Grad-CAM
Li [74]	**Overall survival** (3Y, regression): XGBoost + RF + GBDT	Predict overall survival of ICC patients for **three years** after surgical resection	Desktop-based	CDSS	Scikit-Learn (Python 3.6.8)	NR	NR
Lu [94] ^a^	**Risk stratification** (classification): **CNN** (InceptionV4, pre-trained)	Predict 12-year **lung** cancer incidence for high-risk smokers	Desktop-based	CDSS	PyTorch (V. 1.5.1, Python 3.7.7)	https://github.com/circ-ml/CXR-LC	NR
Mehta [82]	**Diagnosis** (classification, **CNN** (Zone-UNet + CSPCa-U-Net))	Automated reporting of **prostate** MRI images to aid in the diagnosis and management of prostate cancer	Web-based	CADx	Streamlit (Version 0.75.0) [Python]	NR	NR
Nam [90] ^a^	**Detection and diagnosis** (classification): **CNN** (unknown architecture)	Detection and classification of malignant **pulmonary** nodules on chest radiographs	Desktop-based	CADe and CADx	NR	NR	NR
Naso [100]	**Diagnosis** (classification): **CNN** (ResNet18) + **RNN**	Distinguish sarcomatoid malignant mesotheliomas from benign spindle cell **mesothelial** proliferations	Desktop-based	CADx	PyTorch	NR	NR
Osman [59] ^ab^	**Survival prediction** (classification): **LightGBM**	Predict **1-, 2-, 3-, 4-, 5-, 6-, 8- and 10-year** postoperative survival of patients with **colorectal** cancer	web-based	CDSS	Light-GBM 2.2 (Python 3.7)	http://colorectalcancer.pythonanywhere.com/	NR
Romeo [86] ^a^	**Diagnosis** (differential classification): **RF** (radiomics-based)	Differential diagnosis of benign and malignant non-cystic **breast** lesions	desktop-based	CADx	Scikit-Learn	NR	NR
She [93] ^b^	**Cancer-specific survival prediction** (regression): deep learning survival neural network (**MLP, DeepSurv**)	Predict survival and test the reliability of individual treatment recommendations for non-small cell **lung** cancer	desktop-based	CDSS	Vue (JavaScript)	https://github.com/thoraciclang/Deep_Lung	NR
Stadlbauer [57] ^ab^	**Diagnosis** (classification): **AdaBoost** (radiomics-based) **Diagnosis** (classification): **RF** (radiomics-based)	Classify contrast-enhancing **brain** tumors	desktop-based	CADx	Weka	NR	NR
Tang [70] ^a^	**Diagnosis** (classification): **CNN** (ResNet50)	Distinguish intramucosal from advanced **gastric** cancer in real-time	desktop-based	CADx	NR	NR	NR
van der Putten [73] ^b^	**Detection and location** (classification): CNN (unknown architecture, pre-trained)	Detection and localization of dysplasia in **Barrett's Esophagus**	desktop-based	CADe	NR	NR	NR
Varghese [81] ^b^	**Incidence risk stratification** (classification): **QSVM** (radiomics-based)	Risk stratification for **prostate** cancer in low- and high-risk patients	desktop-based	CDSS	MATLAB's Classification-Learner Package	NR	NR
von Schacky [105] ^ab^	**Diagnosis** (classification): **ANN** (radiomics-based)	Distinguish between benign and malignant **bone** tumors	desktop-based	CADx	Scikit-Learn (V. 0.22.2); Fastai [Python 3.7.7]	NR	NR
Wang [98] ^ab^	**Diagnosis** (classification): CNN (DenseNet, pre-trained)	Differentiate benign and malignant ground-glass **pulmonary** nodules to assist in the determination of surgical intervention	desktop-based	CADx	Scikit-Learn (V 1.0; Python 3.6.8)	NR	NR
Xiong [99] ^a^	**Diagnosis** (classification): CNN (GoogLeNet Inception v3, pre-trained)	Automatically detect **laryngeal** cancer in laryngoscopic images	desktop-based	CADx	TensorFlow	NR	NR
Xu [69] ^b^	**Outcome detection and prediction** (classification): SVC	Early detection and prediction of pathological downstaging with neoadjuvant chemotherapy in advanced **gastric** cancer	desktop-based	CDSS	NR	NR	NR
Yamada [63] ^ab^	**Diagnosis** (classification): **CNN** (ResNet152)	Prediction of the pathology of early-stage **colorectal** cancers and precursor lesions (differentiation between neoplastic and non-neoplastic lesions)	desktop-based	CADx	NR	NR	NR
Yang [95] ^b^	**Survival risk prediction** (classification): **neural multitask logistic regression model** (deep neural network)	Survival risk stratification in patients with stage III non-small cell **lung** cancer	desktop-based	CDSS	PyTorch	NR	NR
Yang [64] ^ab^	**Diagnosis** (classification): **CNN** (ResNet152, radiomics-based, pre-trained)	Automatically classify **colorectal** lesions histologically on white-light colonoscopy images (neoplastic vs. non-neoplastic and advanced colorectal lesions vs. non-advanced colorectal lesions)	desktop-based	CADx	PyTorch	NR	CAM
Zachariah [106] ^a^	**3-month mortality** (classification): **GBDT**	Prediction of 3-month mortality in patients with **solid metastatic tumors** for several types of cancer (**breast, gastrointestinal, genitourinary, lung, rare**) and treatment (alterations in treatment versus no alterations) in an outpatient setting	desktop-based	CDSS	NR	NR	NR
Zhang [110] ^ab^	**Outcome prediction** (classification): **XGBoost**	Assess the risk of sentinel lymph node metastasis (SLNM) in **breast** cancer patients	desktop-based	CDSS	Scikit-Learn; PyTorch (Python 3.8)	NR	SHAP
Zhang [60]	**Outcome prediction** (classification): **CNN** (unknown architecture)	Preoperatively predict pathologic complete response to neoadjuvant chemoradiotherapy, assess tumor regression grade, and predict T downstaging for **colorectal** cancer patients	desktop-based	CDSS	Keras 2.1.5; TensorFlow 1.4.0 (Python 3.6)	https://github.com/radiologypkucancer/rectal_MR_DL/	NR
Zhang [79]	**Outcome prediction** (classification): **CNN** (FGP-Net)	Predict muscular invasiveness of **bladder** cancer	desktop-based	CDSS	NR	NR	NR
Zhang [56] ^a^	**Diagnosis** (classification): **RF** (radiomics-based)	Distinguish low-grade **gliomas** from **glioblastoma** using preoperative contrast-enhanced MRI scans (**brain** cancer)	desktop-based	CADx	Sklearn.Ensemble (SciKit-Learn, Python)	https://github.com/pwesp/random-forest-polyp-classification	NR
Zhao [83] ^ab^	**Diagnosis** (classification): **CNN** (G2LNet + DenseNet*2, pre-trained)	Diagnose different **endometrial** hyperplasia and screen endometrial intraepithelial neoplasia in endometrial histological images stained by hematoxylin and eosin	desktop-based	CADx	NR	NR	Grad-CAM
Zhu [65] ^b^	**Outcome prediction** (classification): **CNN** (DC3CNN)	Predict pathological tumor regression grade (TRG) response in patients with **colorectal liver metastases** (CRLM) undergoing preoperative chemotherapy	desktop-based	CADx	Keras V 2.1.5; TensorFlow 1.4.0 (Python 3.6)	NR	NR

*ANN* Artificial neural network, *CADe* Computer-aided detection, *CADx* Computer-aided diagnosis, *CDSS* Computerized decision support system, *CNN* Convolutional neural network, *CT* Computed Tomography, *GB* Gradient Boosting, *GBDT* Gradient Boosted Decision Trees, *GRAD-CAM* Gradient-weighted Class Activation Mapping, *EGFR-TKIs* Epidermal growth factor receptor tyrosine kinase inhibitors, *kNN* k-nearest Neighbors, *LightGBM* Light Gradient-Boosting Machine, *MDA* Mixture discriminant analysis, *MLP* Multilayer perceptron, *MRI* Magnetic resonance imaging, *NB* Naïve Bayes, *NR* Not Reported, *QSVM* Quadratic SVM, *RF* Random Forests, *RNN* Recurrent Neural Network, *SHAP* SHapley Additive exPlanations, *SVC* Support Vector Classifier, *SVM* Support Vector Machine, *XGBoost* Extreme gradient boosting

^a^Method explicitly stated in abstract

^b^Authors compared their system with other ML methods

**Fig. 3:**
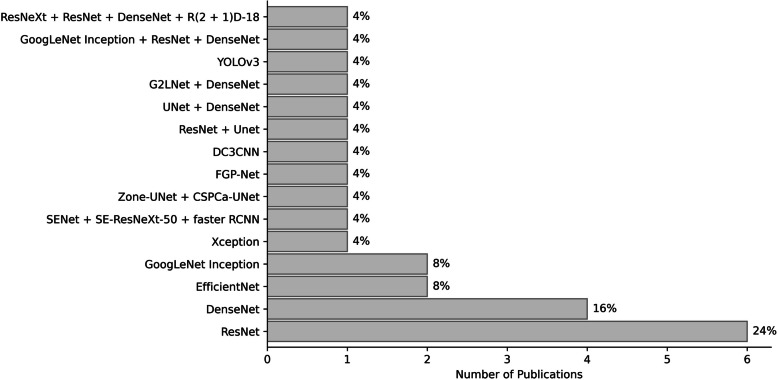
CNN architectures reported in the included papers

Most studies were deep-learning based (*n* = 36, 64.3%). From these, the most frequently reported models were Convolutional Neural Networks (CNNs), either alone (29/36, 80.55%), coupled with a Recurrent Neural Network (RNN, 3/36, 8.34%) [67, 80, 100], or with Logistic Regression (LR), a shallow ANN, Gradient Boosting (GB), a Support Vector Machine (SVM), and Random Forest (RF, 1/36, 2.78%) [77]. Specific CNN architectures were reported for approximately 76% of the articles (25/33), which, as shown in Fig. 3, primarily consisted of ResNet- (*n* = 9, 36%) and DenseNet-based frameworks (*n* = 8, 32%), used individually or in conjunction.

To overcome data scarcity, transfer learning was used in 16 of the 33 CNN-based articles 48.5%), which involves pre-training the network on a specific problem and transferring that base knowledge to a new, related task [116] (see Table 2: *pre-trained* in column *General Focus and Models*).

Besides CNNs, other DL algorithms were described in four articles [67, 72, 93, 95]. Multilayer Perceptrons (MLPs) were used in three (5.56%) [67, 72, 93], two of which applied a DeepSurv architecture, a deep Cox proportional hazards feed-forward neural network [72, 93]. The last (2.78%) involved a neural multitask logistic regression model (N-MTLR) [95].

The remaining documents (*n* = 20, 35.7%) described a non-deep-learning-based workflow encompassing fifteen unique algorithms applied in twenty-eight configurations. From these, boosting-based techniques were the most widely reported, consisting of eXtreme Gradient Boosting (XGBoost, 6/28, 21.43%) [74, 84, 91, 92, 107, 110], a Light Gradient Boosting Machine (LightGBM, 1/28, 3.57%) [59], LogitBoost (1/28, 3.57%) [104], Adaptive Boosting (AdaBoost, 1/28, 3.57%) [57], and Gradient-Boosted Decision Trees (GBDT, 2/28, 7.14%) [74, 106]. Other decision tree designs were also used, including RF (6/28, 21.43%) [74, 84, 91, 92, 107, 110] and extremely randomized trees (ExtraTrees, 1/28, 3.57%) [103]. The third most reported group of algorithms were SVMs [61, 78], a Support Vector Classifier (SVC) [69], and a Quadratic SVM [81] (4/28, 14.28%), followed by shallow ANNs (2/28, 7.14%) [92, 105] and LR (1/28, 3.57%) [115]. Lastly, Mixture Discriminant Analysis (MDA), k-nearest Neighbors (kNNs), and naïve Bayes (NB) were also found, all used in the same article (total of 3/28, 10.71%) [92].

Regarding general cancer types, the selected papers can be broadly divided into two categories: those concentrating on primary tumors and those mainly examining metastasized (secondary) cancers. Most articles focused on primary tumors (51/56, 91.1%), although four also included metastases [57, 58, 76, 90]. These cancers can be further branched into the specific system where the malignancy was formed: (i) central nervous system (CNS), including the brain (3/51, 5.88%) [56–58]; (ii) digestive system, encompassing colorectal (7/51, 13.73%) [59–65], esophageal [71–73] (3/51, 5.88%), gastric (5/51, 9.8%) [66–70], and liver cancers (2/51, 3.92%) [74, 75]; (iii) endocrine system, involving cancers of the pancreas (2/51, 3.92%) [76, 77] and thymus (1/51, 1.96%) [78]; (iv) genitourinary system, consisting of bladder (1/51, 1.96%) [79], cervical (1/51, 1.96%) [80], prostate (2/51, 3.92%) [81, 82], and endometrial (2/51, 3.92%) [83, 115] cancers; (v) integumentary system, with tumors of the breast (4/51, 7.84%) [84–86] and skin [87, 88] (2/51, 3.92%); (vi) respiratory system, studying neoplasms of the larynx (1/51, 1.96%) [99], lung (10/51, 19.61%) [89–98], mesothelium (1/51, 1.96%) [100], and nasopharynx (1/51, 1.96%) [101]; and (vii) the skeletal system, comprising the bones (4/51, 7.84%) [102–105]. In addition, five papers analyzed metastatic cancers (5/56, 8.9%), which can also be bifurcated into malignancies spread to nodes or organs. The former includes solid metastatic breast, lung, and gastrointestinal and genitourinary tract tumors [106], bone metastases in kidney cancer patients [107], and liver metastases from colorectal cancers [108]. The latter encompasses thyroid cancer spread to lymph nodes [109] and sentinel lymph node metastasis from primary breast lesions [110].

Seventy-six cancer-related goals were addressed in the 56 documents, with an average of one task performed per paper and a maximum of three [60, 80, 92]. These included the development or improvement of systems for: (i) diagnosis alone (*n* = 28, 50%) or combined with detection (*n* = 5, 8.93%) [58, 80, 88, 90, 108] or prognosis (*n* = 1, 1.79%) [67]; (ii) detection by itself (*n* = 2, 3.58%) [71, 73] or coupled with outcome prediction (*n* = 1, 1.79%) [69]; and (iii) outcome prediction, including prognosis (*n* = 16, 28.58%) [59, 60, 65, 68, 72, 74, 76, 77, 79, 84, 89, 92, 93, 97, 106, 110]; and risk stratification (*n* = 3, 5.36%) [81, 94, 95].

Finally, fifteen studies resorted to explainable AI (XAI) to increase the transparency behind the models' decisions. Unlike black-box methods, whose reasoning is indecipherable, XAI allows the creation of interpretable models to determine how each prediction was reached and which clinical predictors bore the most weight. Three packages were used for this purpose: (i) SHapley Additive exPlanations (SHAP), which can be employed in any ML algorithm (*n* = 6, 40%) [76, 84, 91, 103, 107, 110]; and (ii) Class Activation Mapping (CAM, *n* = 1, 6.67%) [64] and Gradient-weighted CAM (Grad-CAM, *n* = 8, 53.33%) [62, 68, 75, 77, 83, 88, 89, 109], explicitly developed for CNNs.

#### Clinical inputs and populations

According to the clinical variables used as input, the models validated in the 56 studies can be divided into three types: image-based (including video, *n* = 37, 66.1%), text-based (*n* = 10, 17.9%), and mixed, using both clinical modalities (*n* = 9, 16.1%).

##### Image-based Studies

A total of 335 085 high-resolution images from 112 538 patients^1^ (102 117 female, 8 215 male^2^) were used for classification in 36 of the 37 image-based studies and for classification (recurrence) and regression (recurrence-free survival) in the last study [68]. Except for one paper including both pediatric and adult patients (unknown age proportion, 175 female, 116 male) [102] and two other articles not listing the patients’ age group (698 in [109], unknown in [62], unidentified male–female ratio in both), all studies consisted of adults (111 469 patients, 101 942 women, 8 099 men). Eight studies (21.6%) extracted radiomic features from the retrieved images [56, 57, 64, 69, 81, 86, 103, 104]. The studies encompassed X-rays, Computed Tomography (CT), Magnetic Resonance Imaging (MRI), Positron Emission Tomography – Computed Tomography (PET-CT) scans, endoscopic images and videos, photographs, ultrasounds, histological slides, and whole-slide images (WSI). Besides digital pictures, which are limited to the surface, these imaging techniques capture the body's internal structures. However, they differ in the way they create images and the type of information they provide.

X-rays use and expose the patient to ionizing radiation to create scans [117]. Although time- and cost-effective, these do not provide as much detail as CT or MRI scans. In this review, two studies used radiographic images (2/37, 5.4%) to: (i) classify pathologically-confirmed primary bone tumors in children and adults (639 radiographs, 175 female, 116 male) [102]; and (ii) for breast cancer screening in adult women (*n* = 1, 213 694 X-rays, 92 585 women) [85].

CT scans combine X-rays from different angles to create high-quality, three-dimensional images. Nevertheless, since they are generated from controlled motions of X-rays, CTs are still unfit for extracting molecular information [118]. Furthermore, these scans subject the patient to higher radiation levels than X-rays [117] and may require contrast agents depending on the adopted modality – contrast-enhanced CTs (CECTs) versus non-contrast CTs (NECTs). CT scans were commonly collected variables in the selected articles (8/37, 21.6%), amounting to 7 540 images from: (i) the lungs (*n* = 4, 2 323 nodules, 2 113 patients) [89, 90, 96, 97]; (ii) gastric cancers (*n* = 2, 1 129 images, 352 women, 777 men) [68, 69]; (iii) cervical lymph nodes (*n* = 1, 3 838 images, 698 patients of unknown gender) [109]; and (iv) hepatic metastasis from colorectal cancer (*n* = 1, 250 lesions, 31 women, 54 men) [108].

MRI scans do not depend on radiation and use a strong magnetic field and radio waves to create detailed images. This type of imaging can be separated into two subtypes: conventional and advanced [118]. Conventional MRI (cMRI) sequences include standard MRI protocols commonly used in clinical practice, such as (i) T1-weighted: used to identify structural abnormalities; (ii) Axial fluid-attenuated inversion recovery MRI (FLAIR), applied to identify abnormalities that affect the tissues' water content; and (iii) T2-weighted: also appropriate to assess irregularities in water content. Advanced MRI (advMRI) techniques generate deeper information regarding the tissue's function, structure, and metabolic processes, including: (i) multiparametric MRI (mpMRI), which combine several other MRI sequences to enrich its output; (ii) axial diffusion-weighted (DWI) MRI, which measure the movement of water molecules in tissues; (iii) Vascular architecture mapping (VAM) MRI, providing information about the tissue's blood vessels; (iv) Gradient echo dynamic susceptibility contrast (DSC) MRI, used to measure blood movement; (v) Quantitative blood-oxygenation-level-dependent (qBOLD) MRI, able to measure the oxygen content in the blood; (vi) General Electric-Dynamic Susceptibility Contrast (GE-DSC) MRI, which resorts to a contrast agent to measure blood flow; and (vii) Magnetic resonance spectroscopy (MRS), which calculate the levels of certain chemicals and metabolites in the tissues. Although some types of MRIs – such as MR spectroscopy and diffusion-weighted imaging – allow assessing molecular details without contrasts, most are better equipped to analyze gross internal structures and are more expensive than CTs and X-rays [118]. MRI scans were also frequently used as input for the models, with 64 941 combined images from 8 studies (21.6%), including (i) the brain (*n* = 3, 64 459 lesions, 623 women, 461 men) [56–58]; (ii) the prostate (*n* = 2, 262 nodules, 300 men) [81, 82]; (iii) colorectal malignancies (*n* = 2, 154 images, 54 women, 64 men) [60, 65]; and (iv) bones and cartilages (*n* = 1, 65 scans, 34 women, 31 men) [103].

PET scans, which are also radiation-free, allow for examining the internal body structure and underlying molecular tissues. However, these are extremely expensive, usually unavailable in routine practice, and due to their low spatial resolution, require pairing with a second modality, such as CTs and MRIs [118]. In this review, one study (2.7%) used PET-CT scans to examine atypical cartilaginous tumors and appendicular chondrosarcomas (36 scans, 23 women, 13 men) [104].

Similarly to X-rays, ultrasounds – which use high-frequency sound waves to create images – provide an inexpensive method to inspect organ structures without detailing underlying molecular information, with the upside of not involving radiation [118]. Ultrasonographic imaging was mentioned in 2 articles (*n* = 2, 5.4%, 328), which studied breast cancers (116 ultrasounds, 107 women) [86, 110].

Eight reports describe images captured with standard endoscopes (*n* = 8, 24.3%, 3681 items), which cannot capture molecular features. Four studies used colonoscopic lesions from the colon and rectum (995 images, 105 women, 224 men) [61–64]. Four studies analyzed endoscopic pictures of the esophagus (*n* = 2, 260 images, 260 patients of unknown gender) [71, 73], the larynx (*n* = 1, 1 176 images, unknown number of patients) [99], and the nasopharynx (*n* = 1, 1 430 images, 124 women, 231 men) [101]. Lastly, one study examined endoscopic videos from intramucosal gastric cancer patients (54 videos, 38 women, 16 men) [70].

Two studies used advanced endoscopes. One involved endoscopic ultrasonography (EUS), a technique that combines endoscopy and ultrasonography to gather gastrointestinal images (*n* = 1, 2.7%, 212 ultrasounds, 38 women, 31 men) [66]. The other resorted to endocytoscopy, a relatively new high-magnification imaging approach that allows tissue analysis at a cellular level, to collect 100 colorectal images from 89 patients (*n* = 1, 2.7%, 26 women, 63 men) [61].

A histological image is a high-resolution, microscopic image of a tissue slide after it's been processed with one or more stains to reveal its composition [119]. This method allows distinguishing between different histological cancer subtypes but involves a long preparation time and offers a limited depth of view. One paper used hematoxylin-and-eosin (H&E)-stained histological images to study endometrium hyperplasia and intraepithelial neoplasia (*n* = 1, 2.7%, 1 631 slides, 102 women) [83].

Whole-slide images (WSIs) are virtual representations of a tissue section scanned at high resolution and magnification. WSIs are created by scanning stained histological slides and usually combine and magnify multiple slides using specialized software [120]. This technique allows thorough tissue examination at cellular and sub-cellular levels, but it is still cost-, storage- and technically heavy. WSIs were used to feed the models in three studies (8.1%, 3 315 images), using 30 × or 40 × magnification. Two included H&E stained slides of the liver (*n* = 1, 80 slides, 24 women, 56 men) [75] and the mesothelium (*n* = 1, 39 images, 39 patients of unreported gender) [100]. One was composed of stained slides (unknown stain) for the cervical screening of women without any known conditions and with the Human papillomavirus (HPV) (*n* = 1, 1565 images and women) [80].

Finally, 46 962 digital photographs (captured with a camera) were analyzed across two documents (5.4%). Both inspected skin malignancies (*n* = 2, 10 602 patients).

Detailed information regarding the samples, type of CTs, MRIs, and endoscopes used in the image-based studies, as well as population details and counts (age group, total patients, female, and male), is itemized in Table 3.

**Table 3:** Population details and sample types for the 37 selected image-based studies

Citation	Population Details	Age Group	#patients	Sample Type	Sample Size
Baldwin [96]	Patients with incidentally detected **pulmonary** nodules measuring 5–15 mm	Adults	1187	CECT and NECT images	1 397
Cheng [80]	Women without any known conditions and HPV-positive women	Adults	1 565♀	WSIs: stained slides (with unknown stain) magnified 40 times	1 565
Choi [89]	Patients with resected clinical stage IA **lung** adenocarcinomas	Adults	141 (84♀,57♂)	CECT images (preoperative)	141
Choi [62]	Patients' standard white light **colonoscopic** tumor images that had been histologically confirmed and removed with en-bloc resection	NR	NR	White-light colonoscopy images	400
De Groof [71]	Patients with Barrett’s **esophagus** (BE) and nondysplastic BE with rigorously confirmed early-stage neoplasia	Adults	160	White-light endoscopic images	160
Deng [97]	Patients with stage IV EGFR-mutant non-small cell **lung** cancer treated with EGFR-tyrosine kinase and immune checkpoint inhibitors	Adults	92 (52♀,40♂)	CT images (pre-treatment, unknown if CECT or NECT)	92
Gao [58]	Patients with a pathologically confirmed, single **brain** tumor with the availability of pretreatment MRI scans	Adults	1 039 (600♀, 439♂)	T1-weighted, T2-weighted, T1c MRI scans (pretreatment)	64 414
Gitto [104]	Patients with atypical **cartilaginous** tumors or appendicular chondrosarcoma (**bone**)	Adults	36 (23♀, 13♂)	Preoperative PET-CT scans (unknown if CECT or NECT)	36
Gitto [103]	Patients with pathologically confirmed atypical **cartilaginous** tumors or grade II chondrosarcoma of long **bones** with unavailable MRI scan	Adults	65 (34♀, 31♂)	T1-weighted MRI scans	65
Han [87]	Pathologically diagnosed patients with 1 of 43 primary **skin** neoplasms of the head and neck, trunk, arms, and leg > s	Adults	10,426 (5595♀, 5281♂)	Photographs	40 331
He [102]	Patients with pathologically-confirmed primary **bone** tumors and available pre-procedure radiograph	Children and Adults	291 (175♀, 116♂)	X-rays (preoperative, uncropped)	639
Jiang [68]	Patients with gastric cancer who underwent abdominal CECT scans before gastrectomy	Adults	1043 (329♀, 714♂)	CECT images (preoperative)	1043
Kiani [75]	Digital WSI of H&E-stained slides from primary **hepatic** tumor resections from patients with a pathologic diagnosis of hepatocellular carcinoma or cholangiocarcinoma	Adults	80 (24♀, 56♂)	WSI: eosin-stained (H&E) slides magnified 40 times	80
Kim [66]	Patients with a histopathologically confirmed diagnosis of **gastric** mesenchymal tumors (GISTs, leiomyomas, or schwannomas)	Adults	69 (38♀, 31♂)	Endoscopic ultrasonography images	212
Kim [108]	Recently diagnosed **colorectal** adenocarcinoma patients who underwent pretreatment contrast-enhanced abdominopelvic CT	Adults	85 (31♀, 54♂)	CECT images (pretreatment)	250
Kudo [61]	Patients with small neoplastic and non-neoplastic **colorectal** polyps (< 10 mm) confirmed on colonoscopy using an endocytoscope	Adults	89 (26♀, 63♂)	Stained endocytoscopic images	100
Lee [109]	Patients with **thyroid** cancer	NR	698	CECT images (preoperative)	3838
Leibig [85]	Patients with screen-detected **breast** cancers and follow-up-proven normal mammography exams	Adults	92,585♀	Mammographic images (X-rays)	213,694
Li [101]	Patients who underwent routine clinical screening for **nasopharyngeal** malignancies	Adults	355 (124♀, 231♂)	White-light endoscopic images	1430
Li [88]	Patients who presented for eye examinations and ophthalmology consultations due to the discovery of **eyelid** tumors	Adults	176 (111♀, 65♂)	Photographs (images)	266
Mehta [82]	Whole-prostate and zonal segmentations for men who had undergone an initial standard transrectal ultrasound-guided or transperineal template **prostate**-mapping biopsy, but concern remained over the accuracy of the subsequent diagnosis	Adults	247♂	mpMRI scans	210
Nam [90]	Patients with malignant **pulmonary** nodules with available chest radiography and referential chest cts	Adults	693 (295♀, 398♂)	CECT images	693
Naso [100]	Patients with a pathologically confirmed diagnosis of sarcomatoid malignant mesotheliomas or benign spindle cell **mesothelial** proliferations	Adults	39	WSIs: eosin-stained (H&E) slides magnified 40 times	39
Romeo [86]	Patients with **breast** lesions who underwent breast ultrasound examinations	Adults	57♀	Ultrasound images	66
Stadlbauer [57]	Newly diagnosed patients with untreated contrast-enhancing **brain** tumors with available clinical routine MRI data	Adults	20 (11♀, 9♂)	cMRI: FLAIR, T1-weighted advMRI: GE-DSC perfusion, DWI phyMRI: VAM-MRI, qBOLD	20
Tang [70]	Patients who underwent endoscopic submucosal dissection or gastrectomy with histologically proven intramucosal **gastric** malignancies	Adults	54 (38♀, 16♂)	Endoscopic videos	54
Van der Putten [73]	General cases of a wide variety of esophageal lesions (lesions representative in clinical practice) and visually subtle esophageal lesions from patients with Barrett's neoplasia	Adults	180	Endoscopic images	180
Varghese [81]	Patients with a histopathologic diagnosis of **prostate** cancer, with mpMRI of the prostate, and transrectal ultrasound-MRI fusion guided biopsy of the prostate within 2 months of mpMRI	Adults	53	mpMRI scans	53
Xiong [99]	Raw **laryngoscopic** images	Adults	NR	White-light endoscopic images	1176
Xu [69]	Patients with histologically confirmed **gastric** cancer and absence of distant metastases on admission, who underwent 1–4 cycles of neoadjuvant chemotherapy followed by gastrectomy with lymph node dissection	Adults	86 (23♀, 63♂)	CECT images (pre-treatment)	86
Yamada [63]	Patients who underwent colonoscopy for fecal immunochemical testing positive for **colorectal** cancer, and surveilled after polypectomy	Adults	NR	White-light, narrow-band, and blue-laser colonoscopy images	255
Yang [64]	Patients with diagnosed **colorectal** cancer	Adults	240 (79♀, 161♂)	White-light colonoscopy images	240
Zhang [110]	Patients with primary **breast** cancer who were first discovered and had no history of other malignancies	Adults	50♀	Ultrasound images	50
Zhang [60]	Patients with locally advanced **rectal** adenocarcinoma proved with histopathology and baseline MRI (> = ct3 or N +) and scheduled to undergo neoadjuvant chemoradiotherapy	Adults	93 (41♀, 52♂)	Diffusion kurtosis and T2-weighted MRI scans	93
Zhang [56]	Glioblastoma and low-grade glioma collections (**brain** cancer) that were identified, selected, and labeled by expert board-certified neuroradiologists	Adults	25 (12♀, 13♂)	T1-weighted and T2-weighted MRI scans (preoperative)	25
Zhao [83]	**Endometrial** specimens collected by diagnostic curettage, hysteroscopic surgery or hysterectomy from female patients who did not receive radiotherapy, chemotherapy or hormone therapy before surgery and had no other gynecologic tumor complications (uterine cancer)	Adults	102♀	H&E-stained histological images	1631
Zhu [65]	Patients with pathologically confirmed **liver** metastases and no more than five liver metastatic lesions who were scheduled to receive preoperative chemotherapy followed by liver resection	Adults	25 (13♀, 12♂)	T2-weighted- and DW-MRI scans (pre and post-treatment)	61

If available, the number of female and male patients are presented in parentheses in column Patients

*advMRI* Advanced MRI, *BE* Barrett's Esophagus, *CECT* Contrast-enhanced CT images, *cMRI* Conventional MRI, *CT* Computer Tomography, *DWI* Axial diffusion-weighted imaging, *FLAIR* Axial fluid-attenuated inversion recovery MRI, *GE-DSC* General Electric-Dynamic Susceptibility Contrast, *GISTs* Gastrointestinal stromal tumors, *H&E* Hematoxylin and Eosin, *mp-MRI* Multiparametric MRI scans, *MRI* Magnetic Resonance Imaging, *NECT* Non-contrast-enhanced CT images, *NR* Not Reported, *PET-CT* Positron Emission Tomography – Computed Tomography, *physMRI* Physiological MRI, *qBOLD* Quantitative blood-oxygenation-level-dependent MRI, *T1c* gadolinium-contrast-enhanced T1-weighted MRI, *VAM-MRI* vascular architecture mapping MRI, *WSIs* Whole-Slide Images

##### Text-based Studies

The populations and specific clinical variables used in each text-based study are compiled in Table 4. Clinical data from 6 803 patients (2 772 women, 4 031 men, 7 861 encounters) was collected for validation across ten papers [59, 72, 74, 76, 84, 92, 93, 95, 106, 107]. Apart from one work including senior citizens [92], all studies consisted of adult patients (6 644 subjects, 2 701 women, 3 943 men). An average of 17 clinical variables was used per study (range = 6 [76] – 31 [92]), encompassing information on demographics, tumoral values, and laboratory test results. The machine learning models used in 6 of the articles (60%) were exclusively developed for classification (1 960 women, 3 097 men) [59, 84, 92, 95, 106, 107], while 4 (40%) solely concerned regression (812 women, 934 men) [72, 74, 76, 93].

**Table 4:** Population details and clinical variables used in the ten text-based studies

First Author/ Reference	Population Details	Age Group	# Patients	# Female	# Male	Clinical Variables
Hindocha [92]	Patients with stage I to III disease treated with curative-intent radiotherapy for non-small cell **lung** cancer	Seniors	159	71	88	Sex, age, ethnicity, WHO performance status, smoking status, TNM8 T-stage, TNM8 N-stage, TNM8 overall clinical stage, size of the primary lesion, FDG PET-CT SUV of the primary lesion, nodal avidity and maximal nodal SUV, node sampling, confirmation of pathological diagnosis, histological type, BMI, pre-treatment FEV1 and TLCO, pre and post-treatment neutrophil, and lymphocyte counts, type of radiotherapy treatment received, total dose in Gy, number of fractions, biologically effective dose in Gy, radiotherapy GTV tumor volume and PTV, dates of radiotherapy planning scan, and first and last fraction of radiotherapy
Huang [72]	Patients with pathologically confirmed primary stage I to IV **esophageal** cancer (only adenocarcinoma and squamous cell carcinoma) and the presence of one primary malignant lesion	Adults	500	150	350	Sex, age, race, marital status, primary tumor site and size, histologic grade, histologic type, TNM stage, and treatment details (surgical type)
Ji [107]	**Kidney** cancer patients with complete survival data	Adults	963	323	640	Age at diagnosis, race, gender, primary site, grade, histology subtype, marital status, insurance recode, stage, TNM stage, surgery, lymph node surgery, radiation recode, chemotherapy, brain metastasis, liver metastasis, lung metastasis, laterality, and tumor size
Ji [84]	Patients with primary **breast** cancer diagnosed by pathological examination	Adults	150	150	0	Osteoporosis model: age, BMI, smoking history, history of alcohol intake, M stage, molecular type of primary tumor, surgical treatment, anti-estrogen therapy, chemotherapy, targeted therapy, glucocorticoid medication, radiation therapy, family history of osteoporosis, fracture history, Karnofsky score less than 40, blood BALP level, blood calcium level, and blood phosphorus level fracture model: variables for the osteoporosis models plus history of osteoporosis survival model: age, smoking history, history of alcohol intake, T and N stages, molecular type of primary tumor, surgical treatment, anti-estrogen therapy, chemotherapy, targeted therapy, glucocorticoid medication, radiation therapy, osteoporosis, brain metastasis, liver metastasis, and lung metastasis
Keyl [76]	Patients with unresectable advanced **pancreatic** ductal adenocarcinoma with liver metastases treated palliatively	Adults	22	8	14	Age at diagnosis, metastatic status (no metastases, liver metastases), C-reactive protein, neutrophil-to-lymphocyte ratio, CA19-9 level, and total serum protein level
Li [74]	Patients with pathologically confirmed resectable **intrahepatic** cholangiocarcinoma who underwent hepatectomy	Adults	42	12	30	Age, gender, jaundice, history of kidney stones, history of tumor, smoking status, laboratory test results (blood type, HBV, CA19-9, g-glutamyltranspeptidase, albumin, alanine aminotransferase, CEA, ALP, pre albumin PA, aspartate aminotransferase, AFP, and direct and total bilirubin), perioperative T/N/Mor TNM-8 stage, T or TNM stage in LCSGJ, resection type, and tumor size
Osman [59]	Patients with primary **colorectal** cancer who underwent surgery and had survival-related data	Adults	1572	607	965	Age at diagnosis, sex, tumor location, histology, grade, AJCC stage, tumor size in millimeters, number of examined lymph nodes, number of positive lymph nodes, radiation, chemotherapy, and carcinoembryonic antigen level
She [93]	Patients with stage I to III non-small cell **lung** cancer	Adults	1182	642	540	Sex, age, marriage status, tumor location, size, histologic grade, histologic type, TNM stage, and treatment details (surgical type)
Yang [95]	Patients with newly diagnosed and pathologically confirmed primary stage III non-small cell **lung** cancer	Adults	172	39	133	Year of diagnosis, age, sex, race, T stage, N stage, primary site, laterality, histology, differentiation, number of positive lymph nodes, visceral pleural invasion, surgery to the primary site, chemotherapy, and radiotherapy
Zachariah [106]	Patients with advanced cancer with solid metastatic tumors of the lung, breast, rare, gastrointestinal, and genitourinary with and without changes in treatment	Adults	2041	770	1271	Age, gender, race, diagnosis ICD-9 code, weight, BMI, lymphocyte percentage, albumin, calcium, white blood cells, lactate dehydrogenase, hemoglobin, platelet count, alkaline phosphatase, creatinine, bilirubin, red blood cells, B12, percentage of segmented neutrophils

*AJCC* American Joint Committee on Cancer, *ASA* American Society of Anesthesiologists, *BALP* Bone-specific alkaline phosphatase, *BMI* Body Mass Index, *CEA* Carcinoembryonic antigen, *FEV1* Forced expiratory volume in 1 s, *GTV* Gross Tumor Volume, *HBV* Hepatitis B virus, *ICD* International Classification of Diseases, *PTV* Planning target volume, *SUV* Standard Uptake Value, *TLCO* Diffusing capacity for carbon monoxide, *WHO* World Health Organization

In the four regression-based articles, the developed prognostic models assessed (i) patients with a single lesion of primary stage I to IV esophageal adenocarcinoma or squamous cell carcinoma (*n* = 1, 150 women, 350 men) [72]; (ii) patients with pathologically confirmed and resected intrahepatic cholangiocarcinoma (12 women, 30 men) [74]; (iii) patients with stage I to III non-small cell lung cancer (642 women, 540 men) [93]; and (iv) patients in palliative care with unresectable advanced pancreatic ductal adenocarcinoma with liver metastases (8 women, 14 men) [76].

The six classification papers included: (i) seniors with stage I to III non-small cell lung cancer treated with curative-intent radiotherapy (159 individuals, 71 women, 88 men) [92]; (ii) bone metastasis in kidney cancer patients with complete survival data (323 women, 640 men) [107]; (iii) women with primary breast cancer diagnosed by pathological examination (150 women) [84]; (iv) patients with primary colorectal cancer with survival-related data who underwent surgery (1 572 patients, 607 female, 965 male) [59]; (v) patients with confirmed stage III non-small cell lung cancer (39 women, 133 men) [95]; and (vi) patients with solid metastatic tumors for several types of cancer with and without alterations in treatment in an outpatient setting (3 099 encounters, 2 041 individuals, 770 women, 1 271 men) [106].

##### Mixed Studies

An average of 9 clinical variables (range = 3 [94] – 17 [77]), 784 images, and 720 patients (range = 44 [115] – 5 493 [94] for both) were used in the nine mixed studies, whose information is highlighted in Table 5. These papers combined patients’ demographics, cancer-specific data, laboratory results, and imaging features extracted from different modalities for cancer-specific populations (7 053 images, 6 482 patients, 3 009 women, 3 478 men). Radiomics approaches were used in three studies [78, 105, 115]. Six reports included CT images to study: (i) patients who underwent curative-intent resection for pancreatic ductal adenocarcinoma (*n* = 1, 53 images, 27 women, 26 men) [77]; (ii) patients with benign and malignant pulmonary ground-glass nodules with less than 30 mm (*n* = 1, 63 images, 39 women, 22 men) [98]; (iii) individuals with multiple lungs nodes in a post-operative setting (*n* = 1, 200 images, 51 women, 27 men) [91]; (iv) lung cancer patients with an available baseline radiograph (*n* = 1, 5 493 patients and images, 2456 women, 3037 men) [94]; (v) patients with muscle-invasive bladder cancer who underwent surgery (*n* = 1, 75 images, 13 women, 62 men) [79]; and (vi) adults with pathologically confirmed thymomas and thymic carcinomas (*n* = 1, 76 preoperative scans, 33 women, 48 men) [78].

**Table 5:** Population details, imaging modalities and clinical variables used in the nine mixed studies

First Author/ Reference	Population Details	#Patients^a^	#Women	#Men	#Images	Type of Imaging Modality	Clinical Variables
Bi [115]	Patients with stage IA **endometrial** cancer, endometrial hyperplasia, or endometrial polyps confirmed by histopathology who underwent MR examination within two weeks before treatment and had complete clinical data	44	44	0	44	T1-weighted, T2-weighted, and DWI MRI scans	Histological subtypes, age, menopause, clinical manifestation, metabolic syndrome, BMI, actual treatment options, CA125 and CA199 level, and immunohistochemical findings (estrogen receptor, progesterone receptor, P53, and Ki-67)
Chen [91]	Patients with multiple **pulmonary** nodules who had undergone surgical treatment	493	297	196	978	CT scans (unknown if CECT or NECT)	Age, sex, family history of lung cancer, smoking status, smoking quantity (pack per year), time since quitting smoking, and tumor markers (carcinoembryonic antigen, cytokeratin 19 fragment, neuron-specific enolase, and cancer antigens 199 and 125
Feng [78]	Patients with pathologically confirmed **thymic** epithelial tumors	76	33	48	76	NECT images (preoperative)	Sex, age, clinical symptoms, and smoking status
Huang [67]	Patients unequivocally diagnosed with **gastric** cancer by preoperative biopsy or postoperative pathological examination	91	60	31	175	Whole-slide images: eosin-stained (h&e) slides magnified 30 times	Survival state, overall survival time, age, sex, tumor size, neoplasm histologic grade, and pathologic T, N, M, and TNM-8 stages
Lee [77]	Patients who underwent upfront curative-intent resection for **pancreatic** ductal adenocarcinoma	53	27	26	53	Cect images	Age, sex, ASA score, ca19-9, cea, hemoglobin, white blood cell count, total bilirubin, albumin, tumor location, head/body or tail/head and body/tail, type of pancreatic surgery, resection margin, tumor differentiation, lymphovascular invasion, perineural invasion, and AJCC 8th stage
Lu [94]	Patients with **lung** cancer who were in the chest radiography arm and had an available baseline radiograph	5493	2456	3037	5493	Low-dose CT images (unknown if CECT or NECT)	Age, sex, smoking status
Von Shacky [105]	Patients with primary **bone** tumors	96	40	56	96	X-ray images + clinical data	Age, sex, tumor type, location, and radiomic features (unspecified)
Wang [98]	Patients with pathologically confirmed **pulmonary** ground-glass nodules measuring < 30 mm on specimens obtained by CT-guided transthoracic needle biopsy, transbronchial biopsy, video-assisted thoracoscopic surgery, or surgical resection	61	39	22	63	CT images (unknown if CECT or NECT)	Age, sex, nodule-lung interface, pleural indentation, specular sign, smoking history, nodule attenuation, lobulation, vacuole sign, and air bronchogram
Zhang [79]	Patients who underwent transurethral resection of a **bladder** tumor or radical cystectomy with pathologically confirmed urothelial carcinoma and had performed CT urography	75	13	62	75	CECT images	Age, sex, pathologic T stage, tumor number, size, and CT attenuation of the largest tumor

*AJCC* American Joint Committee on Cancer, *ASA* American Society of Anesthesiologists, *BMI* Body Mass Index, *CEA* Carcinoembryonic antigen, *CECT* Contrast-enhanced CT images, *CT* Computed Tomography, *DWI* Axial diffusion-weighted imaging, *H&E* Hematoxylin and Eosin, *MR* Magnetic Resonance, *MRI* Magnetic Resonance Imaging, *NECT* Non-contrast-enhanced CT images

^a^ All patients were adults (> 18 years)

Additionally, three studies used other types of scans. One work paired breast-specific data with features derived from three types of MRI scans for women with endometrial lesions and complete clinical data (44 images, 44 women) [115]. One paper combined patients’ age, sex, tumor type, location, and radiomic features extracted from X-rays to analyze primary bone tumors (40 women, 56 men) [105]. Finally, one study evaluated survival- and gross-tumor-related data in conjunction with H&E slides magnified 30 times (whole-slide images) to estimate outcomes for patients diagnosed with gastric cancer (175 images, 91 patients, 60 female, 31 male) [67]. Except for the models developed in this study, where the first used only WSIs for classification and the second used these images and clinical data for prognostication (regression), all algorithms were classifiers.

#### Validation design, clinical settings and performance metrics

Information concerning institutional, study, and validation designs, care types, datasets, clinical settings, and the number of institutions involved in validation in the selected documents is illustrated in Table 6. Model development and validation were performed simultaneously in most studies (*n* = 50, 87.5%), while 4 (7.14%) [61, 87, 96, 109] evaluated external validity separately, and 3 (5.36%) [72, 73, 93] entailed model updating and validation. Of the 56 documents included in this review, 44 (78.57%) directly reference external validation in the abstract, 10 (17.86%) indirectly mention it, and 2 (3.57%) omit this information.

**Table 6:** Institutional, study, and validation designs, care types, datasets, clinical settings and the number of institutions involved in validation in the selected documents

First Author/ Reference	Validation Design	Care Type	Study Design	Institutional Design	Training Countries	Validation Countries	Validation Type
Baldwin [96] ^ac^	Validation only	Tertiary	Retrospective	Multi-institutional	USA (NLST)	UK (3)	Temporal; geographical (international); type of CT modality
Bi [115] ^a^	Development and validation	Unknown	Retrospective	Multi-institutional	China	China (1)	Geographical
Chen [91] ^ad^	Development and validation	Tertiary	Prospective and Retrospective	Multi-institutional	China	China (5); South Korea (1)	Temporal and geographical
Cheng [80] ^ac^	Development and validation	Tertiary	Retrospective	Multi-institutional	China	China (5)	Temporal and geographical
Choi [89] ^ac^	Development and validation	Quaternary	Retrospective	Single institution	South Korea	South Korea (1)	Temporal
Choi [62]	Development and validation	Tertiary	Retrospective	Multi-institutional	South Korea	South Korea (1)	Geographical
De Groof [71] ^adc^	Development and validation	Tertiary	Prospective and Retrospective	Multi-institutional	The Netherlands	The Netherlands (2)	Temporal; design of data acquisition
Deng [97] ^a^	Development and validation	Quaternary	Retrospective	Multi-institutional	China	China (3)	Temporal and geographical
Feng [78] ^b^	Development and validation	Tertiary	Retrospective	Single institution	China	China (1)	Temporal
Gao [58] ^ad^	Development and validation	Tertiary	Retrospective	Multi-institutional	China	China (3)	Geographical
Gitto [104] ^ac^	Development and validation	Tertiary	Retrospective	Multi-institutional	Italy	Italy (1)	Temporal and geographical
Gitto [103] ^ac^	Development and validation	Tertiary	Retrospective	Multi-institutional	Italy	The Netherlands	Temporal and geographical (international)
Han [87] ^adc^	Validation only	Quaternary	Retrospective	Multi-institutional and 1 public database	South Korea	South Korea (1); UK (Edinburgh dataset)	Temporal and geographical (international, ethnic)
He [102] ^ac^	Development and validation	Tertiary	Retrospective	Multi-institutional	USA; China	China (2)	Geographical (international, ethnic)
Hindocha [92] ^ac^	Development and validation	Tertiary	Retrospective	Multi-institutional	UK	UK (5)	Geographical
Huang [67] ^a^	Development and validation	Tertiary	Retrospective	2 public databases, 1 institution	China + TCGA	China (NHGRP)	Temporal and geographical (international)
Huang [72] ^af^	Update and validation	Tertiary	Retrospective	1 public database, 1 institution	USA (SEER dataset)	China (1)	Temporal and geographical (international, ethnic)
Ji [107] ^a^	Development and validation	Tertiary	Retrospective	1 public database, 1 institution	USA (SEER dataset)	China (1)	Temporal and geographical (international, ethnic)
Ji [84] ^a^	Development and validation	Quaternary	Retrospective	Multi-institutional	China	China (1)	Geographical
Jiang [68] ^a^	Development and validation	Quaternary	Retrospective	Multi-institutional	China	China (1)	Temporal and geographical
Keyl [76] ^a^	Development and validation	Quaternary	Retrospective	Multi-institutional	Germany	Germany (1)	Temporal and geographical
Kiani [75] ^bc^	Development and validation	Quaternary	Retrospective	1 public database, 1 institution	USA (TCGA)	UK (1)	Temporal and geographical (international)
Kim [66] ^b^	Development and validation	Tertiary	Retrospective	Multi-institutional	South Korea	South Korea (3)	Temporal
Kim [108] ^bc^	Development and validation	Tertiary	Retrospective	Single institution	South Korea	South Korea (1)	Temporal
Kudo [61] ^bd^	Validation only	Tertiary	Retrospective	Multi-institutional	Japan	Japan (5)	Temporal and geographical
Lee [109] ^a^	Validation only	Tertiary	Retrospective	Single institution	South Korea	South Korea (1)	Temporal
Lee [77] ^bc^	Development and validation	Quaternary	Retrospective	Single institution	South Korea	South Korea (1)	Temporal
Leibig [85] ^a^	Development and validation	Unknown	Retrospective	Multi-institutional	Germany	Germany (2)	Temporal and geographical
Li [101] ^ad^	Development and validation	Tertiary	Internal: retrospective; external: prospective	Single institution	China	China (1)	Temporal; design of data acquisition
Li [88] ^a^	Development and validation	Tertiary	Retrospective	Multi-institutional	China	China (2)	Temporal and geographical
Li [74] ^ae^	Development and validation	Quaternary	Retrospective	Single institution	China	China (1)	Temporal
Lu [94] ^ac^	Development and validation	Not applicable	Retrospective	Multi-institutional database	USA (PLCO trial)	USA (NLST) (33)	Temporal and geographical
Mehta [82] ^ac^	Development and validation	Not applicable	Retrospective	2 public databases	The Netherlands (PROSTATEx dataset)	UK (PICTURE dataset)	Temporal and geographical (international)
Nam [90] ^ac^	Development and validation	Tertiary	Retrospective	Multi-institutional	South Korea	South Korea (3); USA (1)	Temporal and geographical (international, ethnic)
Naso [100] ^ac^	Development and validation	Unknown	Retrospective	Multi-institutional	Canada	China (2)	Geographical
Osman [59] ^a^	Development and validation	Tertiary	Retrospective	1 public database, 1 institution	USA (SEER dataset)	South Korea (1)	Temporal and geographical (international, ethnic)
Romeo [86] ^a^	Development and validation	Tertiary	Retrospective	Multi-institutional	Italy	Italy (1)	Temporal and geographical
She [93] ^af^	Update and validation	Tertiary	Retrospective	1 public database, 1 institution	USA (SEER dataset)	China (1)	Temporal and geographical (international, ethnic)
Stadlbauer [57] ^bc^	Development and validation	Tertiary	Retrospective	Single institution	Austria	Austria (1)	Temporal
Tang [70] ^a^	Development and validation	Tertiary	Retrospective	Multi-institutional	China	China (1)	Temporal
Van der Putten [73] ^ac^	Update and validation	Unknown	Prospective	Multi-institutional	Unknown	Unknown	Scanner type
Varghese [81] ^bc^	Development and validation	Quaternary	Retrospective	Single institution	USA	USA (1)	Temporal
Von Schacky [105] ^a^	Development and validation	Quaternary	Retrospective	Multi-institutional	Germany	Germany (1)	Temporal and geographical
Wang [98] ^ac^	Development and validation	Tertiary	Retrospective	Multi-institutional	China	China (1)	Temporal and geographical
Xiong [99] ^ad^	Development and validation	Tertiary	Retrospective	Multi-institutional	China	China (3)	Temporal and geographical
Xu [69] ^b^	Development and validation	Tertiary	Retrospective	Single institution	China	China (1)	Temporal and treatment stage
Yamada [63] ^a^	Development and validation	Secondary	Retrospective	Multi-institutional	Japan	Japan (7)	Geographical, temporal and scanner type
Yang [95] ^a^	Development and validation	Tertiary	Retrospective	1 public database, 1 institution	USA (SEER dataset)	China (1)	Geographical (international)
Yang [64] ^a^	Development and validation	Tertiary	Retrospective	Multi-institutional	South Korea	South Korea (1)	Temporal and geographical
Zachariah [106]^de^	Development and validation	Quaternary	Prospective	Single institution	USA	USA (1)	Temporal
Zhang [110] ^a^	Development and validation	Tertiary	Retrospective	Single institution	China	China (1)	Temporal
Zhang [60] ^bd^	Development and validation	Tertiary	Prospective	Single institution	China	China (1)	Temporal
Zhang [79] ^ac^	Development and validation	Unknown	Retrospective	Multi-institutional	China	China (1)	Temporal and geographical
Zhang [56] ^ac^	Development and validation	Not applicable	Retrospective	Multi-institutional database	USA (TCIA)	USA (TCIA) (1)	Geographical
Zhao [83] ^ac^	Development and validation	Tertiary	Retrospective	Single institution	USA	USA (1)	Temporal
Zhu [65] ^a^	Development and validation	Unknown	Internal: prospective; external: retrospective	Multi-institutional	China	China (1)	Temporal and geographical; design of data acquisition

If available, the number of institutions used for validation is presented in parentheses in column Validation Countries

*CT* Computed Tomography, *NHGRP* National Human Genetic Resources Sharing Service Platform, *NLST* The National Lung Screening Trial, *PLCO* Prostate, Lung, Colorectal and Ovarian (PLCO) Cancer Screening Trial, *SEER* Surveillance, Epidemiology, and End Results program, *TCGA* The Cancer Genome Atlas, *TCIA* The Cancer Imaging Archive, *UK* United Kingdom, *USA* United States of America

^a^Validation explicitly stated in abstract

^b^Validation indirectly mentioned in abstract

^c^Reports decision threshold

^d^Evaluated in a clinical setting

^e^Implemented in Practice

^f^Regression model

Overall, 74 medical datasets were used for external validation across the 56 studies, averaging 1.3 per paper (range = 1—8). All studies used real-world data acquired prospectively or collected from the patients' EHRs and imaging archiving platforms. Except for three articles using both standard and uncommon types of MRI scans [57, 60, 65] and one using endocytoscopy (whose use is still growing) [61], all studies used text- and image-based data routinely collected in clinical practice. However, only nine reports describe external validation in clinically realistic scenarios [57, 58, 60, 61, 71, 87, 91, 101, 106], and solely two systems are currently implemented in practice [61, 106]. The papers involved several cancer-related settings, including secondary (*n* = 1, 2%), tertiary (*n* = 34, 61%), and quaternary (12, 21%) oncology care. However, 6 (11%) studies did not report from which centers data were retrieved, and 3 (5%) used databases without this information.

Among the collected studies, 49 (87.5%) were conducted retrospectively, 3 (5.36%) were prospective, 4 (7.15%) were mixed: one performed internal validation prospectively and external validation retrospectively [65], one proceeded inversely [101], and two used both retrospective and prospective cohorts [71, 91]. Only one report used randomized data [94]. Regarding validation design, 31 (55.357%) studies followed a multi-institutional approach, 14 (25%) collected information from a single center, 1 (1.786%) only used public databases, 2 (3,572%) used public multi-institutional databases, and 8 (14,286%) used both types of sources. For the multi-institutional studies (including databases), the average number of facilities used for validation was 3, with a maximum of 33 [94]. One study did not report the number of institutions involved [73].

The following freely available data sources were used: (i) the Surveillance, Epidemiology, and End Results (SEER) database, which covers population-based cancer registries of approximately 47.8% of the United States Population [59, 72, 93, 95, 107]; (ii) The Cancer Genome Atlas (TCGA, from the USA), which molecularly characterizes over 20,000 primary cancers, and contains whole-slide images [75]; (iii) The Cancer Imaging Archive, which hosts a large number of medical images for various types of cancer [56]; (iv) the Edinburgh dataset, containing data from the University of Edinburgh (Scottland, United Kingdom) [87]; (v) the Prostate, Lung, Colorectal, and Ovarian (PLCO) randomized trial sponsored by the by the National Cancer Institute (NCI), designed to evaluate the impact of cancer screening on mortality rates, as well as to assess the potential risks and benefits associated with screening [94]; (vi) the National Lung Screening Trial (NLST), a randomized controlled trial also supported by the NCI that aimed to evaluate the impact of using low-dose helical CT scans on patient mortality [94]; (vii) the PROSTATEx dataset, which contains a retrospective set of prostate MRI studies [82]; (viii) the PICTURE dataset, containing data from a single-center trial, and intended to evaluate the diagnostic accuracy of multiparametric magnetic resonance imaging (mpMRI) in men with prostate lesions [82]; and (ix) the National Human Genetic Resources Sharing Service Platform (NHGRP), for which we could not find any details [67].

In two studies, models were trained using data from multiple countries. One developed their model using patients from three Chinese institutions and one center from the United States of America (USA) and validated it on a Chinese dataset (*n* = 1, 1.8%) [102]. The other gathered data from a Chinese institution and TCGA and validated their model on images from NHGRP [67]. Additionally, one document did not report which countries were involved in their model’s development or validation [73]. All other authors developed their model on data from a single country. These included China (*n* = 19, 33.7%), the USA (*n* = 12, 21.4%), South Korea (*n* = 9, 16.1%), Italy and Germany (3 each, 5.4%), Japan and the Netherlands (2 each, 3.6%), and the United Kingdom (UK), Canada, and Austria (1 each, 1.8%).

Besides the two abovementioned papers [67, 102], twelve other studies performed international validation. Of these, six included ethnically different sources. Two authors trained their model with data from South Korea: one validated it on South Korean and American datasets [90], and the other validated it on a South Korean dataset and the Edinburgh dataset (UK) [87]. Additionally, five reports mention training their model on the SEER database (USA), with four validating it with Chinese patients [72, 93, 95, 107] and one with South Korean patients [59]. For the five remaining studies, patients with the same ethnicity were included: (i) one was developed with the NLST trial dataset (USA) and validated on data from the UK [96]; (ii) one was trained with data from TCGA (USA) and validated on an institution from the UK [96]; (iii) one used data from Italy for training and patients from The Netherlands for validation [103]; (iii) one trained their model on the PROSTATEx dataset (from The Netherlands) and validated it on the PICTURE dataset (from the UK) [82]; and (iv) one used a Chinese dataset for training and Chinese and South Korean patients for validation [91].

Regarding validation types, 12 studies (21.48%) were limited to temporal validation from a single institution, which cannot be interpreted as a fully independent validation [57, 60, 74, 77, 78, 81, 83, 89, 106, 108–110]. Five other studies also only temporally validated their model. However, two used a multi-institutional approach (3.58%) [66, 70], two (3.58%) used different data acquisition designs (retrospective internal validation and prospective external validation) [71, 101], and one evaluated performance for patients at different treatment stages (1.78%) [69]. Nine studies (16,08%) only validated their model geographically, seven within the same country [56, 58, 62, 84, 92, 100, 115], one internationally [95], and one with internationally and ethnically different patients [102]. Twenty-nine reports (51.8%) included both temporal and geographical validation. Sixteen (28.57%) used local data, one evaluated temporally and geographically different patients from the same country with images captured using various scanners [96], and one (1.79%) used national data and mixed data acquisition (prospective internal validation and retrospective external validation) [65]. Lastly, one study that did not report data sources validated their model on different types of computed tomography (CT) scanners [73].

The external datasets were used to evaluate the models’ generalizability to populations differing – geographically, temporally, or both – from the development cohort. The performance metrics reported in the articles can be branched into three categories: discrimination, calibration, and processing time. For classification models, an average of 5 metrics were used to assess discrimination, up to a maximum of seven (range = 1 – 7). These consisted of (i) sensitivity, reported in 48 papers; (ii) area under the receiver operating characteristic (ROC) curve (AUC), calculated in 43 studies; (iii) specificity, used in 42 articles; (iv) accuracy, presented in 35 documents; (v and vi) positive and negative predictive values (PPV and NPV), computed in 29 and 19 reports, respectively; (vii) F1-score, considered in 13 papers; (viii) C-index, used in 2 articles [68, 95]; (ix) false positive rate, reported in two papers [82, 108]; (x) area under the alternative free-response ROC curve (AUAFROC) [108], calculated for one model; (xi) jackknife alternative free-response ROC (JAFROC), also computed for one algorithm [90]; and (xii) Softspot (Sos) and Sweetspot (Sws) flags, both used in the same two papers [71, 73]. However, decision thresholds were only disclosed for half of the articles (26/52, 50%), and only three papers presented results for different cut-off values/settings [82, 85, 87]. Likewise, 39 classification studies did not assess calibration. When evaluated (13/52, 25%), calibration was illustrated graphically in five studies (9.62%) [68, 79, 89, 94, 95], via Brier Score in three documents (5.77%) [91, 107, 115], using both approaches in four papers (7.69%) [86, 92, 103, 104], and with mean absolute error (MAE) in one report [82]. Lastly, the models’ processing time was also seldomly revealed, with only seven studies reporting it [62, 63, 66, 70, 80, 97, 101].

For the regression-based algorithms, discriminative performance was assessed via C-index [67, 68, 72, 74, 76, 93]. Regarding calibration, the model’s Brier Score was presented in one study [74], calibration plots in two [68, 72], both metrics in one [93], and none in two [67, 76]. The models’ processing time and decision thresholds were not reported in any of these studies.

#### Clinical utility

From the selected studies, the majority (*n* = 50, 89.29%) explicitly mentions the assessment of the models' clinical utility, that is, its relevance to clinicians and patient outcomes, in the paper's abstract. However, one only refers to it indirectly (1.79%) [68], and the remaining five (8.93%) do not state this aspect in their summaries [67, 69, 73, 90, 115]. Two approaches were used to assess the models’ utility: comparison against clinician performance, adopted in most studies (40/56, 71.4%), and benchmarking against established clinical tools (15/56, 26.8%). Additionally, one study used both: retrospective comparisons were performed against routine clinical scores, while prospective assessments involved clinicians (1/56, 1.8%) [91].

##### Comparison Against Clinicians

Four hundred-ninety-nine medical professionals of varying expertise were involved in these studies, with an average of 12 clinicians compared against each model (range = 1 – 109 [87]). These included endoscopists (*n* = 204), oncologists (*n* = 77), radiologists (*n* = 76), general physicians (*n* = 71), dermatologists (*n* = 44), pathologists (*n* = 21), ophthalmologists (*n* = 3), and thoracic surgeons (*n* = 3). A subset of 113 115 patients (102 178 female, 9 619 male) was used for these assessments, and identical performance metrics as those documented for external validation were observed, plus time until diagnosis. Specific clinicians’ years of experience were reported in 20 papers (48.8%), ranks (without years) in 11 (26.8%), and no information concerning expertise in 10 (24.4%). The 41 classification studies encompassing model comparison against clinicians can be divided into two designs: with and without the model and independent evaluation of the models and the clinicians.

The most commonly adopted technique was separately assessing model and clinician performance and comparing it posteriorly (*n* = 30, 73.2%). Four hundred-one clinicians (μ = 15 per report, range = 1 – 109) and 109 720^3^ patients (μ = 3 657 per paper, 100 965 female, 8 203 male^4^) were involved in these papers, and model-clinician performance was compared for detection and diagnostic capabilities. An average of 4 performance metrics (range = 1 – 7 [62]) were computed per paper, with sensitivity being the most calculated (*n* = 23), followed by specificity (*n* = 18) and accuracy (*n* = 15), AUC (*n* = 11), PPV (*n* = 11), NPV (*n* = 7), F1-score (*n* = 3) [57, 62, 83], false positive rate (*n* = 2) [82, 108], Sweetspot and Softsoft flags (*n* = 2) [71, 73], diagnostic time (*n* = 1) [62], and AUAFROC (*n* = 1) [108], and JAFROC (*n* = 1) [90].

The second approach involved comparing clinician performance with and without the assistance of the artificially intelligent systems developed by the authors (*n* = 11, 26.8%). The eleven studies employing this method comprised 92 clinicians (μ = 8, minimum = 1, maximum = 20 [70]) and 3 337 patients (μ = 370, 1 223 female, 1 416 male^5^). Similarly to the previous technique, an average of 4 performance metrics were used per paper (range = 1 [75, 90] – 6 [60, 101]), including sensitivity (*n* = 9), specificity (*n* = 8), accuracy (*n* = 8), PPV (*n* = 6), NPV (*n* = 5), AUC (*n* = 2) [68, 90], mean diagnostic time (*n* = 2) [70, 101], and error rate (*n* = 1) [60].

##### Comparison Against Standard/Established Clinical Tools

In sixteen studies, assessing the usefulness of the models involved comparing their performance against well-established and routinely used clinical tools. In total, 11 659 patients (μ = 777 per paper, 4 521 female, 5 694 male^6^) were encompassed in these assessments, and twelve standard tools were used for comparisons. These included: (i) the 7th and 8th editions of the Tumor, Node, and Metastasis (TNM) staging system; (ii) the Brock University Model; (iii) the Fracture Risk Assessment Tool (FRAX); (iv) the Liver Cancer Study Group of Japan (LCSGJ); (v) the Mayo clinic model; (vi) the modified Glasgow Prognostic Score (mGPS); (vii) the Osteoporosis Self-Assessment Tool for Asians (OSTA); (viii) the second version of the Prostate Imaging Reporting and Data System (PI-RADS v2); (ix) the Peking University (PKU) model; (x) the PLCOm2012 model; (iv) the Response Evaluation Criteria in Solid Tumors (RECIST); (xi) the Veterans Affairs (VA) model; and (xii) the World Health Organization (WHO) performance status. Except for one study [69], all papers explicitly mention comparisons against these tools in the abstract.

The TNM system, created by the American Joint Committee on Cancer (AJCC), is globally used in routine clinical procedures. It categorizes cancer progression and guides subsequent treatment decisions depending on (i) the size and extent of the primary tumor (T), (ii) if it has spread to nearby lymph nodes (N), and (iii) if it has metastasized to distant organs (M) [121]. In this review, two text-based classification studies compared their models against the 7th edition of this staging system (TNM-7): one juxtaposed diagnostic and prognostic (3-year overall survival) predictions for bone metastasis in kidney cancer patients (323 women, 640 men) [107], while the other compared 1–10-year postoperative survival predictions for patients with colorectal cancer (607 women, 965 men) [59]. Similarly, seven papers resorted to the 8th edition of AJCC TMN (TNM-8), its revised and updated version. On the one hand, in four articles, the models were only compared against this system. Two analyzed their text- and regression-based models to predict cancer-specific survival for esophageal (500 patients, 150 women, 350 men) [72] and lung tumors (1 182 individuals, 642 female, 540 male) [93]. The other two concerned the evaluation of classification models. Using preoperative images and descriptive data, one compared 2-year overall survival and 1-year recurrence-free survival predictions for patients with pancreatic cancer (27 female, 26 male) [77]. The other compared risk stratification performance for overall survival for lung cancer patients (39 women, 133 men) between their model and the TMN-8 system using only text-based data [95].

On the other hand, in three text-based studies, models were compared against TNM-8 and other tools. One paper also contrasted model performance for recurrence, recurrence-free survival, and overall survival for lung cancer patients (71 women, 88 men) [92] with the WHO performance status, often used in oncology to determine patients' overall health status, prognosis, and the ability to tolerate treatment [122]. This scaling system ranges from 0 to 4, where 0 represents no symptoms and pre-disease performance, and 4 translates to total disability. In the second article, predictions of overall postoperative survival were benchmarked against TNM-8 and LCSGJ (42 liver cancer patients, 12 women, 30 men) [74]. LCSGJ is a group of Japanese medical professionals specializing in diagnosing and treating liver cancer, recognized as a leading authority in this cancer research field. Lastly, the third study describes the development of three risk models for breast cancer patients (150 women) [84]: (i) fracture, whose predictions were contrasted with those generated by FRAX; (ii) osteoporosis, compared against and FRAX and OSTA; (iii) and survival, benchmarked against TNM-8. FRAX is a web-based tool designed to stratify 10-year bone fracture risk, and OSTA assesses the risk of osteoporosis in Asian populations [84].

The Brock University (also known as PanCan) model is a logistic regression model devised to assist in risk stratification for lung cancer. It is recommended in the British Thoracic Society guideline as a tool to decide if nodules measuring 8 mm or more in maximum diameter should be assessed further with PET-CT [96]. Here, it was applied in one of the selected papers to compare predictions of malignancy risk for lung cancer from CECT and NECT scans (1 397 images, 1187 patients, unknown gender proportion) [96]. In addition to the Brock Model, comparisons in a second paper (978 CTs, 493 patients, 297 women, 196 men) were also performed against three other tools: (i) the Mayo model, which the Mayo Clinic developed to assess cancer prognosis and predict patient outcomes; (ii) the PKU model, created by the Peking University; and (iii) the VA model, which includes a comprehensive cancer care system that aims to provide high-quality, evidence-based care to veterans with cancer [91].

The mGPS scale is a validated scoring system formulated to assess the prognosis of patients with advanced or metastatic cancer based on nutritional and inflammatory markers [76]. In this review, it was used to establish clinical utility for a text-based classification model developed to predict overall survival for patients with unresectable pancreatic tumors (22 patients, 8 women, 14 men) [76].

PI-RADS is a standardized system for interpreting and reporting findings from prostate MRI scans, created to guide clinical decision-making in diagnosing and treating prostate cancer. In this context, it was contrasted against a model developed to stratify low- and high-risk patients (39 and 14 men, respectively) [81].

PLCOm2012 is a validated risk score that uses logistic regression to predict the probability of lung cancer occurrence within six years based on demographic and clinical information [123]. It was the chosen comparator in a study predicting 12-year lung cancer incidence using low-dose CT images and patients’ age, sex, and smoking status (5493 images and patients, 2456 women, 3037 men) [94].

Finally, RECIST is a set of guidelines used to evaluate the response of solid tumors to treatment in clinical trials and clinical practice. It was compared against two classification models: one aimed at detecting pathological downstaging in advanced gastric cancer patients from CECT images (86 patients and images, 23 women, 27 men) [69]; the other was designed to predict pathological tumor regression grade response to neoadjuvant chemotherapy in patients with colorectal liver metastases from MRI scans (61 images, 25 patients, 13 female, 12 male) [65].

A few performance metrics were reported for the comparisons between the models developed in the selected papers and routinely used clinical tools, with an average of 3 metrics reported per document (range = 1 – 6). Here, the most frequently calculated metrics were AUC (*n* = 11) and sensitivity (*n* = 8), but PPV (*n* = 5), C-index (*n* = 4), specificity (*n* = 4), accuracy (*n* = 3), NPV (*n* = 3), Brier Score (*n* = 2) and F1-score (*n* = 1) were also used in the evaluations.

### Primary tumors

Fifty-one papers (91.1%) describe models developed for primary tumor-related assessments. These include cancers of the CNS (brain [56–58]), digestive (colorectal [59–65], esophageal [71–73], gastric [66–70], and hepatic [74, 75] malignancies), endocrine (pancreas [76, 77] and thymus [78]), genitourinary (bladder [79], cervix [80], prostate [81, 82], and uterus [83, 115]), and integumentary (breast [84–86] and skin [87, 88]) systems, respiratory system and associated tissues (larynx [99], lung [89–98], mesothelium [100], and nasopharynx [101]), and the skeleton (cartilages and bones [102–105]).

#### Central nervous system

Three retrospective studies were developed to diagnose brain cancers using MRI scans, amounting to 1 084 patients and 64 459 images, resulting in an average sensitivity of 81.97% and specificity of 91.63 (Table 7) [56–58]. The first involved the following conditions: acoustic neuroma, pituitary tumor, epidermoid cyst, meningioma, paraganglioma, craniopharyngioma, glioma, hemangioblastoma, metastatic tumor, germ cell tumor, medulloblastoma, chordoma, lymphomas, choroid plexus, papilloma, gangliocytoma, dysembryoplastic neuroepithelial tumor, and hemangiopericytoma [58]. The CNN-based model was trained on images from 37 871 patients and externally validated using 64 414 T1-weighted, T2-weighted, and T1c MRI scans from 1039 subjects (600 female, 349 male) from three institutions. Its diagnostic performance was compared against nine neuroradiologists (5 to 20 years of experience) to assess clinical utility. This CNN classified brain tumors with high accuracy, sensitivity, and specificity, performing particularly well in identifying gliomas, which are difficult to diagnose using traditional imaging methods. When aided by the model, the neuroradiologists' accuracy increased by 18.9%, which was still lower than the model alone. AI assistance also boosted the neuroradiologists' sensitivity, specificity, and PPV. However, only three types of scans were considered, training data was obtained from a single center, and few rare tumors were included.

**Table 7:** External validation and clinical utility performance for cancers of the central nervous system (brain tumor diagnosis from MRI scans)

Outcome	Author	Patients	Images	Comparator	AUC	ACC	SEN	SPEC	PPV	F1-Score
Detection and diagnosis (multiclass, 18 recognized conditions)	Gao [58]	1039 (600♀, 439♂)	64,414 T1-weighted, T2-weighted, T1c MRI scans	**Model (CNN, MRI-based)**	NR	**76.33333**	**77.933**	**91.933**	**74.8**	**0.68333**
				Neuroradiologists, *n* = 9, 5 to 20 years of experience)—Unaided	NR	63.5	63.8	95.3	NR	NR
				**Neuroradiologists,** ***n*** **= 9, 5 to 20 years of experience)—Aided**	NR	**75.5**	**81.4**	**97.1**	NR	NR
Diagnosis (5-way, glioblastoma vs. anaplastic glioma vs. meningioma vs. primary central nervous system lymphoma vs. brain metastasis)	Stadlbauer [57]	20 (11♀, 9♂)	20 cMRI advMRI, and physMRI scans	Model 1 (AdaBoost, MRI-based)	0.862	**87.5**	75	90.2	**82.7**	**0.774**
				Model 2 (RF, MRI-based)	0.886	83	75	84.4	76.5	0.752
				Radiologists (*n* = 2)	0.813	85	**76.7**	**92.5**	79.8	0.74
diagnosis (binary, low-grade glioma vs. glioblastoma)	Zhang [56]	25 (1♀, 13♂)	25 T1-weighted and T2-weighted MRI scans	**Model (RF, MRI-based)**	**1**	NR	**100**	**100**	NR	NR
				Neuroradiologist 1 (15 years of experience)	0.92	NR	NR	NR	NR	NR
				Neuroradiologist 2 (1 year of experience)	0.7	NR	NR	NR	NR	NR
				Radiologist (3 years of experience)	0.59	NR	NR	NR	NR	NR

*AdaBoost* Adaptive Boosting, *ACC* Accuracy, *AUC* Area Under the ROC Curve, *CNN* Convolutional Neural Network, *MRI* Magnetic Resonance Imaging, *NPV* Negative Predictive Value, *NR* Not reported, *PPV* Positive Predicted Value, *RF* Random Forest, *ROC* Receiver Operating Characteristic, *SEN* Sensitivity, *SPEC* Specificity

In the second paper, the authors explored the combination of 9 different ML models – NB, logistic regression, SVM with a polynomial kernel, kNN (k = 3), DT, MLP, RF, AdaBoost, and bootstrap aggregating – to distinguish between different types of brain tumors (glioblastoma, anaplastic glioma, meningioma, primary central nervous system lymphoma, and brain metastasis) [57]. MRI techniques were analyzed in a combination of 135 classifiers and radiomics: cMRI, advMRI, phyMRI, cMRI + phyMRI, and advMRI + phyMRI. A dataset of 167 patients was used for training, and temporal validation was performed on 20 subjects. Physiological MRI scans (phyMRI), named radiophysiomics, achieved the best results using AdaBoost with cMRI and phyMRI and RF with phyMRI. Both models surpassed the radiologists in AUC and F1-score but were outperformed in sensitivity and specificity. The AdaBoost model also had a higher PPV than the clinicians. However, this was a single-center, retrospective study, and the application and tuning of the models were performed manually.

The third study evaluated the usefulness of preoperative contrast-enhanced T1- and T2-weighted MRI in differentiating low-grade gliomas (LGG) from glioblastomas (GBM) [56]. The authors trained a radiomics-based RF classifier on 142 patients from 8 American centers and externally validated it on 25 patients from another institution (all from The Cancer Imaging Archive). The results showed that the machine learning algorithm was highly accurate in differentiating between GBM and LGG based on preoperative contrast-enhanced MRI scans, surpassing two neuroradiologists (15 and 1 year of experience) and a radiologist (3 years of experience). However, few patients from a public database were collected, possibly resulting in selection bias (non-random selection).

#### Digestive system

Malignancies of the digestive system – highlighted in Table 8 – were the most comprehensively studied (17/56, 30.4%), encompassing colorectal (*n* = 7, 41.2%), esophageal (*n* = 3, 17.6%), gastric (*n* = 5, 29.4%), and liver (*n* = 2, 11.8%) cancers.

**Table 8:** External validation and clinical utility performance for primary digestive cancer assessments

Cancer	Author	Patients	Images	Outcome	Comparator	AUC	ACC	SEN	SPEC	PPV	NPV	F1-Score	FPR	MDT per image (s)	Sos flag	Sws flag	C-index	Brier Score
**Colorectal**	Choi [62]	NR	400 white-light colonoscopy images	Diagnosis (4-way, healthy vs. A-LGD vs. A-HGD vs. adenocarcinoma)	**Model (CNN, image-based)**	**0.92**	**NR**	**77.25**	**92.42**	**77.16**	**92.58**	**0.7681**	NR	**0.12 s**	**NR**	**NR**	**NR**	**NR**
					Expert Endoscopists (*n* = 4, 5 years of experience)	NR	NR	72.38	90.58	71.38	90.89	0.7187	NR	7.72 s	NR	NR	NR	NR
					Trainees Endoscopists (*n* = 6, < 2 years)	NR	NR	62.5	86.47	61.91	87.07	0.6217	NR	8.13 s	NR	NR	NR	NR
	Kudo [61]	89 (26♀, 63♂)	100 endocytoscopic images	Diagnosis (binary, neoplastic vs. non-neoplastic)	Model (SVM, stained endocytoscopic images)	NR	**98**	**96.9**	**100**	**100**	**94.6**	NR	NR	NR	NR	NR	NR	NR
					Trainee Endoscopists (*n* = 20, stained endocytoscopic images)	NR	69	70.8	65.7	79.3	54.8	NR	NR	NR	NR	NR	NR	NR
					Expert Endoscopists (*n* = 10, stained endocytoscopic images)	NR	93.3	92.8	94.3	96.8	87.5	NR	NR	NR	NR	NR	NR	NR
					Model (SVM, narrow-band endocytoscopic images)	NR	96	**96.9**	94.3	96.9	**94.3**	NR	NR	NR	NR	NR	NR	NR
					Trainee Endoscopists (*n* = 20, narrow-band endocytoscopic images)	NR	70.4	70.4	85.7	89	54.9	NR	NR	NR	NR	NR	NR	NR
					Expert Endoscopists (*n* = 10, narrow-band endocytoscopic images)	NR	94.6	93.5	**96.6**	**98.1**	89	NR	NR	NR	NR	NR	NR	NR
	Osman [59]	1572 (607♀, 965♂)	NA	Overall survival (1y)	**Model (LightGBM, text-based)**	**0.8255**	**80.08**	NR	NR	NR	NR	NR	NR	NR	NR	NR	NR	NR
					TNM-7	0.7513	NR	NR	NR	NR	NR	NR	NR	NR	NR	NR	NR	NR
				Overall survival (2y)	**Model (LightGBM, text-based)**	**0.8362**	78.16	NR	NR	NR	NR	NR	NR	NR	NR	NR	NR	NR
					TNM-7	0.7666	NR	NR	NR	NR	NR	NR	NR	NR	NR	NR	NR	NR
				Overall survival (3y)	**Model (LightGBM, text-based)**	**0.8102**	**77.69**	NR	NR	NR	NR	NR	NR	NR	NR	NR	NR	NR
					TNM-7	0.7514	NR	NR	NR	NR	NR	NR	NR	NR	NR	NR	NR	NR
				Overall survival (4y)	**Model (LightGBM, text-based)**	**0.8052**	**76.41**	NR	NR	NR	NR	NR	NR	NR	NR	NR	NR	NR
					TNM-7	0.7515	NR	NR	NR	NR	NR	NR	NR	NR	NR	NR	NR	NR
				Overall survival (5y)	**Model (LightGBM, text-based)**	**0.8046**	**75.2**	NR	NR	NR	NR	NR	NR	NR	NR	NR	NR	NR
					TNM-7	0.7367	NR	NR	NR	NR	NR	NR	NR	NR	NR	NR	NR	NR
				Overall survival (6y)	**Model (LightGBM, text-based)**	**0.7875**	**74.57**	NR	NR	NR	NR	NR	NR	NR	NR	NR	NR	NR
					TNM-7	0.7246	NR	NR	NR	NR	NR	NR	NR	NR	NR	NR	NR	NR
				Overall survival (8y)	**Model (LightGBM, text-based)**	**0.7876**	**73.67**	NR	NR	NR	NR	NR	NR	NR	NR	NR	NR	NR
					TNM-7	0.7154	NR	NR	NR	NR	NR	NR	NR	NR	NR	NR	NR	NR
				Overall survival (10y)	**Model (LightGBM, text-based)**	**0.7772**	**74.21**	NR	NR	NR	NR	NR	NR	NR	NR	NR	NR	NR
					TNM-7	0.7028	NR	NR	NR	NR	NR	NR	NR	NR	NR	NR	NR	NR
	Yamada [63]	NR	255 narrow-band, and blue-laser colonoscopy images, 128 lesions	Diagnosis (binary, neoplastic vs. non-neoplastic)	**Model (CNN, image-based)**	**0.903**	**89**	**88.3**	**90.3**	**NR**	**NR**	**NR**	NR	**0.0129 s**	**NR**	**NR**	**NR**	**NR**
					Novice Endoscopists (*n* = 4)	NR	NR	82.5	66.1	NR	NR	NR	NR	NR	NR	NR	NR	NR
					Fellow Endoscopists (*n* = 3)	NR	NR	85	71	NR	NR	NR	NR	NR	NR	NR	NR	NR
					Expert Endoscopists (*n* = 4)	NR	NR	87.9	72.6	NR	NR	NR	NR	NR	NR	NR	NR	NR
	Yang [64]	240 (79♀, 161♂)	240 white-light colonoscopy images	Diagnosis—7-way classification	**Model (CNN, Inception-ResNet-v2, image-based)**	NR	74.3	**NR**	NR	NR	NR	NR	NR	NR	NR	NR	NR	NR
					Model (CNN, ResNet-152, image-based)	NR	74.7	NR	NR	NR	NR	NR	NR	NR	NR	NR	NR	NR
				Diagnosis—4-way classification	Model (CNN, ResNet-152, image-based)	NR	79.2	NR	NR	NR	NR	NR	NR	NR	NR	NR	NR	NR
					Model (CNN, Inception-ResNet-v2, image-based)	NR	76	NR	NR	NR	NR	NR	NR	NR	NR	NR	NR	NR
					**Best-Performing Endoscopist**	NR	**85.5**	NR	NR	NR	NR	NR	NR	NR	NR	NR	NR	NR
					Worst-Performing Endoscopist	NR	68	NR	NR	NR	NR	NR	NR	NR	NR	NR	NR	NR
				Diagnosis—neoplastic vs. non-neoplastic	Endoscopist 1	0.691	67.5	41.7	**96.5**	**93**	59.6	NR	NR	NR	NR	NR	NR	NR
					Endoscopist 2	**0.853**	**85**	79.5	91.2	91	79.8	NR	NR	NR	NR	NR	NR	NR
					Endoscopist 3	0.791	79.6	80.3	78.8	81	78.1	NR	NR	NR	NR	NR	NR	NR
					ResNet-152	0.818	73.3	**91.9**	52.5	68.8	**85.8**	NR	NR	NR	NR	NR	NR	NR
					Inception-ResNet-v2	0.76	71.5	**92.4**	44.2	65.4	**83.6**	NR	NR	NR	NR	NR	NR	NR
				Diagnosis—advanced vs. non-advanced	Endoscopist 1	0.723	96.7	45.5	99.1	71.4	97.4	NR	NR	NR	NR	NR	NR	NR
					Endoscopist 2	**0.95**	**98.8**	**90.9**	**99.1**	**83.3**	**99.6**	NR	NR	NR	NR	NR	NR	NR
					Endoscopist 3	0.883	94.2	81.8	94.8	42.9	99.1	NR	NR	NR	NR	NR	NR	NR
					ResNet-152	0.829	93.6	48.5	95.8	36.7	97.5	NR	NR	NR	NR	NR	NR	NR
					Inception-ResNet-v2	0.876	94.6	51.5	96.7	43	97.6	NR	NR	NR	NR	NR	NR	NR
	Zhang [60]	93 (41♀, 52♂)	93 diffusion kurtosis and T2-weighted MRI scans	pCR	Model (CNN, image-based)	0.99	**97.8**	**100**	**97.3**	**90**	**100**	NR	**2.2**	NR	NR	NR	NR	NR
					Radiologist 1 (10 years of experience)—Unaided	NR	73.1	55.6	77.3	37.1	87.9	NR	26.9	NR	NR	NR	NR	NR
					Radiologist 1 (10 years of experience)—Aided	NR	**87.1**	**72.2**	**90.7**	**65**	**93.2**	NR	**12.9**	NR	NR	NR	NR	NR
					Radiologist 2 (15 years of experience)—Unaided	NR	75.2	66.7	77.3	41.4	90.6	NR	24.8	NR	NR	NR	NR	NR
					Radiologist 2 (15 years of experience)—Aaided	NR	**86**	**77.8**	**88**	**60.9**	**94.3**	NR	**14**	NR	NR	NR	NR	NR
				T downstaging	Model (CNN, image-based)	0.79	**75.3**	**77.8**	**74.7**	42.4	**93.3**	NR	**22.6**	NR	NR	NR	NR	NR
					Radiologist 1 (10 years of experience)—Unaided	NR	72	81.6	61.4	70.2	75	NR	38	NR	NR	NR	NR	NR
					Radiologist 1 (10 years of experience)—Aided	NR	**73.1**	**83.7**	**61.4**	**70.7**	**77.1**	NR	**26.9**	NR	NR	NR	NR	NR
					Radiologist 2 (15 years of experience)—Unaided	NR	71	79.6	61.4	69.6	76.7	NR	39	NR	NR	NR	NR	NR
					Radiologist 2 (15 years of experience)—Aaided	NR	**73.1**	**81.6**	**63.6**	**71.4**	75.7	NR	**26.9**	NR	NR	NR	NR	NR
				TRG	Model (CNN, image-based)	0.7	**75.3**	60.8	**78.6**	40.7	**89.4**	NR	**31.2**	NR	NR	NR	NR	NR
					Radiologist 1 (10 years of experience)—Unaided	NR	**62.4**	62.7	**61.9**	66.7	**57.8**	NR	**37.6**	NR	NR	NR	NR	NR
					Radiologist 1 (10 years of experience)—Aided	NR	57	**68.8**	54.8	61.2	52.3	NR	43	NR	NR	NR	NR	NR
					Radiologist 2 (15 years of experience)—Unaided	NR	**65.6**	64.7	**69**	**71.7**	**61.7**	NR	34.4	NR	NR	NR	NR	NR
					Radiologist 2 (15 years of experience)—Aaided	NR	63.4	**64.7**	61.9	61.2	59.1	NR	**26.6**	NR	NR	NR	NR	NR
	Zhu [65]	25 (13♀, 12♂)	61 T2-weighted- and DW-MRI scans	TRG	**Model (CNN, image-based)**	**0.833**	**88.5**	**91.8**	**75**	**93.8**	**69.2**	NR	NR	NR	NR	NR	NR	NR
					RECIST	0.558	53.3	50	61.5	82.8	25	NR	NR	NR	NR	NR	NR	NR
**Esophagus**	De Groof [71]	80	80 white-light endoscopic images (prospective comparison)	Detection	**Model (CNN, endoscopic images)**	**NR**	**87.5**	**92.5**	**82.5**	**NR**	**NR**	**0.881**	**NR**	**NR**	100/100^1^	97.3/91.9^1^	NR	NR
					Senior endoscopists (*n* = 17)	NR	74.8	76.5	73.1	NR	NR	NR	NR	NR	98.9/98.6^1^	98.6/97.8^1^	NR	NR
					Junior endoscopists (*n* = 8)	NR	77.7	79.1	76.3	NR	NR	NR	NR	NR	99.7/99.3^1^	99.3/97.8^1^	NR	NR
					Fellow endoscopists (*n* = 18)	NR	73.3	70.6	76.1	NR	NR	NR	NR	NR	97.9/97.4^1^	96.3/93.9^1^	NR	NR
					Novice endoscopists (*n* = 10)	NR	65.9	60.3	71.5	NR	NR	NR	NR	NR	96.6/95.3^1^	90.0/86.4^1^	NR	NR
		80	80 white-light endoscopic images		**Model (CNN, endoscopic images)**	NR	88.8	90	87.5	NR	NR	0.887	NR	NR	100/100^1^	100/97.2^1^	NR	NR
	Huang [72]	500 (150♀, 350♂)	NA	Cancer-specific survival	**Model (MLP, text-based, regression model)**	**NR**	NR	NR	NR	NR	NR	NR	NR	NR	NR	NR	**0.687**	**NR**
					TNM-8	NR	NR	NR	NR	NR	NR	NR	NR	NR	NR	NR	0.643	NR
	Van der Putten [73]	80	80 white-light endoscopic images	Detection and Location	**Model (CNN, endoscopic images)—clinically realistic lesions**	**NR**	**88.8**	**90**	**87.5**	**NR**	**NR**	NR	**NR**	**NR**	**100**	**97.2**	NR	NR
		80	80 white-light endoscopic images		**Model (CNN, endoscopic images)—visually subtle lesions**	**NR**	**87.5**	**92.5**	**82.5**	**NR**	**NR**	NR	**NR**	**NR**	**97.3**	**91.9**	NR	NR
					Senior endoscopists (*n* = 17, > 5Y)—visually subtle lesions	NR	74.8	76.5	73.1	NR	NR	NR	NR	NR	98.6	97.8	NR	NR
					Junior endoscopists (*n* = 8, < 3Y)—visually subtle lesions	NR	77.7	79.1	76.3	NR	NR	NR	NR	NR	99.3	97.8	NR	NR
					Fellow endoscopists (*n* = 18, trainees)—visually subtle lesions	NR	73.3	70.6	76.1	NR	NR	NR	NR	NR	96.3	93.9	NR	NR
					Novices (*n* = 10, no endoscopic expertise)—visually subtle lesions	NR	65.9	60.3	71.5	NR	NR	NR	NR	NR	90	86.4	NR	NR
		20	20 white-light colonoscopy images		Model (CNN, endoscopic images)—live setting	NR	**90**	**90**	**90**	**NR**	**NR**	NR	**NR**	**NR**	NR	NR	NR	NR
**Gastric**	Huang [67]	91 (60♀, 31♂)	175 WSIs	Overall Survival	Model (MLP, WSIs and clinical data)	NR	NR	NR	NR	NR	NR	NR	NR	NR	NR	NR	0.657	NR
				Diagnosis (binary, malignant vs. healthy)	Model (CNN + RNN, WSIs images)	NR	92	93.4	90.5	NR	NR	NR	NR	NR	NR	NR	NR	NR
					Junior Pathologist (*n* = 1)	NR	87.4	75.8	100	NR	NR	NR	NR	NR	NR	NR	NR	NR
					**Expert Pathologists (*n* = 3)**	**NR**	**97.9**	**96.7**	**95.2**	**NR**	**NR**	**NR**	**NR**	NR	NR	NR	**NR**	**NR**
	Jiang [68]	1043 (329♀, 714♂)	1043 CECTs	Peritoneal recurrence	**Model (contrastive learning + CNN)**	**0.843**	NR	**98.89**	**52.13**	**41.9**	**99.3**	**NR**	NR	NR	NR	NR	**0.778**	**NR**
					Physician 1 (11 years of experience)—Unaided	0.692	NR	61.5	NR	NR	NR	NR	NR	NR	NR	NR	NR	NR
					Physician 1 (11 years of experience)—Aided	0.814	NR	94.4	NR	NR	NR	NR	NR	NR	NR	NR	NR	NR
					Physician 2 (5 years of experience)—Unaided	0.726	NR	NR	NR	NR	NR	NR	NR	NR	NR	NR	NR	NR
					Physician 2 (5 years of experience)—Aided	0.816	NR	NR	NR	NR	NR	NR	NR	NR	NR	NR	NR	NR
					Physician 31 (21 years of experience)—Unaided	0.734	NR	NR	NR	NR	NR	NR	NR	NR	NR	NR	NR	NR
					Physician 3 (21 years of experience)—Aided	0.816	NR	NR	NR	NR	NR	NR	NR	NR	NR	NR	NR	NR
				Recurrence-free survival	Model (contrastive learning + CNN)	NR	NR	NR	NR	NR	NR	NR	NR	NR	NR	NR	0.61	0.61
	Kim [66]	69 (38♀, 31♂)	212 EUS images	Diagnosis (binary, GIST vs. non-GIST)	**Model (CNN, image-based)**	0.834	**79.2**	**83**	**75.5**	**77.2**	**81.6**	**NR**	NR	26 images per second	NR	NR	NR	NR
					Junior Endoscopists (*n* = 3, < 200 EUS examinations)	NR	72.8	80.8	64.8	70.2	77.5	NR	NR	NR	NR	NR	NR	NR
					Expert Endoscopists (*n* = 3, > 500 EUS examinations)	NR	76.4	76.8	62.3	66.9	72.5	NR	NR	NR	NR	NR	NR	NR
				Diagnosis (triple-way, GISTs vs. leiomyomas vs. schwannomas)	**Model (double CNN, image-based)**	NR	**75.5**	NR	NR	NR	NR	NR	NR	NR	NR	NR	NR	NR
					Junior Endoscopists (*n* = 3, < 200 EUS examinations)	NR	67.1	NR	NR	NR	NR	NR	NR	NR	NR	NR	NR	NR
					Expert Endoscopists (*n* = 3, > 500 EUS examinations)	NR	64.5	NR	NR	NR	NR	NR	NR	NR	NR	NR	NR	NR
	Tang [70]	54 (38♀, 16♂)	54 endoscopic videos	Diagnosis (binary, mucosal vs. submucosal)	**Model (CNN, video-based)**	**NR**	**94.4**	**93.2**	**100**	NR	NR	NR	NR	**0.15**	**NR**	**NR**	**NR**	**NR**
					Expert Endoscopists (*n* = 6)—Unaided	NR	86.7	85.2	93.3	NR	NR	NR	NR	**2.62**	**NR**	**NR**	NR	NR
					**Expert Endoscopists (*n* = 6)—Aided**	**NR**	**93.2**	**92.4**	**96.7**	NR	NR	NR	NR	2.76	NR	NR	NR	NR
					Novice Endoscopists (*n* = 14)—Unaided	NR	70.4	67.7	82.1	NR	NR	NR	NR	5.09	NR	NR	NR	NR
					**Novice Endoscopists (*n* = 14)—Aided**	**NR**	**84.6**	**88.6**	**92.1**	**NR**	**NR**	**NR**	**NR**	**3.12**	**NR**	**NR**	**NR**	**NR**
	Xu [69]	40 (13♀, 27♂)	40 CECTs	Detection—temporal cohort	**Model (SVC, image-based)**	**0.961**	**90**	**73.3**	**100**	NR	NR	NR	NR	NR	NR	NR	NR	NR
					RECIST (temporal cohort)	0.75	77.5	62.5	87.5	NR	NR	NR	NR	NR	NR	NR	NR	NR
				Treatment response prediction—temporal cohort	Model (SVC, image-based)	0.75	76.9	46.7	95.8	NR	NR	NR	NR	NR	NR	NR	NR	NR
		46 (10♀, 36♂)	46 CECTs	Detection—treatment stage cohort	**Model (SVC, image-based)**	**0.85**	**86**	**85.7**	86.2	NR	NR	NR	NR	NR	NR	NR	NR	NR
					RECIST (treatment stage cohort)	0.617	73.9	26.6	**96.8**	NR	NR	NR	NR	NR	NR	NR	NR	NR
				Treatment response prediction—treatment stage cohort	Model (SVC, image-based)	0.889	83.7	57.1	96.6	NR	NR	NR	NR	NR	NR	NR	NR	NR
**Liver**	Kiani [75]	80 (24♀, 56♂)	80 WSIs magnified 40 times	Diagnosis (binary, hepatocellular carcinoma vs. cholangiocarcinoma)	**Model (CNN, image-based)**	**NR**	**84.2**	**NR**	**NR**	**NR**	**NR**	**NR**	**NR**	**NR**	**NR**	**NR**	NR	NR
					GI pathologists (*n* = 3)—Unaided	NR	94.6	NR	NR	NR	NR	NR	NR	NR	NR	NR	NR	NR
					**GI pathologists (*n* = 3)—Aided**	**NR**	**96.3**	**NR**	**NR**	**NR**	**NR**	**NR**	**NR**	**NR**	**NR**	**NR**	NR	NR
					Non-GI subspecialty pathologists (*n* = 3)—Unaided	NR	84.2	NR	NR	NR	NR	NR	NR	NR	NR	NR	NR	NR
					**Non-GI subspecialty pathologists (*n* = 3)—Aided**	**NR**	**87.1**	**NR**	**NR**	**NR**	**NR**	**NR**	**NR**	**NR**	**NR**	**NR**	NR	NR
					Pathology trainees (*n* = 3)—Unaided	NR	85.8	NR	NR	NR	NR	NR	NR	NR	NR	NR	NR	NR
					**Pathology trainees (*n* = 3)—Aided**	**NR**	**89.6**	**NR**	**NR**	**NR**	**NR**	**NR**	**NR**	**NR**	**NR**	**NR**	NR	NR
					**Other pathologists (*n* = 2)—Unaided**	**NR**	**96.9**	**NR**	**NR**	**NR**	**NR**	**NR**	**NR**	**NR**	**NR**	**NR**	NR	NR
					Other pathologists (*n* = 2)—Aided	NR	93.1	NR	NR	NR	NR	NR	NR	NR	NR	NR	NR	NR
	Li [74]	42 (12♀, 30♂)	NA	Overall Survival (3y)	**Model (XGBoost + RF + GBDT, text-based)**	**NR**	**NR**	**NR**	**NR**	**NR**	**NR**	**NR**	**NR**	**NR**	**NR**	**NR**	**0.671**	**0.169**
					TNM-8	NR	NR	NR	NR	NR	NR	NR	NR	NR	NR	NR	0.648	0.198
					LCSGJ	NR	NR	NR	NR	NR	NR	NR	NR	NR	NR	NR	0.539	0.189

*A-HGD* Adenoma with High-Grade Dysplasia, *A-LGD* Adenoma with Low-Grade Dysplasia, *ACC* Accuracy, *AUC* Area Under the ROC Curve, *CECT* Contrast-Enhanced CT, *CNN* Convolutional Neural Network, *CT* Computed Tomography, *EUS* Endoscopic Ultrasonography, *FPR* False Positive Rate, *GBDT* Gradient-Boosted Decision Tree, *GI* Gastrointestinal, *LCSGJ* Liver Cancer Study Group of Japan, *LightGBM* Light Gradient-Boosting Machine, *MDT* Mean Diagnostic Time, *MLP* Multi-Layer Perceptron, *MRI* Magnetic Resonance Imaging, *NA* Not applicable, *NPV* Negative Predictive Value, *NR* Not Reported, *pCR* Pathological Complete Response, *RECIST* Response Evaluation Criteria in Solid Tumors, *ROC* Receiver Operating Characteristic, *SEN* Sensitivity, *Sos* Softspot, *SPEC* Specificity, *SVC* Support Vector Classifier, *SVM* Support Vector Machine, *Sws* Sweetspot, *TNM* Tumor, Node, and Metastasis staging system, *TNM-7* Seventh edition of the TNM staging system, *TNM-8* Eighth edition of the TNM staging system, *TRG* Tumor Regression Grade, *WSI* Whole-Slide Image, *Y* Years

##### Colorectal Cancer

Three sets of articles addressed colorectal cancers (7 papers). The goal of the first set, consisting of four multi-institutional retrospective studies, was its diagnosis, averaging a sensitivity of 77.3% and a specificity of 93.2% for tests on 995 images from different sources [61–64]. The authors in [62] developed an ensemble of three CNNs (Inception-v3, ResNet-50, and DenseNet-161) to predict the histology of colorectal neoplasms based on white light colonoscopic images. The ensemble model transferred knowledge from digital photography and learned with colonoscopic images to classify the images into one of 4 different pathologic categories: normal (healthy), adenoma with low-grade dysplasia (A-LGD), adenoma with high-grade dysplasia (A-HGD), and adenocarcinoma. The system's diagnostic performance was compared against four experts (more than five years of experience) and six trainees (less than two years). In the external validation dataset (400 images, 100 of each type), the CNN-CAD model achieved high accuracy in predicting the histology of the lesions. Compared to endoscopists, the model's performance was slightly better than the experts' and significantly outperformed the trainees. In addition, the authors used Grad-CAM to create a heatmap highlighting the regions of the input image that were most relevant to the network's decision. However, only one image per polyp was used; consequently, tumors that cannot be contained within a single image were neglected.

The second work [61] concerns the external validation and clinical utility assessment of EndoBRAIN, an AI-assisted system to classify colorectal polyps into malignant or non-malignant. EndoBRAIN was trained with 69 142 endocytoscopic images from patients with colorectal polyps from five academic centers in Japan. Its clinical validity had previously been confirmed in a single-center prospective study. However, since its implementation depends on governmental regulatory approval, the current study compared EndoBRAIN's diagnostic performance against 30 endoscopists (20 trainees, 10 experts) using stained and narrow-band endocytoscopic images in a web-based trial. The authors found their CADx tool accurately differentiated neoplastic from non-neoplastic lesions, outperforming all endoscopists for stained images, achieving similar performance in narrow-band images, and being accepted for clinical use.

The third diagnostic model concerns the development of a deep learning model to predict the revised Vienna Classification in colonoscopy, which categorizes colorectal neoplasms into different levels of malignancy using standard endoscopic colonoscopy images [63]. Several CNN architectures were compared, namely AlexNet, ResNet152, and EfficientNet-B8, with ResNet152 being chosen as the prediction model due to its higher accuracy and fastest inference time. The model was trained using 56,872 colonoscopy images (6775 lesions) and validated on 255 images (128 lesions) from 7 external institutions in Japan. The authors also compared diagnostic performance against endoscopists (five novices, three fellows, and four experts). The AI system’s sensitivity and specificity exceeded that of all endoscopists. Nevertheless, the model cannot discriminate between high-grade dysplasia and invasive cancer (categories 4 and 5 of the revised Vienna Classification), and only binary classification is supported.

In the fourth document, the authors tested two pre-trained radiomics-based CNN architectures (Inception-ResNet-v2 and ResNet-152) to classify colorectal neoplasms into three types of sets automatically: 7-class (T1-4 colorectal cancer, high-grade dysplasia, tubular adenoma, vs. non-neoplasms), 4-class (neoplastic vs. non-neoplastic – advanced vs. early CRC vs. adenoma vs. healthy), and 2-class (neoplastic versus non-neoplastic and advanced versus non-advanced lesions) [64]. The CNNs were trained on a South Korean dataset (3453 colonoscopy images, 1446 patients) and temporally and geographically validated on 240 images (and as many patients) from another institution. CAM was used to highlight its decisions. The best-performing architecture was ResNet-152 for 7-way and 4-way diagnoses, but Inception-ResNet-v2 achieved better results on binary classifications. In addition, the model's performance was compared with one novice and two experienced endoscopists with six months and more than five years of colonoscopy experience, respectively. Although resulting in high accuracy, neither CNN architecture could outperform the endoscopists. Furthermore, this retrospective study only considered three types of diseases and white-light colonoscopy images.

The second set of articles was devoted to predicting outcomes from MRI scans in patients with colorectal cancer undergoing neoadjuvant chemotherapy (NCRT), accruing 143 MRIs from 118 patients and a mean AUC and accuracy of 0.77 and 81.9%, respectively [60, 65]. The first was a prospective study using a multipath CNN on MRI scans (diffusion kurtosis and T2-weighted) [60]. The authors used a dataset of 412 patients (290 for development and 93 for temporal validation) with locally advanced rectal adenocarcinoma scheduled for NCRT. The researchers developed three multipath CNN-based models: one to preoperatively predict pathologic complete response (pCR) to neoadjuvant chemoradiotherapy, one to assess tumor regression grade (TRG) (TRG0 and TRG1 vs. TRG2 and TRG3), and one to predict T downstaging. In addition, the authors evaluated the models' utility by comparing two radiologists' – with 10 and 15 years of experience – performance with and without their assistance. The results showed excellent performance in predicting pCR, superior to the assessment by the two radiologists, whose error rate was also reduced when assisted by the DL model. Although with lower performance, the TRG and T downstaging models also achieved promising results with an AUC of 0.70 and 0.79, respectively (although not outperforming the clinicians). Nevertheless, this monoinstitutional research required manual delineation, and interobserver variability was not analyzed. Moreover, further validation studies are necessary to assess performance with different MRI scanners.

The second group of researchers developed an MRI-based CNN (DC3CNN) to predict tumor regression grade (assessment of tumor size) in response to NCRT in patients with colorectal liver metastases [65]. The authors used prospective internal (328 lesions from 155 patients) and retrospective external cohorts (61 images, 25 patients) to collect pre and post-treatment T2-weighted- and DW-MRI scans. The model surpassed the diagnostic accuracy of RECIST, the most commonly used criteria for clinical evaluation of solid tumor response to chemotherapy. However, the study was retrospective, and further studies are needed to validate its performance in larger ethnically diverse patient populations.

Lastly, only one model assessed postoperative survival of colorectal cancer using text-based data [59]. The model was trained on the SEER database (364 316 patients) and externally validated (temporally and ethnically) on a Korean dataset (1 572 subjects, 607 women, 965 men). The authors compared 4 ML algorithms, namely logistic regression, DTs, RFs, and LightGBM, to obtain an optimal prognostic model. The best-performing model – LightGBM – outperformed TNM-7 in predicting survival for all tested periods (1, 2, 3, 4, 5, 6, 8, and 10 years). Still, data were collected retrospectively from a public database and a single institution using only text-based data, so prospective studies are necessary, and clinicopathological, molecular, and radiologic variables should also be incorporated.

##### Esophageal Cancer

Three studies involved esophageal cancers. Two papers studied neoplasia detection in patients with Barrett’s esophagus, a medical condition resulting from long-term acid-reflux damage, causing esophageal tissue lining to thicken and become irritated, increasing cancer risk [71, 73]. The same group of researchers conducted both studies: the first paper describes model development for detection [71], while the second encompasses its tuning and update to include location [73]. The authors proposed a multi-stage pretraining approach that involved training a CNN learning model on 494,355 gastrointestinal images before fine-tuning it on a smaller dataset of medical images specific to Barrett's neoplasia. The model was trained with images from different endoscopes. In the first paper [71], using data from separate institutions, the authors used a retrospective dataset of early Barrett’s neoplasia for primary validation (80 patients, unknown proportion) and a second prospectively acquired dataset (80 patients and images) to compare their model’s performance against fifty-three endoscopists (17 seniors, 8 juniors, 18 fellows, and 10 novices). In the second paper, the researchers validated their model on three prospective datasets: one with clinically representative images (80 individuals), one with subtle lesions (80 subjects), and one in a live setting with dysplastic and nondysplastic patients (ten each) [73]. It showed excellent performance on the three external validation datasets, and its detection and location performances were also compared against the 53 experienced endoscopists on the subtle lesions. The CAD system outperformed all 53 endoscopists for all tested metrics in both papers, obtaining an average accuracy, sensitivity, and specificity of 87.9%, 91.7%, and 84.16%, respectively. The models developed in both articles performed similarly and were tested in clinically realistic scenarios, with an average accuracy, sensitivity, and specificity of 88.45%, 91.25%, and 85.63%, respectively, enhancing CNNs’ predictive power.

Additionally, a retrospective study evaluated cancer-specific survival for esophageal adenocarcinoma and squamous cell carcinoma according to individual treatment recommendations [72]. The authors trained a deep-, regression-, and text-based survival neural network (DeepSurv, multi-layer perceptron) using the SEER database (6855 patients) and validated it on 150 women and 350 men from their institution (China). Additionally, prognostic performance was compared against TNM-8, having exceeded it. However, only one medical center was used, and research was not performed in an accurately representative clinical setting.

##### Gastric Cancer

In five articles, models were developed for gastric-related tasks. The first three studies had a diagnostic component. In the first research, the authors developed two models – GastroMIL and MIL-GC –, training them on WSIs from H&E slides magnified 30 times collected from TCGA and a Chinese institution. They also temporally and geographically validated them with 175 WSIs from 91 patients from NHGRP [67]. GastroMIL used an ensemble of a CNN and an RNN to distinguish gastric cancer from normal gastric tissue images. Its performance was compared against one junior and three expert pathologists. MIL-GC, a regression-based model, was created to predict patients’ overall survival. Besides WSIs, MIL-GC uses clinical data, namely survival state, overall survival time, age, sex, tumor size, neoplasm histologic grade, and pathologic T, N, M, and TNM-8 stages. The deep learning models achieved high performance in both tasks, with an overall accuracy of 92% for diagnosis and a C-index of 0.657 for prognosis prediction in the external dataset. Compared to human performance, GastroMIL outperformed the junior pathologist in accuracy and sensitivity but was surpassed by the experienced pathologists (in accuracy, sensitivity, and specificity). However, the tested cohorts were retrospective and had unbalanced survival times, and clinical utility was not evaluated for the prognostic model.

The second study used a CNN (ResNet-50) for real-time gastric cancer diagnosis [70]. The model was developed with 3 407 endoscopic images of 666 patients with gastric lesions from two institutions. The DCNN model was tested on a temporally different dataset of endoscopic videos from a separate institution (54 videos from 54 patients), and performance was compared against 20 endoscopists (6 experts, 14 novices). The model achieved better performance than any of the endoscopists, and diagnostic accuracy, sensitivity, and specificity increased for all clinicians while assisted by the model. Nevertheless, despite decreasing the aggregate diagnostic time from 4.35 s to 3.01 s, it increased experts’ by 0.10 s. In addition, the diagnostic model was only tested on high-quality images, and the validation dataset was small and domestic. Although slightly less sensitive than Gastro-MIL [67] (93.2% vs. 93.4%), the model developed in [70] achieved the best accuracy and sensitivity, evidencing that endoscopic images and videos might be more appropriate to diagnose gastric cancer.

The third model was created using endoscopic ultrasonography images (EUS) for the differential diagnosis of gastric mesenchymal tumors, including GISTs, leiomyomas, and schwannomas [66]. This model was trained with EUS from three Korean institutions and tested on a temporally separate set of 212 images from the same centers (69 patients, 38 female, 31 male). A sequential analysis approach was adopted using two CNNs: the first classifies the tumor as GIST or non-GIST; for non-GISTs, the second CNN classifies it as either a leiomyoma or schwannoma. The results were compared against junior (*n* = 3, less than 200 examinations) and expert endoscopists (*n* = 3, more than 500 examinations) who evaluated the same images, having surpassed them in both types of classification. However, this study was retrospective and involved a small number of patients, and the types of equipment used to perform ultrasounds varied considerably across the facilities.

The last two papers concerned outcome predictions. The first presents a multi-institutional study that uses multitask deep learning to predict peritoneal recurrence and disease-free survival in gastric cancer patients after curative-intent surgery based on CT images [68]. Supervised contrastive learning and a dynamic convolutional neural network were combined to achieve this purpose, and Grad-CAM was used to explain the model’s decisions. The model included CT scans from three patient cohorts, and external validation included 1 043 patients (329 women, 714 men) and as many images from another Chinese institution. In addition, the authors investigated clinician performance for peritoneal recurrence prediction with and without the assistance of the AI model, having found that performance was significantly enhanced after integrating it and that the model alone surpassed all physicians. Nonetheless, only East Asian patients were included in this retrospective study, which was not performed in a real clinical setting, and sensitivity was only reported for one of the clinicians.

The last study discusses the use of CT radiomics to predict the response of advanced gastric cancer to neoadjuvant chemotherapy and to detect pathological downstaging at an early stage [69]. The authors trained two SVCs on 206 patients who had undergone three or four cycles of chemotherapy and externally validated them on two testing cohorts, which were also used for benchmarking detection against RECIST. The first testing cohort consists of temporal validation (40 patients and CTs, 13 women, 27 men), while the second differs in the number of chemotherapy cycles (46 individuals and CTs, 10 women, 36 men). Performance for the detection model surpassed RECIST in both cohorts, and, except for sensitivity, the response prediction model also produced positive results. However, retrospective data and a small, unbalanced sample size constrain this study, which was not evaluated in a clinically representative setting.

##### Liver Cancer

Two models were developed for liver cancer-related predictions. The first aimed at classifying hepatocellular carcinomas and cholangiocarcinomas (differential diagnosis) [75]. The authors developed a web-based (cloud-deployed AI model and browser-based interface) CNN (DenseNet architecture) using WSIs from H&E slides magnified 40 times and used Grad-CAM to increase the model’s explainability. The training dataset was obtained from TCGA (70 slides from 70 unique patients). The external validation dataset was collected from the Department of Pathology at Stanford University Medical Center (80 slides from 24 women and 56 men). The model achieved a diagnostic accuracy of 84.2% in the validation cohort. Diagnostic performance was also compared to that of 11 pathologists. Except for the two unspecified pathologists, performance (AUC) increased for all clinicians when assisted by this tool. However, the pathologists only had access to the WSIs (as opposed to being complemented with clinical data), the model required manual intervention for patch selection, and the study was retrospective with a small sample size (development and external validation with a total of 150 WSIs and patients).

The second model was designed to predict three-year overall survival for intrahepatic cholangiocarcinoma patients after undergoing hepatectomy using an ensemble of Random Forests, XGBoost, and GBDT [74]. Using a single quaternary Chinese institution, the authors collected 1390 patients for training and 42 patients (12 women, 30 men) for external temporal validation. Results were compared against the TNM-8 and LCSGJ staging systems, with model performance exceeding that of the routinely used tools. Nonetheless, this was a monoinstitutional endeavor limited to a small number of Asian patients. Furthermore, only six prognostic factors were used: carcinoembryonic antigen, carbohydrate antigen 19–9, alpha-fetoprotein, pre-albumin, and T and N stages.

#### Endocrine system

Three papers described prognostic models for cancers in organs affecting the endocrine system (pancreas and thymus), whose results are depicted in Table 9.

**Table 9:** External validation and clinical utility performance for models evaluating primary cancers of the endocrine system

Cancer	Citations	Patients	Images	Outcome	Comparator	AUC	ACC	SEN	SPEC	PPV	NPV	C-index
**Pancreas**	Keyl [76]	22 (8♀, 14♂)	NA	Overall survival prediction	**Model (Random Survival Forest, text-based validation)**	NR	NR	NR	NR	NR	NR	**0.67**
					mGPS	NR	NR	NR	NR	NR	NR	0.59
	Lee [77]	53 (27♀, 26♂)	53 CECTs	2-year Overall Survival	**Model (ANN + logistic regression + RF + GB + SVM + CNNs, CECTs + clinical data)**	**0.76**	NR	69	**83.3**	**83.3**	69	NR
					TNM-8	0.67	NR	**86.2**	37.5	62.5	**69.2**	NR
				1-year Recurrence-Free Survival	Model (ANN + logistic regression + RF + GB + SVM + CNNs, CECTs + clinical data)	**0.744**	NR	74.1	**65.4**	**69**	**70.8**	NR
					TNM-8	0.541	NR	**81.5**	30.8	55	61.5	NR
**Thymus**	Feng [78]	76 (33♀, 48♂)	76 NECTs	Differential Diagnosis—LRT vs. HRT + TC	**Model (SVM, NECTs + clinical data)**	**0.844**	71.05	**82.86**	60.98	NR	NR	NR
					Radiologist 1 (3 years of experience)	0.645	63.16	54.29	70.73	NR	NR	NR
					**Radiologist 2 (6 years of experience)**	0.724	**76.32**	80	**73.17**	NR	NR	NR
					Radiologist 3 (12 years of experience)	0.813	67.11	65.71	68.29	NR	NR	NR
				Differential Diagnosis—HRT vs. LRT + TC	**Model (SVM, NECTs + clinical data)**	**0.844**	68.42	55.26	**81.58**	NR	NR	NR
					Radiologist 1 (3 years of experience)	0.645	55.26	55.26	55.26	NR	NR	NR
					**Radiologist 2 (6 years of experience)**	0.724	**76.32**	**73.68**	78.95	NR	NR	NR
					Radiologist 3 (12 years of experience)	0.813	64.47	65.79	63.16	NR	NR	NR
				Differential Diagnosis—TC vs. LRT + HRT	**Model (SVM, NECTs + clinical data)**	**0.844**	94.74	33.33	97.26	NR	NR	NR
					Radiologist 1 (3 years of experience)	0.645	86.84	0	90.41	NR	NR	NR
					Radiologist 2 (6 years of experience)	0.724	97.37	33.33	100	NR	NR	NR
					Radiologist 3 (12 years of experience)	0.813	94.74	0	**98.63**	NR	NR	NR

*ACC* Accuracy, *ANN* Artificial Neural Network, *AUC* Area Under the ROC Curve, *CECT* Constrast-Enhanced CT, *CNN* Convolutional Neural Network, *CT* Computed Tomography, *GB* Gradient Boosting, *HRT* How-Risk Thymoma, *LRT* Low-Risk Thymoma, *mGPS* Modified Glasgow Prognostic Score, *NECT* Non-Contrast Enhanced CT, *NPV* Negative Predictive Value, *NR* Not Reported, *PPV* Positive Predictive Value, *RF* Random Forest, *ROC* Receiver Operating Characteristic, *SEN* Sensitivity, *SPEC* Specificity, *SVM* Support Vector Machine, *TC* Thymic Cancer, *TNM* Tumor, Node, and Metastasis staging system, *TNM-8* Eighth edition of the TNM staging system

##### Pancreatic Cancer

The first two studies assessed survival for pancreatic ductal adenocarcinoma (PDAC) patients but adopted disparate research designs and clinical inputs [76, 77].

The first group of researchers used a regression-based random survival forest model to prognosticate patients with advanced pancreatic cancer [76]. Aimed at predicting overall survival for patients with unresectable PDAC, the model was developed with clinical data and CT scans from a German institution (203 patients). It was temporally and geographically validated using only text-based clinical data from patients with liver metastases from the same country (8 women, 14 men) and compared against mGPS, having outperformed it. Additionally, the authors used SHAP to explain their model, finding that inflammatory markers C-reactive protein and neutrophil-to-lymphocyte ratio had the most significant influence on its decision-making. Nonetheless, only twenty national patients were used to validate the model externally, and different types of inputs were used for training and testing.

The second set of authors used an ensemble of ML methods – ANN, logistic regression, RF, GB, SVM, and CNNs (3D ResNet-18, R(2 + 1)D-18, 3D ResNeXt-50, and 3D DenseNet-121) – to predict 2-year overall and 1-year recurrence-free survival for PDAC patients after surgical resection [77]. The classifier was trained and tuned using 229 patients and temporally validated with CECT images and seventeen clinical variables from the same South Korean institution (53 CECTs from 27 women and 26 men). Grad-CAM was used to explain the model’s decisions, and comparisons were made against TMN-8 to evaluate clinical utility. Although more accurate, specific, and with a higher PPV than TNM-8, it was less sensitive for both predictions and had a lower NPV for overall survival prediction. Furthermore, tumor margins were manually segmented, and the model did not consider histopathologic data.

##### Thymic Cancer

One study was designed for the simplified risk categorization of thymic epithelial tumors (TETs), rare cancer forms [78]. Here, three types of tumors were evaluated: low-risk thymoma (LRT), high-risk thymoma (HRT), and thymic carcinoma (TC). Three triple classification models were developed using radiomic features extracted from preoperative NECT images and clinical data from 433 patients: (i) LRT vs. HRT + TC; (ii) HRT vs. LRT + TC; (iii) TC vs. LRT + HRT. The authors compared several CT-based classifiers: logistic regression, linear SVC, Bernoulli and Gaussian Naïve Bayes, LDA, Stochastic Gradient Descent, SVM, DT, kNN, MLP, RF, AdaBoost, gradient boosting, and XGBoost. Combined with clinical data, the SVM model demonstrated the best performance for predicting the simplified TETs risk categorization. In addition, the SVM model was validated in a temporally different cohort using images from 5 types of scanners (76 scans and patients, 33 women, 48 men). Finally, its diagnostic performance was compared against three radiologists (3, 6, and 12 years of experience), having exceeded them regarding AUC (0.844 versus 0.645, 0.813, and 0.724) but not for other metrics (accuracy, sensitivity, and specificity). Caveats include the reduced amount of patients, low number of thymic carcinomas, and incomplete automation of the models.

#### Genitourinary system

Table 10 illustrates the models developed for genitourinary cancers, including the bladder, cervix, prostate, and uterus.

**Table 10:** External validation and clinical utility performance for models evaluating primary cancers of the genitourinary system

Cancer	First Author	Patients	Images	Outcome	Comparator	AUC	ACC	SEN	SPEC	PPV	F1-Score	Brier Score	MAE	MDT (s)
**Bladder**	Zhang [79]	75 (13♀, 62♂)	75 CECTs	Muscular invasiveness	Model (CNN, CECT images + clinical data)	0.791	**74.7**	71	77.3	NR	NR	NR	NR	NR
					**Radiologist 1 (9 years of experience)**	NR	**74.7**	77.4	**72.7**	NR	NR	NR	NR	NR
					Radiologist 2 (3 years of experience)	NR	57.3	**83.9**	38.6	NR	NR	NR	NR	NR
**Cervix**	Cheng [80]	1170♀	1170 WSIs (with unknown stain) magnified 40 times	Detection and diagnosis (binary, neoplastic vs. non-neoplastic) – Women without comorbidities	**CNN + RNN (WSIs)—women without conditions**	**0.979**	NR	95.1	93.5	NR	NR	NR	NR	90 s per gigapixel WSI
					**Cytopathologists (*n* = 3)**	**0.82**	NR	NR	NR	NR	NR	NR	NR	NR
		395♀	395 WSIs (with unknown stain) magnified 40 times	Detection and diagnosis (binary, neoplastic vs. non-neoplastic) – HPV-positive women	CNN + RNN (WSIs)	0.89	NR	79.3	81.9	NR	NR	NR	NR	90 s per gigapixel WSI
**Prostate**	Mehta [82]		210 mpMRI scans	Diagnosis (binary, CSPCa vs. non-CSPCa)	**Model (CNN, MRI-based)—Post-thresholding (cut-off: > = 4.5%)**	**0.7**	**NR**	**78**	49	78	NR	NR	0.019	NR
		247			**Model (CNN, MRI-based)—Post-thresholding (cut-off: > = 4.5%) and false-positive reduction (< 40 mm**^**3**^**)**	**0.7**	**NR**	76	**57**	**80**	NR	NR	0.019	NR
					Expert Radiologist (10 years of experience)	0.64	NR	78	48	78	NR	NR	0.031	NR
	Varghese [81]	14♂	14 mpMRI scans	Risk Stratification—High-risk patients	**Model (QSVM, MRI-based)**	0.71	NR	**86**	NR	**57**	**0.69**	NR	NR	NR
					PI-RADS v2	**0.73**	NR	61	NR	45	0.52	NR	NR	NR
		39♂	39 mpMRI scans	Risk Stratification—Low-risk patients	Model (QSVM, MRI-based)	0.71	NR	**78**	NR	**94**	**0.85**	NR	NR	NR
					PI-RADS v2	**0.73**	NR	**78**	NR	87	0.82	NR	NR	NR
**Uterus**	Bi [115]	44♀	44 T1-weighted, T2-weighted, and DWI MRI scans	Diagnosis (binary, malignant vs. benign)	Radiomics model only	0.798	**75**	73.1	**83.3**	NR	NR	0.188	NR	NR
					Nomogram: clinical model + radiomic score	**0.802**	72.7	73.1	77.8	NR	NR	0.184	NR	NR
					Stacking model: clinical data + radiomics model + multivariate logistic regression	0.792	77.7	76.9	77.8	NR	NR	**0.182**	NR	NR
					Ensemble model: clinical model + radiomics model + weighted accuracy average	0.794	70.5	76.9	77.8	NR	NR	0.184	NR	NR
					Radiologist (30 years of experience)	0.628	68.2	**92.3**	33.3	NR	NR	NR	NR	NR
	Zhao [83]	102♀	1631 H&E-stained histological images	Diagnosis (binary, endometrium hyperplasia vs. intraepithelial neoplasia)	Model (CNN, WSIs)	NR	95.34	95.34	NR	95.67	0.9531	NR	NR	NR
					**Senior Pathologist (25 years of experience)**	**NR**	**98.65**	**98.52**	**NR**	**98.66**	**0.9859**	NR	NR	NR
					Mid-Level Pathologist (6 years of experience)	NR	95.71	95.48	NR	95.77	0.956	NR	NR	NR
					Junior Pathologist (2 years of experience)	NR	85.04	85.04	NR	85.7	0.8501	NR	NR	NR

*ACC* Accuracy, *AUC* Area Under the ROC Curve, *CECT* Constrast-Enhanced CT, *CNN* Convolutional Neural Network, *CSPCa* Clinically Significant Prostate Cancer, *CT* Computed Tomography, *DWI* Diffusion-Weighted Imaging, *H&E* Hematoxylin and Eosin, *HPV* Human Papillomavirus, *MAE* Mean Average Error, *MDT* Mean Diagnostic Time, *mpMRI* Multiparametric MRI, *MRI* Magnetic Resonance Imaging, *NECT* Non-Contrast Enhanced CT, *NR* Not Reported, *PI-RADS* Prostate Imaging Reporting and Data System, *PPV* Positive Predictive Value, *QSVM* Quadratic Support Vector Machine, *RNN* Recurrent Neural Network, *ROC* Receiver Operating Characteristic, *WSI* Whole-Slide Image

##### Bladder Cancer

From the retrieved models, only one assesses outcomes for primary bladder cancers [79]. This article presents a CNN-based strategy to predict the muscular invasiveness of bladder cancer based on CT images and clinical data. The model was developed with 183 patients. Its performance was tested on an independent institution's temporally and geographically different validation cohort of patients with urothelial carcinoma (13 women, 62 men, and as many images). The model’s predictions were juxtaposed with diagnoses from two radiologists with nine and two years of experience, having achieved better accuracy and specificity than the two clinicians but a lower sensitivity. Overall, the authors found that the deep learning algorithm achieved a high accuracy rate in predicting muscular invasiveness, an essential factor in determining the prognosis and treatment of bladder cancer. However, the study is limited by its retrospective nature, exclusion of tumors not visible in CT images, and small sample size.

##### Cervical Cancer

Similarly, primary tumors of the cervix were only screened in one paper [80]. Here, the authors trained an ensemble of convolutional and recurrent neural networks on whole-slide images from patients' cervical biopsies and 79 911 annotations from five hospitals and five kinds of scanners. The system comprises (i) two CNNs – the first scans WSIs at low resolution and the second at high resolution – to identify and locate the ten most suspicious areas in each slide; (ii) and an RNN to predict corresponding probabilities. The system classifies squamous and glandular epithelial cell abnormalities as positive (neoplastic) and normal findings as negative for intraepithelial lesions or malignancies (non-neoplastic). The method was externally validated on multi-center independent test sets of 1 565 women (1 170 without additional conditions and 395 with HPV), and classification performance was compared against three cytopathologists. Although obtaining promising results and surpassing clinician performance for both types of women, the authors highlight that the model was designed for the general women population, implying that further refinements are required for specific comorbidities.

##### Prostate Cancer

Two models were developed for prostate-cancer-related classifications using multiparametric MRI scans [81, 82]. In the first paper, the authors describe the development of Autoprostate, a system employing deep learning to generate a report summarizing the probability of suspicious lesions qualifying as clinically significant prostate cancer (CSPCa) [82]. The authors trained their approach on the PROSTATEx dataset (249 men), externally validated it on the PICTURE dataset (247 patients), and compared its reports (with post-thresholding and false positive reduction) to those generated by a radiologist with ten years of experience. The system achieved a high level of agreement with the human reports (surpassing the radiologist in AUC and specificity) and could accurately identify CSPCa. However, this study was retrospective, a single (public) dataset was used for external validation, and only two types of prostate lesions were considered.

The second article presented an ML-based approach for prostate cancer risk stratification using radiomics applied to multiparametric MRI scans [81]. In this retrospective, monoinstitutional study, the authors compared seven classification algorithms: logistic regression, linear, quadratic (Q), cubic, and Gaussian kernel-based SVM, linear discriminant analysis, and RF. After training with 68 patients, the best-performing method – QSVM – was validated on a temporally independent dataset (14 high- and 39 low-risk patients). Its performance was compared against PI-RADS v2, having found that the model could accurately predict the risk of clinically significant prostate cancer. Although the classifier performed equivalently to PI-RADS v2 regarding AUC, it performed substantially better in class-specific measures (F1-score, sensitivity, and PPV), especially for the high-risk class. However, the study is limited by its retrospective nature and small sample size from a single source.

##### Uterine Cancer

Two studies for primary cancers focused on classifying lesions of the endometrium, the layer of tissue lining the uterus [83, 115]. In the first article, using 245 women as the training cohort, the authors compared nine models – logistic regression (LR), SVM, stochastic gradient descent, kNN, DT, RF, ExtraTrees, XGBoost, and LightGBM – to obtain an optimal algorithm for differential diagnosis (malignant versus benign tumors) [115]. A radiomics score (radscore) was computed for the best-performing algorithm (logistic regression), and four models were selected using different combinations of T1-weighted, T2-weighted, and DWI MRI features: (i) the radiomics model; (ii) a nomogram, combining the radscore and clinical predictive parameters; (iii) a two-tiered stacking model, where the first tier was the clinical model and the optimal radiomics model (LR), and the second tier used the output of the first tier as the input of the multivariate LR; and (iv) an ensemble model, where the predictions obtained from the preceding clinical model and radiomics model were calculated by an accuracy-weighted average. The results showed that all four models accurately differentiated stage IA endometrial cancer and benign endometrial lesions. Furthermore, during external validation (44 MRIs from 44 women), the authors found that the nomogram had a higher AUC than the radiomics model, revealing more stable discrimination efficiency and better generalizability than the stacking and ensemble models and a radiologist with 30 years of experience (except in sensitivity). Nevertheless, data was collected from two same-country centers (Chinese institutions), only standard radiomics features were extracted, and lesions were manually segmented, which is highly time-consuming.

The second paper encompassed a global-to-local multi-scale CNN to diagnose endometrial hyperplasia and screen endometrial intraepithelial neoplasia (EIN) in histopathological images [83]. The researchers trained the CNN using a large annotated dataset (6 248 images) and tested it on a temporally different set of patients (1631 images, 135 specimens, 102 women). They found that it performed well in diagnosing endometrial hyperplasia and detecting EIN, outperforming a junior pathologist (2 years of experience) and obtaining comparable performance to a mid-level and a senior pathologist (6 and 25 years of experience, respectively). The authors used Grad-CAM to emphasize the regions the model deemed relevant for diagnosis. However, this retrospective study only used histopathological images (as opposed to WSIs). Besides, it focused solely on classifying healthy slides, hyperplasia without atypia, and endometrial intraepithelial neoplasia, thus neglecting the differentiation between benign lesions and endometrial cancer.

#### Integumentary system

As illustrated in Table 11, five papers studied cancers of the integumentary system, focusing on the breasts and skin.

**Table 11:** External validation and clinical utility performance for models evaluating primary cancers of the integumentary system

Cancer	First Author	Patients	Images	Outcome	Comparator	AUC	ACC	SEN	SPEC	PPV	NPV	F1-Score
**Breast**	Ji [84]	150♀	NA	Osteoporosis Risk	**Model (XGBoost, text-based)**	**0.87**	**87**	**87**	NR	**86**	NR	**0.86**
					FRAX	0.72	NR	NR	NR	NR	NR	NR
					OSTA	0.66	NR	NR	NR	NR	NR	NR
				Fracture Risk	**Model (XGBoost, text-based)**	**0.92**	**93**	**93**	NR	**91**	NR	**0.91**
					FRAX	0.86	91	91	NR	90	NR	0.91
				Overall Survival (8y)	**Model (XGBoost, text-based)**	**0.96**	**92**	**92**	NR	**92**	NR	**0.92**
					TNM-8	0.87	87	87	NR	86	NR	0.85
	Leibig [85]	92 585♀	213,694 mammographic images	Diagnosis (binary, malignant vs. non-malignant)	Model (CNN, image-based)	0.951	NR	84.6	91.3	NR	NR	NR
					Radiologist for AI alone	NR	NR	87.2	93.4	NR	NR	NR
					**Decision referral (AI + Radiologist) **^a^	**NR**	**NR**	**90.7**	**93.7**	NR	NR	NR
	Romeo [86]	57♀	66 ultrasounds	Diagnosis (binary, malignant vs. benign)	**Model (RF, image-based)**	**0.82**	**82**	**93**	57	82	**80**	NR
					Radiologist (8y) without assistance	NR	79.4	77.8	81	89.7	63	NR
					**Radiologist (8y) with assistance**	NR	80.2	88.9	**71.4**	**87**	75	NR
**Skin**	Han [87]	10,426 (4855♀, 4701♂)	40,331 photographs	Diagnosis (binary, malignant vs. non-malignant, 43 recognized conditions)	Model (CNNs, photograph-based)—high-sensitivity threshold	0.863	NR	79.1	76.9	31.3	96.5	NR
					**Physicians (*n* = 65)—high-sensitivity threshold**	**NR**	**NR**	**88.1**	**83.8**	**41.9**	**98.1**	**NR**
					Model (CNNs, photograph-based)—high-specificity threshold	0.863	NR	62.7	90	45.4	94.8	NR
					**Physicians (*n* = 65)—high-specificity threshold**	**NR**	**NR**	**70.2**	**95.6**	**68.1**	**96**	**NR**
		10,315^b^ (4838♀, 4626♂)	39,721 photographs	Diagnosis (multiclass, 32 conditions)	Model (CNNs, photograph-based)—South Korean Dataset	0.931	NR	NR	NR	NR	NR	NR
		NR	1300 photographs	Diagnosis (multiclass, 10 conditions)	Model (CNNs, photograph-based)—Edinburgh Dataset	0.939	NR	NR	NR	NR	NR	NR
		1320^b^ (740♀, 580♂)	5065 photographs	Diagnosis (binary, malignant vs. non-malignant, 31 conditions)	**Model (CNNs, photograph-based)—high-sensitivity threshold**	**0.869**	**NR**	**85.3**	**75.2**	**NR**	**NR**	**NR**
					Dermatologists (*n* = 44)—high-sensitivity threshold	NR	NR	84.9	66.9	NR	NR	NR
					**Model (CNNs, photograph-based)—high-specificity threshold**	**0.869**	**NR**	**66.9**	**87.4**	NR	NR	NR
					Dermatologists (*n* = 44)—high-specificity threshold	NR	NR	65.8	85.7	NR	NR	NR
	Li [88]	176 (111♀, 65♂)	266 photographs	Detection (location) and diagnosis (binary, malignant vs. benign)	**Model (CNNs, photograph-based)**	**0.899**	**81.8**	**91.5**	79.2	NR	NR	NR
					Junior Ophthalmologist (3 years of experience)	NR	72.3	66.1	78.8	NR	NR	NR
					Junior Ophthalmologist (7 years of experience)	NR	77.9	74.6	78.8	NR	NR	NR
					Expert Ophthalmologist (15 years of experience)	NR	81.8	91.5	79.2	NR	NR	NR

*AI* Artificial Intelligence, *ACC* Accuracy, *AUC* Area Under the ROC Curve, *CNN* Convolutional Neural Network, *FRAX* Fracture Risk Assessment Tool, *NA* Not Applicable, *NPV* Negative Predictive Value, *NR* Not Reported, *OSTA* Osteoporosis Self-Assessment Tool for Asians, *PPV* Positive Predictive Value, *RF* Random Forest, *ROC* Receiver Operating Characteristic, *SEN* Sensitivity, *SPEC* Specificity, *TNM* Tumor, Node, and Metastasis staging system, *TNM-8* Eighth edition of the TNM staging system, *XGBoost* Extreme Gradient Boosting

^a^This example was selected due to having the largest average increase of sensitivity and specificity (without sensitivity and specificity suffering loss)

^b^ Subset of the first dataset

##### Breast Cancer

Three studies developed models for cancers originating in the breasts, each with a specific purpose and using different clinical modalities. In [84], several text-based machine learning classifiers, namely, DTs, RFs, MLPs, logistic regression, naïve Bayes, and XGBoost, were compared to establish optimal classifiers for osteoporosis, relative fracture, and 8-year overall survival predictions. The algorithm was trained on 420 patients from a Chinese institution and geographically validated on 150 women from a separate local institution. The osteoporosis model was compared against OSTA and FRAX, the fracture model against FRAX, and the prognostic model against TNM-8. The results showed that the XGBoost classifier performed the best for the three tasks and outperformed the other clinical models. Additionally, for explainability, the authors also used SHAP for feature importance analysis for each model: (i) age, use of anti-estrogens, and molecular type are the most predictive of osteoporosis; (ii) osteoporosis, age, and bone-specific alkaline phosphatase are the best predictors for fracture; and (iii) N-stage, molecular type, and age have the highest prognostic value for overall survival. Despite its positive results, prospective studies are needed to validate the model in more diverse patient populations.

In [85], authors explored how combining AI and radiologists can improve breast cancer screening. Using 213 694 retrospectively collected mammograms (X-ray images) from 92 585 women, it was found that the combination of radiologists and AI (CNN-based classifier) achieved the highest accuracy in detecting breast cancer. The sensitivity and specificity of the standalone AI system were significantly lower than an unaided radiologist. However, the decision-referral approach outperformed the unaided radiologist on both sensitivity and specificity for several tested thresholds. Nonetheless, the study only included mammogram images and did not consider other factors, such as patient history or clinical data, which may impact the accuracy of breast cancer screening. Furthermore, the AI algorithm used in the study was not optimized for clinical use and may require further development and testing before it can be implemented in a clinical setting.

Lastly, the work developed in [86] entailed diagnosing non-cystic benign and malignant breast lesions from ultrasonographic images. Radiomic features were extracted from the ultrasound images, and a random forest model was trained with 135 lesions and externally validated to predict malignancy for each lesion. Moreover, the performance of an experienced radiologist (8 years) was compared with and without the model’s assistance. Although not with statistical significance, the radiologist's assessments improved when using the AI system. However, the final validation population was small (66 ultrasounds from 57 women) and showed different proportions of malignant lesions.

##### Skin Cancer

Two models were developed to diagnose skin tumors using photographs, producing an average AUC, sensitivity, and specificity of 0.89, 77.1%, and 81.74% [87, 88]. The first was a retrospective validation study assessing the performance of deep neural networks in detecting and diagnosing benign and malignant skin neoplasms of the head and neck, trunk, arms, and legs [87]. In a previous study, the authors trained an ensemble of CNNs (SENet + SE-ResNeXt-50 + faster RCNN) with 1 106 886 image crops from South Korean patients to detect potential lesions and classify skin malignancies. Here, performance was tested on three new temporal and geographical validation datasets of skin lesions (two national, one international, 46 696 photographs from 10 876 patients): (i) one dataset was used to compare the model’s classification performance against 65 attending physicians in real-world practice; (ii) one’s goal was to evaluate classification performance against with 44 dermatologists in an experimental setting; and (iv) the last two were meant to predict exact diagnosis (1 of 43 primary skin neoplasms) in a local (South Korean) and an international (UK, 1 300 images) dataset, with the first also being compared against physicians. In (i) and (ii), performance was calculated for high specificity and high sensitivity thresholds. The algorithm was more sensitive and specific than the dermatologists in the experimental setting. However, attending physicians outperformed it in real-world practice in all tested metrics (sensitivity, specificity, PPV, and NPV). In addition, the model only dealt with high-quality clinical photographs, and there was a lack of ethnic diversity in the study population.

The second paper presented a set of CNNs – DenseNet-121 (Faster R-CNN and deep classification network) – developed to detect malignant eyelid tumors from photographic images [88]. The researchers used a 1 417 clinical images dataset with 1 533 eyelid tumors from 851 patients across three Chinese institutions (one for development and two for external validation). Besides using Grad-CAM for interpretation, the AI’s performance on the external dataset (266 pictures from 176 patients) was compared to three ophthalmologists: one junior, one senior, and one expert (3, 7, and 15 years of experience, respectively). It surpassed the junior and senior ophthalmologists’ performance and achieved similar results to the expert. Notwithstanding its potential, the system still needs evaluation on non-Asian populations and prospectively acquired datasets, and it was only developed for detection (it cannot provide a specific diagnosis).

#### Respiratory system and associated tissues

Thirteen papers addressed respiratory system cancers, which predominantly concerned the lungs, but also included the larynx, nasopharynx, and mesothelium (Table 12).

**Table 12:** External validation and clinical utility performance for models evaluating primary cancers of the respiratory system and associated tissues

Cancer	First Author	Patients	Images	Outcome	Comparator	JAF-ROC		AUC	ACC	SEN	SPEC	PPV	NPV	FPR	F1-Score	C-index	Brier Score	PT (s)
**Larynx**	Xiong [99]	392	1176 white-light endoscopic images	Diagnosis (binary, urgent (LCA and PRELCA) vs. non-urgent (BLT and healthy)	CNN, GoogLeNet Inception v3, white-light endoscopic images)	NR		0.953	89.7	72	**94.8**	NR	NR	NR	NR	NR	NR	NR
					Endoscopist 1 (10 – 20 years of experience)	NR		NR	**90.6**	**76.1**	94.6	NR	NR	NR	NR	NR	NR	NR
					Endoscopist 2 (3 years of experience)	NR		NR	81.7	**87.5**	80.1	NR	NR	NR	NR	NR	NR	NR
					Endoscopist 3 (3—10 years of experience)	NR		NR	85.8	70.2	90.2	NR	NR	NR	NR	NR	NR	NR
				Diagnosis (4-way classification, LCA vs. PRELCA vs. BLT vs. healthy)	CNN, GoogLeNet Inception v3, white-light endoscopic images)	NR		NR	**77.3**	NR	NR	NR	NR	NR	NR	NR	NR	NR
					Endoscopist 1 (10 – 20 years of experience)	NR		NR	75	NR	NR	NR	NR	NR	NR	NR	NR	NR
					Endoscopist 2 (3 years of experience)	NR		NR	64.7	NR	NR	NR	NR	NR	NR	NR	NR	NR
					Endoscopist 3 (3—10 years of experience)	NR		NR	70.4	NR	NR	NR	NR	NR	NR	NR	NR	NR
**Lung**	Baldwin [96]	1187	1397 CECT and NECT images	Diagnosis (binary, malignant vs. non-malignant)	Model (CNN, CT-based)	NR		**0.896**	NR	**99.57**	28.03	NR	NR	NR	NR	NR	NR	NR
					Brock Model	NR		0.868	NR	97.44	**29.23**	NR	NR	NR	NR	NR	NR	NR
	Chen [91]	220 (136♀, 84♂)	583 CT images	Diagnosis (binary, malignant vs. non-malignant)	**Model (XGBoost, CT images + clinical data)**	**NR**		**0.89**	**NR**	**NR**	**NR**	**NR**	**NR**	**NR**	**NR**	**NR**	**0.1426**	**NR**
					Brock model	NR		0.806	NR	NR	NR	NR	NR	NR	NR	NR	0.4	NR
					PKU model	NR		0.78	NR	NR	NR	NR	NR	NR	NR	NR	0.216	NR
					Mayo model	NR		0.739	NR	NR	NR	NR	NR	NR	NR	NR	0.366	NR
					VA model	NR		0.682	NR	NR	NR	NR	NR	NR	NR	NR	0.37	NR
		195 (110♀, 85♂)	195 CT images	Diagnosis (binary, malignant vs. non-malignant)—generalization cohort	**Model (XGBoost, CT images + clinical data)**	NR		**0.871**	NR	NR	NR	NR	NR	NR	NR	NR	NR	NR
					Brock model	NR		0.78	NR	NR	NR	NR	NR	NR	NR	NR	NR	NR
					PKU model	NR		0.812	NR	NR	NR	NR	NR	NR	NR	NR	NR	NR
					Mayo model	NR		0.762	NR	NR	NR	NR	NR	NR	NR	NR	NR	NR
					VA model	NR		0.681	NR	NR	NR	NR	NR	NR	NR	NR	NR	NR
		78 (51♀, 27♂)	200 CT images (prospective)	Diagnosis (binary, malignant vs. non-malignant)—prospective cohort	**Model (XGBoost, CT images + clinical data)**	NR		**0.871**	NR	79.4	75.7	NR	NR	NR	NR	NR	0.1426	NR
					Thoracic Surgeons, *n* = 3, 3, 5, and 10 years of experience)	NR		0.753	NR	NR	NR	NR	NR	NR	NR	NR	NR	NR
					Radiologist (*n* = 1, 5 years of experience)	NR		0.748	NR	NR	NR	NR	NR	NR	NR	NR	NR	NR
	Choi [89]	141 (84♀, 57♂)	141 CECTs	Prediction of visceral invasion	Model (CNN, image-based, high sensitivity cutoff (0.245) [90%])	NR		61	NR	93.8	31.2	41.3	90.6	NR	NR	NR	NR	NR
					Model (CNN, image-based, high specificity cutoff (0.245) [90%])	NR		64	NR	47.9	86	**63.9**	76.2	NR	NR	NR	NR	NR
					Model (CNN, image-based, optimal diagnostic performance (0.372))	NR		75	NR	62.5	68.8	50.8	78	NR	NR	NR	NR	NR
					Radiologist 1 (9 years of experience)	NR		NR	NR	93.8	48.4	48.4	93.8	NR	NR	NR	NR	NR
					Radiologist 2 (4 years of experience)	NR		NR	NR	**97.9**	40.9	46.1	**97.4**	NR	NR	NR	NR	NR
					Radiologist 3 (4 years of experience)	NR		NR	NR	62.5	**69.9**	51.7	78.3	NR	NR	NR	NR	NR
	Deng [97]	92 (52♀, 40♂)	92 CT images	Prediction of progression-free survival (survival benefit prediction)	**Model (CNN, image-based)**	**NR**		**0.76**	**76.08**	**85.71**	**61.11**	**77.43**	**73.33**	NR	NR	0.69	NR	55 s/8 s ^1^
					Radiologist 1 (Trainee, 2 years of experience)—Unaided	NR		NR	47.93	42.51	45.83	34.43	54.37	NR	NR	NR	NR	NR
					**Radiologist 1 (Trainee, 2 years of experience)—Aided**	**NR**		NR	**68.5**	**43.69**	**89.13**	**71.56**	**71.66**	NR	NR	NR	NR	NR
					Radiologist 2 (Competent, 5 years of experience)—Unaided	NR		NR	51.03	59.95	45.83	42.54	63.11	NR	NR	NR	NR	NR
					**Radiologist 2 (Competent, 5 years of experience)—Aided**	**NR**		NR	**60.83**	**62.67**	**71.43**	**49.13**	**65.84**	NR	NR	NR	NR	NR
					Radiologist 3 (Expert, 10 years of experience)—Unaided	NR		NR	65.09	55.98	67.39	51.8	70.97	NR	NR	NR	NR	NR
					**Radiologist 3 (Expert, 10 years of experience)—Aided**	**NR**		NR	**70.7**	**74.44**	**69.57**	**60.49**	**81.3**	NR	NR	NR	NR	NR
					Oncologist 1 (Trainee, 2 years of experience)—Unaided	NR		NR	47.9	**50.02**	47.83	37.51	60.45	NR	NR	NR	NR	NR
					**Oncologist 1 (Trainee, 2 years of experience)—Aided**	**NR**		NR	**64.13**	46.74	**73.91**	**52.87**	**68.91**	NR	NR	NR	NR	NR
					Oncologist 2 (Competent, 5 years of experience)—Unaided	NR		NR	55.2	26.19	74.47	40.03	60.79	NR	NR	NR	NR	NR
					**Oncologist 2 (Competent, 5 years of experience)—Aided**	**NR**		NR	**61.95**	**30.92**	**78.26**	**47.11**	**64.41**	NR	NR	NR	NR	NR
					Oncologist 3 (Expert, 10 years of experience)—Unaided	NR		NR	65.23	70.95	52.21	49.55	76.9	NR	NR	NR	NR	NR
					**Oncologist 3 (Expert, 10 years of experience)—Aided**	**NR**		NR	**75.2**	**71.25**	**78.26**	**65.27**	**78.25**	NR	NR	NR	NR	NR
	Hindocha [92]	159 (71♀, 88♂)	NA	Recurrence	**Model (kNN + NB + RF, text-based)**	**NR**		**0.722**	**69.2**	**63**	**72.4**	**54**	**79.2**	**NR**	**0.581**	NR	**0.2**	**NR**
					TNM-8	NR		0.707	NR	NR	NR	NR	NR	NR	NR	NR	NR	NR
					WHO Performance Status	NR		0.584	NR	NR	NR	NR	NR	NR	NR	NR	NR	NR
				Recurrence-free survival	Model (kNN, text-based)	NR		0.681	63.5	40.5	83.5	68.2	61.7	NR	0.508	NR	0.32	NR
					**TNM-8**	**NR**		**0.695**	NR	NR	NR	NR	NR	NR	NR	NR	NR	NR
					WHO Performance Status	NR		0.499	NR	NR	NR	NR	NR	NR	NR	NR	NR	NR
				Overall Survival (2Y)	**Model (XGBoost + NNET + MDA, text-based)**	**NR**		**0.717**	**64.8**	**75.9**	**59.1**	**48.8**	**82.7**	**NR**	**0.594**	NR	**0.2**	**NR**
					TNM-8	NR		0.665	NR	NR	NR	NR	NR	NR	NR	NR	NR	NR
					WHO Performance Status	NR		0.531	NR	NR	NR	NR	NR	NR	NR	NR	NR	NR
	Lu [94]	5493 (2456♀, 3037♂)	5493 low-dose CT images	12-year lung cancer risk incidence model	**Model (CNN, mixed)**	**NR**		**0.755**	NR	**74.9**	**63.6**	**7.3**	**98.5**	NR	NR	NR	NR	NR
					PLCOm2012	NR		0.761	NR	74.4	63.5	7.2	94.8	NR	NR	NR	NR	NR
	Nam [90]	181 (82♀, 99♂)	181 CECTs	Detection	Model (CNN, CT-based)—1/4 institutions (South Korea)	0.885		NR	NR	69.9	NR	NR	NR	34	NR	NR	NR	NR
				Diagnosis (binary, malignant vs. non-malignant)	**Model (CNN, CT-based)—1/4 institutions (South Korea)**	NR		**0.92**	NR	79	95	94.9	NR	NR	0.87	NR	NR	NR
					Radiology residents (*n* = 6, 1—3 years of experience)—Unaided (Center 1)	NR		0.852	NR	NR	NR	NR	NR	NR	NR	NR	NR	NR
					**Radiology residents (*n* = 6, 1—3 years of experience)—Aided (Center 1)**	NR		**0.883**	NR	NR	NR	NR	NR	NR	NR	NR	NR	NR
					Board-certified radiologists (*n* = 5, 7—8 years of experience)—Unaided (Center 1)	NR		0.74	NR	NR	NR	NR	NR	NR	NR	NR	NR	NR
					**Board-certified radiologists (*n* = 5, 7—8 years of experience)—Aided (Center 1)**	NR		**0.89**	NR	NR	NR	NR	NR	NR	NR	NR	NR	NR
					Thoracic radiologists (*n* = 4, 9—26 years of experience)—Unaided (Center 1)	NR		0.89	NR	NR	NR	NR	NR	NR	NR	NR	NR	NR
					**Thoracic radiologists (*n* = 4, 9—26 years of experience)—Aided (Center 1)**	NR		**0.91**	NR	NR	NR	NR	NR	NR	NR	NR	NR	NR
		182 (67♀, 115♂)	182 CECTs	Detection	Model (CNN, CT-based)—2/4 institutions (South Korea)	0.924		NR	NR	82	NR	NR	NR	30	NR	NR	NR	NR
				Diagnosis (binary, malignant vs. non-malignant)	**Model (CNN, CT-based)—2/4 institutions (South Korea)**	NR		**0.99**	NR	91.1	98	99.1	NR	NR	0.949	NR	NR	NR
		181 (81♀, 100♂)	181 CECTs	Detection	Model (CNN, CT-based)—3/4 institutions (South Korea)	0.831		NR	NR	69.6	NR	NR	NR	2	NR	NR	NR	NR
				Diagnosis (binary, malignant vs. non-malignant)	**Model (CNN, CT-based)—3/4 institutions (South Korea)**	NR		**0.94**	NR	71.2	100	100	NR	NR	0.832	NR	NR	NR
		149 (65♀, 84♂)	149 CECTs	Detection	**Model (CNN, CT-based)—4/4 institutions (USA)**	0.88		**NR**	NR	75	NR	NR	NR	25	NR	NR	NR	NR
				Diagnosis (binary, malignant vs. non-malignant)	**Model (CNN, CT-based)—4/4 institutions (USA)**	NR		**0.96**	NR	88	93	95.1	NR	NR	0.912	NR	NR	NR
	She [93]	1182 (642♀, 550♂)	NA	Cancer-specific survival	**Model (MLP, text-based)**	**NR**	NR		NR	NR	NR	NR	NR	NR	NR	**0.742**	NR	NR
					TNM-8	NR	NR		NR	NR	NR	NR	NR	NR	NR	0.706	NR	NR
	Yang [95]	172 (39♀, 133♂)	NA	Survival risk stratification	**Model (N-MTLR, text-based)**	**NR**	**0.7439**		NR	NR	NR	NR	NR	NR	NR	0.665	NR	NR
					TNM-8	NR	0.561		NR	NR	NR	NR	NR	NR	NR	NR	NR	NR
	Wang [98]	61 (39♀, 22♂)	63 CTs	Diagnosis (binary, malignant vs. benign)	Model (CNN, CT images)	NR	0.83		75	77	**69**	89	**47**	NR	NR	NR	NR	NR
					Radiologist 1 (5 years of experience)	NR	NR		81	87	44	**91**	33	NR	NR	NR	NR	NR
					Radiologist 2 (10 years of experience)	NR	NR		**83**	**90**	33	90	33	NR	NR	NR	NR	NR
**Mesothe-lium**	Naso [100]	39	39 WSIs (H&E slides magnified 40 times)	Diagnosis (binary, malignant vs. benign)	Model (CNN + RNN, image-based)—External validation	NR	0.989		NR	NR	NR	NR	NR	NR	NR	NR	NR	NR
		40*	40 WSIs (H&E slides magnified 40 times)		**Model (CNN + RNN, image-based)—Referral test set**	**NR**	NR		**92.5**	85.7	**100**	NR	NR	NR	NR	NR	NR	NR
					Pathologists (*n* = 3)—Referral test set	NR	NR		91.7	**87.3**	96.5	NR	NR	NR	NR	NR	NR	NR
**Naso-pharynx**	Li [101]	355 (124♀, 231♂)	1430 white-light endoscopic images	Diagnosis (binary, malignant vs. benign)	**CNN (white-light endoscopic images)**	**NR**	**0.93**		**88**	90.2	**85.5**	**86.9**	**89.2**	NR	NR	NR	NR	**40**
					Expert Endoscopists (*n* = 3)	NR	NR		80.5	89.5	70.8	76.6	86.4	NR	NR	NR	NR	6600
					Resident Endoscopists (*n* = 8)	NR	NR		72.8	88.8	55.5	69.5	83.2	NR	NR	NR	NR	5958
					Interns (*n* = 3)	NR	NR		66.5	**92.2**	38.9	62.3	82.2	NR	NR	NR	NR	6402

*AUC* Area Under the ROC Curve, *ACC* Accuracy, *CECT* Contrast-Enhanced CT, *CNN* Convolutional Neural Network, *CT* Computed Tomography, *FPR* False Positive Rate, *JAFROC* Jackknife Alternative Free-Response ROC, *MLP* Multi-layer Perceptron, *NECT* Non-Constrast Enhanced CT, *N-MTLR* Neural Multitask Logistic Regression, *NPV* Negative Predictive Value, *NR* Not Reported, *PPV* Positive Predictive Value, *PT* Processing Time, *ROC* Receiver Operating Characteristic, *SEN* Sensitivity, *SPEC* Specificity, *TNM* Tumor, Node, and Metastasis staging system, *TNM-8* Eighth edition of the TNM staging system, *XGBoost* Extreme Gradient Boosting

##### Lung Cancer

Ten approaches were developed for lung cancer assessments. The first document describes a validation study of a CNN-based tool (DenseNet) designed to predict the malignancy of pulmonary nodules [96]. The model was previously trained with the NLST dataset and was now externally validated in 3 UK centers with different CT scanners (1 397 CECTs and NECTs, 1 187 patients of unknown gender ratio). The authors also evaluated its clinical utility by comparing it to the Brock Model. Although slightly less specific than the Brock model, the detection algorithm developed by the authors had a higher AUC and sensitivity. Despite having undergone international validation, prospective studies in ethnically diverse populations are still amiss.

The second paper involved developing and validating a model to predict the malignancy of multiple pulmonary nodules from CT scans and eleven clinical variables [91]. The study analyzed data from various medical centers. Ten ML methods were compared to identify the best malignancy predictor: AdaBoost, DT, Logistic Regression, Linear SVM, Radial Basis Function Kernel SVM, NB, kNN, Neural Net, Quadratic Discriminant Analysis, RF, and XGBoost. The best-performing model – XGBoost – was tested on three datasets. The first was retrospective, compiled from 6 institutions (five from China and one from South Korea), used for primary external validation (220 patients, 583 CT scans), and compared against four well-established models: Brock, Mayo, PKU, and VA. The second retrospective dataset was used for generalizability, containing patients from a Chinese institution with solitary pulmonary nodules (195 patients and images, 110 women, 85 men), whose results were also compared against the four just-mentioned models. The third and last dataset included data from 4 Chinese centers and was collected prospectively for secondary validation and comparisons against clinicians (200 CTs, 78 patients, 51 women, 27 men). This comparison involved three thoracic surgeons and one radiologist, who achieved an average sensitivity of 0.651 and specificity of 0.679. The model significantly outperformed this average and each clinician’s AUC, as well as in all comparisons against the routinely used models. In addition, SHAP was used to identify the most predictive nodule characteristics, finding that the model's most predictive features were nodule size, type, count, border, patient age, spiculation, lobulation, emphysema, nodule location, and distribution. Nonetheless, besides not reporting individual clinician sensitivity and specificity in the prospective cohort, the drawbacks of this study include only assessing typical high-risk patients and the lack of validation with different ethnicities.

The work in [89] involved a CNN-based model for predicting the presence of visceral pleural invasion in patients with early-stage lung cancer. The deep learning model was trained using a dataset of CT scans from 676 patients and externally validated on a temporally different cohort from the same South Korean institution (141 CTs from 84 women and 57 men). Besides using Grad-CAM to evidence its decisions, this CNN can adapt its sensitivity and specificity to meet the clinical needs of individual patients and clinicians. The model achieved a performance level comparable to three expert radiologists but did not surpass it except in PPV. Besides, these are results from a monoinstitutional retrospective study where geographical validation was not performed. In addition to using a small number of patients, data was also imbalanced, and the model was not fully automated (required manual tumor annotations).

The fourth article concerns developing an EfficientNetV2-based CNN system to predict the survival benefit of tyrosine kinase inhibitors (TKIs) and immune checkpoint inhibitors (ICIs) in patients with stage IV non-small cell lung cancer [97]. The model was developed with accessible pre-therapy CT images from five centers and externally validated on a monoinstitutional dataset from a national dataset (China, 92 CTs from 92 patients). The authors also compared radiologists' and oncologists' (three each, 2, 5, and 10 years of experience) performance with and without ESBP. The results showed that, while assisted by the system, all radiologists improved their diagnostic accuracy, sensibility, specificity, PPV, and NPV (except for the trainee oncologist, who achieved better sensitivity without the model). However, prospective studies in ethnically rich cohorts are still necessary to implement this tool in clinical practice.

The fifth study aimed at finding optimal predictors of two-year recurrence, recurrence-free survival, and overall survival after curative-intent radiotherapy for non-small cell lung cancer [92]. Ten text-based ML models were trained on 498 patients and compared: ANN, Linear and Non-linear SVM, Generalized Linear Model, kNN, RF, MDA, Partial Least Squares, NB, and XGBoost. The best-performing models were as follows: (i) an ensemble of kNN, NB, and RF for recurrence classification; (ii) kNN for recurrence-free survival prediction; and (iii) a combination of XGBoost, ANN, and MDA for overall survival. The three optimal predictors were externally validated using routinely collected data from 5 UK institutions (159 seniors, 71 women, 88 men) and compared against TNM-8 and WHO performance status. The recurrence and overall survival models outperformed both routinely used systems, but these tools surpassed the recurrence-free survival predictor’s performance. Moreover, this study was retrospective and had a small sample size with missing data.

The sixth study was designed to identify high-risk smokers to predict long-term lung cancer incidence (12 years) [94]. In this paper, the authors developed a convolutional neural inception V4 network based on low-dose chest CT images, age, sex, and current versus former smoking statuses. The CNN was trained using patients from the PLCO trial and externally validated on data from the NLST randomized controlled trial (2456 women and 3037 men from 33 USA institutions). The model was also compared against PLCOm2012 to evaluate clinical utility, having exceeded its performance for all assessed metrics (AUC, sensitivity, specificity, PPV, and NPV). However, this study was retrospective, lacked ethnic diversity, and was not evaluated in a clinically realistic scenario. Additionally, information from symptomatic patients was unavailable due to using data from a screening trial.

In the seventh article, a CNN-based model was developed for the automated detection and diagnosis of malignant pulmonary nodules on CECT scans [90]. The algorithm was externally validated on four separate datasets with ethnic differences (three from South Korea and one from the USA, amounting to 693 patients and CTs). Furthermore, the diagnostic performance of 18 physicians (from non-radiologists to radiologists with 26 years of experience) was compared while assisted and not assisted by the algorithm for one dataset. The model achieved an excellent performance in the four tested datasets, outperforming all clinicians, and the professionals’ accuracy increased while aided by the model for all tested groups. Nonetheless, the model was undertrained for small nodules (< 1 cm) and trained only for malignant nodule detection for one type of CT (posterior-anterior projections), and the study was retrospective and not representative of a real-world clinical setting.

The eighth algorithm consisted of a multilayer perceptron (Feed-Forward Neural Network) paired with a Cox proportional hazards model to predict cancer-specific survival for non-small cell lung cancer [93]. The text-based model was trained using the SEER database and externally validated on patients from a Chinese tertiary pulmonary hospital (642 women, 540 men). It was compared against TNM-8, having outperformed it with statistical significance. Although tested with real-world clinical data, prospective multi-institutional studies are needed before the deep learning model can be used in clinical practice.

The ninth article described developing, validating, and comparing three CNN models to differentiate between benign and malignant pulmonary ground-glass nodules (GGNs) [98]. The first CNN only used CT images. The second CNN used clinical data: age, sex, and smoking history. The third was a fusion model combining CTs and clinical features, achieving the best performance. This model was temporally and geographically validated with 63 CT scans from 61 patients (39 women, 22 men). Its classification performance was compared against two radiologists (5 and 10 years of experience) for clinical utility assessment. Despite performing satisfactorily in external validation, the model was surpassed by both clinicians in accuracy, sensitivity, and NPV, only producing higher results for specificity and NPV. Furthermore, this study was retrospective, and validation was neither international nor evaluated in a correct clinical setting.

In the tenth and final paper, a Neural Multitask Logistic Regression (N-MTLR) network was developed for survival risk stratification for stage III non-small cell lung cancer [95]. The text-based deep learning system was trained on 16 613 patients from the SEER database and externally validated on subjects from a Chinese institution (172 patients, 39 women, 133 men). The results in the external dataset showed that the DSNN could predict survival outcomes more accurately than TNM-8 (AUC of 0.7439 vs. 0.561). The study results suggest that the deep learning system could be used for personalized treatment planning and stratification for patients with stage III non-small cell lung cancer. However, prospective studies in multi-institutional datasets are still required.

##### Laryngeal, Mesothelial and Nasopharyngeal Cancers

Three models were developed to assess tumors of other elements of the respiratory system. In [99], the authors trained a CNN (GoogLeNet Inception v3 network) with 13 721 raw endoscopic laryngeal images – including laryngeal cancer (LCA), precancerous laryngeal lesions (PRELCA), benign laryngeal tumors (BLT), and healthy tissue – from three Chinese institutions (1 816 patients). External validation was performed on 1 176 white-light endoscopic images from two additional institutions in the same country (392 patients), testing the model for binary classification – urgent (LCA and PRELCA) or non-urgent (BLT and healthy) – and between the four conditions. Predictions for both classification types were compared against three endoscopists (3, 3 to 10, and 10 to 20 years of experience). In two-way classification, the algorithm was less accurate than one endoscopist and less sensitive than two but outperformed all clinicians in four-way diagnostic accuracy. Still, this accuracy was relatively low (less than 80%), the study was retrospective, and all tested laryngoscopic images were obtained by the same type of standard endoscopes.

Cancers of the mesothelium were approached in a single retrospective multi-center study [100]. The paper uses DL to distinguish between two types of mesothelial cell proliferations: sarcomatoid malignant mesotheliomas (SMM) and benign spindle cell mesothelial proliferations (BSCMP). SMMs and BSCMPs are difficult to distinguish using traditional histopathological methods, resulting in misdiagnoses. The authors propose a new strategy—SpindleMesoNET—that uses an ensemble of a CNN and an RNN to analyze WSIs of H&E-stained mesothelial slides magnified 40 times. The model was trained on a Canadian dataset, externally validated on 39 images from 39 patients from a Chinese center, and compared against the diagnostic performance of three pathologists on a referral test set (40 WSIs from 40 patients). The accuracy and specificity of SpindleMesoNET on the referral set cases (92.5% and 100%, respectively) exceeded that of the three pathologists on the same slide set (91.7% and 96.5%). However, the pathologists were more sensitive than the diagnostic model (87.3% vs. 85.3%). In addition, the study had a minimal sample size, and only AUC was reported for the external validation dataset (0.989), which, although considerably high, is insufficient to assess the model’s effectiveness.

The last study entailed developing and validating a CNN-based model to differentiate malignant carcinoma from benign nasopharyngeal lesions using white-light endoscopic images [101]. Malignant conditions included lymphoma, rhabdomyosarcoma, olfactory neuroblastoma, malignant melanoma, and plasmacytoma. Benign subtypes encompassed precancerous or atypical hyperplasia, fibroangioma, leiomyoma, meningioma, minor salivary gland tumor, fungal infection, tuberculosis, chronic inflammation, adenoids or lymphoid hyperplasia, nasopharyngeal cyst, and foreign body. The model was trained on 27 536 images collected retrospectively (7 951 subjects) and temporally (prospectively) externally validated with 1 430 images (from 355 patients) from the same Chinese institution. Diagnostic performance was compared against 14 endoscopists: (i) three experts with more than five years of experience; (ii) eight residents with one year of experience; and (iii) interns with less than three months of experience. Except for the interns’ sensitivity, the model’s diagnostic performance surpassed the endoscopists in all tested metrics. However, data were collected from a single tertiary institution, and more malignancies should be included.

Although not developed for the same cancer type, the two cancer detection studies for the larynx [99] and nasopharynx [99, 101] are comparable due to using white-light endoscopic images. Both used CNNs and involved more than 300 patients and 1000 images, but the optimal diagnostic performance – although less sensitive (72% vs. 90.2% in [101]) – was achieved for the GoogLeNet Inception v3 network CNN [99] with an AUC of 0.953, an accuracy of 89.7%, and a specificity of 94.8%, enhancing the value of pre-training CNNs.

#### Skeletal system

Four studies using different imaging techniques were designed to diagnose bone cancers, producing an average AUC of 0.88 (Table 13). The first two radiomics-based models were developed for the binary classification of atypical cartilaginous tumors (ACT) and appendicular chondrosarcomas (CS) [103, 104].

**Table 13:** External validation and clinical utility performance for models evaluating primary cancers of the bones

Author	Patients	Images	Outcome	Comparator	AUC	ACC	SEN	SPEC	PPV	NPV	F1-Score	Brier Score
Gitto [104]	36 (23♀, 13♂)	36 PET-CT images	Diagnosis (binary, ACT vs. CS)	Model (LogitBoost, PET-CT-based)	0.784	75	75	NR	76.2	NR	0.751	0.25
				Radiologist (*n* = 1)	NR	**81**	NR	NR	NR	NR	NR	NR
Gitto [103]	65 (34♀, 31♂)	65 T1-weighted MRI scans	Diagnosis (binary, ACT vs. CS)	Model (ExtraTrees, MRI-based)	0.94	92	92	NR	92	NR	0.92	0.09
				Radiologist (*n* = 1)	NR	97.5	NR	NR	NR	NR	NR	NR
He [102]	291 (175♀, 116♂)	639 X-rays	Diagnosis (binary)	Model (CNN, X-ray-based): benign vs. non-benign	0.877	NR	NR	NR	NR	NR	NR	NR
				Model (CNN, X-ray-based): malignant vs. non-malignant	0.916	NR	NR	NR	NR	NR	NR	NR
			Diagnosis (triple, benign vs. intermediate vs. malignant)	**Model (CNN, X-ray-based)**	**NR**	**73.4**	NR	NR	NR	NR	NR	NR
				Musculoskeletal subspecialist Radiologist 1 (25Y)	NR	69.3	NR	NR	NR	NR	NR	NR
				**Musculoskeletal subspecialist Radiologist 2 (23Y)**	**NR**	**73.4**	NR	NR	NR	NR	NR	NR
				Trainee Radiologist 1 (6Y)	NR	73.1	NR	NR	NR	NR	NR	NR
				Trainee Radiologist 2 (1Y)	NR	67.9	NR	NR	NR	NR	NR	NR
				Trainee Radiologist 3 (7Y)	NR	63.4	NR	NR	NR	NR	NR	NR
Von Shacky [105]	96 (40♀, 56♂)	96 X-rays	Diagnosis (binary, malignant vs. benign, 16 recognized conditions)	Model (ANN, X-ray and text-based)	**0.9**	75	**90**	68	57	95	NR	NR
				Radiologist Residents (*n* = 2)	NR	68.5	48	76.5	NR	NR	NR	NR
				Expert Radiologists (*n* = 2)	NR	**83.5**	85.5	**83.5**	NR	NR	NR	NR

*ACC* Accuracy, *ACT* Atypical Cartilaginous Tumor, *AUC* Area Under the ROC Curve, *CNN* Convolutional Neural Network, *CS* Chondrosarcoma, *ExtraTrees* Extremely Randomized Trees. *MRI* Magnetic Resonance Imaging, *PET-CT* Positron Emission Tomography – Computed Tomography, *RF* Random Forest, *ROC* Receiver Operating Characteristic, *NPV* Negative Predictive Value, *NR* Not Reported, *SEN* Sensitivity, *SPEC* Specificity, *Y* Years

In [104], a LogitBoost algorithm was temporally and geographically validated on 36 PET-CT scans from 23 women and 13 men. Besides externally validating their method, the authors evaluated clinical utility by comparing its diagnostic performance against a radiologist. The model performed satisfactorily in all calculated metrics (AUC, accuracy, sensitivity, PPV, and F1-score), but its accuracy was lower than the radiologist. In addition, only non-contrast PET-CT scans were included in the analyses.

In the following year, research performed by the same first author evaluated bone tumor diagnosis from MRI scans [103]. Radiomic features were extracted from T1-weighted MRI scans, and an ExtraTrees algorithm was trained to classify the tumors. On an external validation dataset of 65 images (34 women, 31 men), the model achieved a PPV, sensitivity, and F1-score of 92%, 98%, and 0.95 in classifying ACTs, while 94%, 80%, and 86% for the classification of grade II CS of long bones, respectively (weighted average is presented in Table 13). The model's classification performance was compared against an experienced radiologist (with 35 years of experience) to assess clinical utility, finding that it could not match the radiologist's performance. Using SHAP, it was also found that certain radiomic features, such as the mean and standard deviation of gradient magnitude and entropy, significantly differed between the two tumor types. Drawbacks include the study’s retrospective nature, using only one type of MRI, and over-representing appendicular chondrosarcomas compared to cartilaginous tumors in the study population.

The second set of papers used neural networks to differentiate benign from malignant bone tumors from X-ray images [102, 105]. On the one hand, in [102], a CNN (EfficientNet-B0) was developed on a dataset of 2899 radiographic images from 1356 patients with primary bone tumors from 5 institutions (3 for training, 2 for validation), including benign (1523 images, 679 patients), intermediate (635 images, 317 patients), and malignant (741 images, 360 patients) growths. The CNN model was developed for binary (benign versus not benign and malignant versus not malignant) and three-way (benign versus intermediate versus malignant) tumor classification. The authors also compared the model’s triple-way classification performance against two musculoskeletal subspecialists with 25 and 23 years of experience and three junior radiologists with 6, 1, and 7 years of experience. The deep learning algorithm had similar accuracy to the subspecialists and better performance than junior radiologists. However, only a modest number of patients was used for validation (639 X-rays from 291 patients), tumor classes were unbalanced (smaller number of benign bone tumors compared to intermediate and malignant), and the pipeline was not fully automated.

In contrast, other authors resorted to a non-deep ANN that uses radiomic features extracted from X-ray images and demographic data to classify and differentiate malignant and benign bone tumors [105]. The ANN was developed on 880 patients with the following conditions: (i) malignant tumors: chondrosarcoma, osteosarcoma, Ewing’s sarcoma, plasma cell myeloma, non-Hodgkin lymphoma B cell, and chordoma; (ii) benign subtypes: osteochondroma, enchondroma, chondroblastoma, osteoid osteoma, giant cell tumor, non-ossifying fibroma, haemangioma, aneurysmal bone cyst, simple bone cyst, fibrous dysplasia. The method was externally validated on 96 patients from a different institution, and performance was compared against four radiologists (two residents and two specialized). The model was more sensitive than both radiologist groups but was outperformed by the specialized radiologists in accuracy and specificity. In addition, the model requires manual segmentations and can only distinguish between benign and malignant tumors and not specific subtypes.

### Metastases (Secondary Tumors)

As shown in Table 14, five studies entailed the assessment of metastatic cancer, that is, secondary tumors spread from different tissues. From these, three focused on cancer spread to organs [106–108], while two evaluated metastasized nodes.

**Table 14:** External validation and clinical utility performance for models evaluating metastatic cancers

First Author	Metastases	Primary Cancer	Patients	Images	Outcome	Comparator	AUAF-ROC	AUC	ACC	SEN	SPEC	PPV	NPV	FPR	F1-Score
Ji [107]	Bone	Kidney	963 (323♀, 640♂)	NA	Risk (diagnosis)	Model (XBoost, text-based)	NR	**0.98**	95	95	NR	95	NR	NR	0.94
						**TNM-7**	NR	0.93	**96**	**96**	NR	**96**	NR	NR	**0.96**
					Overall survival (3 years)	**Model (XBoost, text-based)**	NR	**0.91**	**92**	**92**	NR	**94**	NR	NR	**0.93**
						TNM-7	NR	0.89	91	91	NR	82	NR	NR	0.86
Kim [108]	Liver	Colorectal	85 (31♀, 54♂)	250 CECTs	Detection	Model (CNN, image-based)	0.631	NR	NR	NR	NR	NR	NR	NR	NR
						Radiologists (*n* = 3, 2, 3, and 20 years of experience)	0.723	NR	NR	NR	NR	NR	NR	NR	NR
						**Second-year Radiology Residents (*n* = 3)**	**0.66**	NR	NR	NR	NR	NR	NR	NR	NR
					Diagnosis (classification)	Model (CNN, image-based)	NR	NR	NR	**81.82**	NR	NR	NR	1.33	NR
						Radiologists (*n* = 3, 2, 3, and 20 years of experience)	NR	NR	NR	80.81	NR	NR	NR	**0.357**	NR
						Second-year Radiology Residents (*n* = 3)	NR	NR	NR	79.46	NR	NR	NR	0.667	NR
Lee [109]	Cervical Lymph Nodes	Thyroid	698	3838 CECTs	Diagnosis (classification)	Model (CNN, image-based)	NR	0.884	82.8	80.2	83	83	80.2	NR	NR
						Experienced radiologist (*n* = 1, 14 years of experience)—Unaided	NR	NR	91.7	94.7	90.2	66.7	98.9	NR	NR
						Experienced radiologist (*n* = 1, 14 years of experience)—Aided	NR	NR	93	97.4	90.2	64.9	99.5	NR	NR
						Trainee radiologists (*n* = 6, 1–3 years of experience)—Unaided	NR	NR	75	91.2	72	51.7	75	NR	NR
						Trainee radiologists (*n* = 6, 1–3 years of experience)—Aided	NR	NR	76.2	90.8	73.4	44	97.8	NR	NR
Zachariah [106]	Solid Metastases	Aggregate	2041 (770♀, 1271♂, 3099 encounters)	NA	Three-month mortality	**Model (GBDT, text-based)**	NR	**0.812**	NR	29.5	**96.7**	**60**	**89.1**	NR	NR
						Oncologists (*n* = 74)	NR	0.598	NR	**29.7**	90	34.8	87.7	NR	NR
		Breast	482	NA	Three-month mortality	Model (GBDT, text-based)	NR	0.873	NR	36.2	NR	**53.2**	NR	NR	NR
						Oncologists (*n* = 74)	NR	NR	NR	**37.5**	NR	36.5	NR	NR	NR
		Gastrointestinal	629	NA	Three-month mortality	**Model (GBDT, text-based)**	NR	**0.81**	NR	**52.6**	NR	**43.8**	NR	NR	NR
						Oncologists (*n* = 74)	NR	NR	NR	52.1	NR	32.5	NR	NR	NR
		Genitourinary	280	NA	Three-month mortality	**Model (GBDT, text-based)**	NR	0.85	NR	19	NR	**42.1**	NR	NR	NR
						Oncologists (*n* = 74)	NR	NR	NR	**20**	NR	32.1	NR	NR	NR
		Lung	378	NA	Three-month mortality	**Model (GBDT, text-based)**	NR	**0.777**	NR	**10.1**	NR	**63.6**	NR	NR	NR
						Oncologists (*n* = 74)	NR	NR	NR	9.6	NR	46.7	NR	NR	NR
		Rare tumors	272	NA	Three-month mortality	Model (GBDT, text-based)	NR	0.764	NR	22.2	NR	**60.9**	NR	NR	NR
						Oncologists (*n* = 74)	NR	NR	NR	**23.1**	NR	38.5	NR	NR	NR
		All (No treatment changes)	NR	NA	Three-month mortality	**Model (GBDT, text-based)**	NR	**0.801**	NR	**27.3**	NR	**49**	NR	NR	NR
						Oncologists (*n* = 74)	NR	NR	NR	26.8	NR	33.1	NR	NR	NR
		All (With treatment changes)	NR	NA	Three-month mortality	**Model (GBDT, text-based)**	NR	**0.824**	**NR**	**31.7**	NR	**64.2**	NR	NR	NR
						Oncologists (*n* = 74)	NR	NR	NR	31.4	NR	35.8	NR	NR	NR
Zhang [110]	Sentinel lymph nodes	Breast	50♀	50 ultrasounds	Risk (diagnosis)	**Model (XBoost, image-based)**	NR	**0.916**	**84.6**	**87**	**86.2**	NR	NR	NR	**0.826**
						Radiologist (*n* = 1)	NR	0.758	NR	NR	NR	NR	NR	NR	NR

For clinicians with years of experience, metrics are presented individually (if reported). For physicians without this information (or readers without age matching), results are grouped by rank (if available) or all together

*ACC* Accuracy, *AUAFROC* Area Under the Alternative Free-response ROC curve, *AUC* Area Under the ROC Curve, *CECT* Contrast-Enhanced CT, *CNN* Convolutional Neural Network, *CT* Computed Tomography, *EUS* Endoscopic Ultrasonography, *GBDT* Gradient Boosted Decision Tree, *NPV* Negative Predictive Value, *NA* Not Applicable, *NR* Not Reported, *PPV* Positive Predictive Value, *ROC* Receiver Operating Characteristic, *TNM* Tumor, Node, and Metastasis staging system, *TNM-7* Seventh edition of the TNM staging system, *TNM-8* Eighth edition of the TNM staging system, *XGBoost* Extreme Gradient Boosting

#### Organ metastases

In [107], models were created to predict the risk of bone metastasis and prognosis (three-year overall survival) for kidney cancer patients. To achieve optimal performance, the researchers developed and compared eight ML models: DTs, RFs, MLPs, Logistic Regression, Naïve Bayes BS classifier, XGBoost, SVMs, and kNN. The text-based models were trained with 71 414 patients from the SEER database (USA) and externally validated with 963 patients from a Chinese institution (323 women, 640 men). The results showed that their XGBoost-based models had the best accuracy in predicting bone metastasis risk and prognosis. The risk prediction model (diagnosis) outperformed TNM-7 only regarding AUC (0.98 vs. 0.93), while the prognostic model exceeded TNM-7’s predictions for all tested metrics (AUC, accuracy, sensitivity, PPV, and F1-score). Using SHAP analysis, the authors also unveiled that the key factors influencing these outcomes were age, sex, and tumor characteristics. Although trained on ethnically different patients, these models were only validated on Asian subjects and not compared against clinicians, so further studies are required to establish clinical validity and utility.

The second paper explores the effectiveness of a deep learning-based algorithm (CNN) in detecting and classifying liver metastases from colorectal cancer using CT scans [108]. In this South Korean monoinstitutional study, 502 patients were used for training, and temporally different patients (40 with 99 metastatic lesions, 45 without metastases) were used for validation. The algorithm's detection and classification performance was compared to three radiologists (with 2, 3, and 20 years of experience in liver imaging) and three second-year radiology residents. Although showing a higher diagnostic sensitivity than both types of clinicians, the six radiologists outperformed the model in AUAFROC (detection) and false positives per patient (FPP, classification). In addition, the CT scans had been captured eight years before the analyses.

The third study was conducted in a clinically realistic scenario, and the model has been implemented in practice [106]. The model was designed to predict 3-month mortality in patients with solid metastatic tumors for several types of cancer (breast, gastrointestinal, genitourinary, lung, rare) and treatment alterations in an outpatient setting. The authors trained a Gradient-Boosted Trees Binary Classifier with observations from 28 484 deceased and alive patients and 493 features from demographic characteristics, laboratory test results, flowsheets, and diagnoses. The model was silently deployed in the patients’ EHRs for 20 months to compare its predictions against 74 oncologists. This prospective temporal validation study involved 3099 encounters from 2041 ethnically diverse patients. The model outperformed oncologists in all metrics for aggregate (general, with and without treatment alterations), gastrointestinal, genitourinary, and lung cancers but was less sensitive than the professionals for rare and breast metastatic tumors. Although currently available in medical practice, the authors note that further research is needed to validate whether using the model improves prognostic confidence and patient engagement.

#### Node metastases

Two models were developed to diagnose node metastases. In [109], the authors aimed to classify cervical lymph node metastasis from thyroid cancer using CT scans [109]. The researchers had previously developed a CNN (Xception architecture) trained on a 787 axial preoperative CT scans dataset. This study validated the systems' performance on 3 838 images from 698 patients (unknown female-male ratio) and used Grad-CAM to explain the model’s reasoning. The researchers also evaluated the clinical utility of the model by comparing seven radiologists’ performance (one expert, six trainees) with and without its assistance. While aided by the system, the expert’s accuracy, sensitivity, specificity, PPV, and NPV were all found to increase, while only accuracy, specificity, and NPV improved for the trainees. This study was retrospective and conducted in a single institution, and the results obtained were not satisfying enough to justify clinical implementation.

The second and last document describes developing an ultrasound-based ML model to assess the risk of sentinel lymph node metastasis (SLNM) in breast cancer patients [110]. First, the authors compared ten algorithms to achieve an optimal model: SVM, RF, LDA, Logistic Regression, NB, kNN, MLP, Long Short-Term Memory, and CNN. The best algorithm (XGBoost) was then integrated into a clinical model, and SHAP was used to analyze its diagnostic performance. XGBoost was trained with 902 patients, and external validation consisted of 50 temporally separate women. The authors also compared their tool with a radiologist’s diagnostic evaluations (unknown years of experience). The results showed that the ML model could predict the risk of SLNM in breast cancer patients based on ultrasound image features with high accuracy (84.6%), having outperformed the radiologist. In addition, SHAP analysis deemed suspicious lymph nodes, microcalcifications, spiculation at the edge of the lesion, and distorted tissue structure around the lesion as the model’s most significant features. Nonetheless, this research was retrospective and used a minimal number of patients from a single institution with limited pathological types of breast cancer.

## Discussion

We conducted a scoping review to gather externally validated ML algorithms developed for patient care in oncology whose clinical utility has also been assessed. Given the rapidly evolving nature of the field and the potential for novel approaches and emerging research, and unlike previous reviews [23, 37, 39, 41, 124], a deliberate decision was made not to restrict the search strategy or outcomes stringently. The objective was to adopt a comprehensive and inclusive process to capture a diverse range of literature that could potentially contribute to our understanding of externally validated machine learning algorithms in the context of oncology practice. This approach allowed for exploring various cancer variants, clinical outcomes, validation methodologies, and clinical utility assessments without preconceived limitations that might have excluded relevant studies.

### Principal findings

The findings from this scoping review reveal several critical insights into the landscape of ML and DL applications in cancer-patient-related decision-making. A notable prominent trend is their increasing recognition and interest. The dominance of papers focused on patients and medical issues (versus computational journals, Fig. 2A) highlights this growing enthusiasm and a strong emphasis on tackling clinical challenges and reflects a paradigmatic transition from theoretical and computational considerations toward practical, patient-oriented solutions. This is underscored by the significant rise in relevant sources after 2018, particularly in 2020, 2021, and 2022 (Fig. 2B). However, it's crucial to note that many papers were excluded due to insufficient external validation and clinical utility assessment (Fig. 1), showing that the model development and testing methodology still lack standardization, which agrees with the literature [23, 37]. These observations collectively emphasize the evolution and maturation of the field, yet they also serve as a call to action for enhancing the methodological rigor of research endeavors.

Concerning the first research question, we found that CNNs have risen to prominence and are now the backbone of most research initiatives (33/56 papers). Random Forests and XGBoost, while less common, still played significant roles, featuring in 7/56 and 6/56 of the studies, respectively, adding diversity to the oncology decision-making landscape. While lung cancer and digestive system assessments were the primary focus, these algorithms demonstrated versatile applicability across various cancer types. Moreover, the emphasis on image-based analyses reflects the potential of ML in augmenting the accuracy of diagnostic processes. However, the limited attention to risk stratification and pharmacotherapy research is a notable caveat. Likewise, the underutilization of radiomics in image studies indicates a missed opportunity. Incorporating radiomics can provide a wealth of information about tumor characteristics and heterogeneity, enriching our understanding and predictive capabilities in oncology. These are areas where ML can make significant contributions to the field, highlighting future directions for research and untapped potential for exploring alternative methodologies.

Indeed, methodological considerations highlight several areas that demand attention. The simultaneous development and validation of models in most papers could potentially introduce partiality [38]. Further, the limited sample sizes in many studies, with the majority involving fewer than 200 patients, raise concerns about the generalizability and robustness of these models [38, 41]. Equally, except for three prospective studies and four pieces of research encompassing both retrospective and prospective datasets, the selected papers were mainly retrospective (49/56), a less rigorous design potentially lowering data quality and compromising reliability [125]. Nonetheless, in contrast to previous reviews [126], we witnessed a substantial increase in multi-institutional studies, marking a positive transformation in the landscape of oncological research. The shift towards collaborative efforts involving multiple centers brings diversity to the study populations, which is critical for generalizing findings to broader patient groups and instilling confidence in research outcomes. Collaborative research involving several institutions augments resources, expertise, and data access, offering a deeper understanding of research questions. However, the infrequent international validation and the paucity of data and code sharing in multi-institutional studies present substantial hurdles. These challenges obstruct the path to enhanced reproducibility and collaborative progress. Scientifically, they emphasize the importance of standardizing data-sharing practices and code accessibility to facilitate transparency, rigor, and cooperation in the field.

Besides, the disconnect between data used in research and real-world clinical scenarios is an essential finding. In a clinical environment, both text and image-based information are often simultaneously available, making it crucial for ML models to adapt to such real-world complexities. The prevalence of models designed for binary classification, while suitable for emergency settings, reveals a limitation. Clinical decision-making is a complex process that often involves navigating numerous potential diseases, each with unique characteristics, presentations, and treatment considerations. The overreliance on binary classification fails to capture this richness and underscores the need for more nuanced approaches.

Furthermore, the observation that only two models have been effectively implemented in clinical practice highlights the gap between research findings and practical implementation. This finding underscores the challenges in translating scientific progress into real-world healthcare contexts. It draws attention to the necessity of comprehensive validation, addressing regulatory considerations, and managing the integration of new technologies into existing clinical workflows [127–129]. Additionally, building trust in AI systems is a crucial scientific contribution. The employment of XAI models in 15 reviewed papers demonstrates a proactive effort to enhance transparency and accountability. XAI provides insights into the underlying features, variables, or patterns that contribute to the model's decision-making process, enabling clinicians to comprehend and validate outputs and allay their wariness [130, 131]. This multi-dimensional approach acknowledges the technical and human factors critical for AI's successful implementation in healthcare.

Regarding the second research question, two main comparators were used to evaluate clinical utility: clinicians and routine clinical scoring systems and tools, with only one study adopting both types of comparative analyses [91]. An important consideration is the presence of a wide inter- and intra-variability in the number of included clinicians. While 499 medical professionals were identified across the reviewed studies, it is crucial to note that the distribution was heavily skewed. Specifically, only six studies involved a substantial number of clinicians (twenty or more). At the same time, eleven included a moderate number (between five and eighteen), and most had a considerably smaller sample size (four or fewer clinicians, 24 studies).

Furthermore, the observed variability underscores the importance of reporting detailed clinician characteristics. Although the number of clinicians was reported in the studies, there was limited information regarding their specific backgrounds, years of experience, and areas of specialization. Of the 41 studies comparing models against clinicians, eleven did not report years of experience, and ten only reported rank. Clinician expertise and experience can significantly influence diagnostic accuracy and decision-making outcomes, so studies with limited physicians with unreported proficiency may be more susceptible to bias and not fully encompass the full spectrum of clinical decision-making [23]. Besides, none of the comparisons were carried out in randomized trials, which is the most accurate way of testing utility [42].

Clinical utility was assessed mainly by comparing model and clinician performance separately, intended to evaluate each entity’s capabilities independently and capture the variations in clinical decision-making among different individuals or groups. Although helpful in calculating inter- and intra-observer variability, this approach may overlook the interaction dynamics between AI and clinicians and not fully reflect the complexities and challenges of real-world clinical practice. Conversely, performance with and without AI assistance was evaluated in ten papers, which helps discern the unique contributions of AI in terms of augmenting clinician judgment, providing additional insights, or improving efficiency. In addition, sixteen studies benchmarked the clinical utility of machine learning models against twelve commonly used clinical tools. Although more prone to bias and less generalizable [38], this type of comparison provides a uniform reference point for evaluating performance, assessing the practical impact and potential improvements the new method offers over the current standard of care. There is also a clear need for more comprehensive and standardized research in clinical utility, fostering a more effective and seamless integration of AI into healthcare decision-making. Future studies should strive for a more inclusive representation of clinicians, prioritize randomized trials for robust validation, and aim for a thorough understanding of how AI can complement and enhance human expertise.

Answering the third research question involves the reported performance during both external validation and clinical utility assessment. The impressive performance of CNNs across various cancer types presents a vital scientific contribution. Their consistently high performance underscores their reputation as a powerful tool in patient-focused cancer research. Additionally, the strong performance of Gradient and Decision Tree-based algorithms in diverse cancer-related tasks reveals an underrepresented facet of ML research. This finding highlights an opportunity to explore and evaluate different ML approaches in oncology applications. The variability in reporting discrimination metrics and calibration metrics, while illuminating the diversity of evaluation methods, raises a critical concern. The lack of standardization hampers the reliability and accuracy of risk assessments [38, 132], emphasizing the need for consistency in reporting and metrics. In assessing clinical utility, the notable superiority of ML models over clinical tools marks a significant scientific advancement. These findings signify the potential for ML to enhance clinical decision-making processes significantly. However, they also reveal that ML models have not yet reached the same level of expertise as human clinicians in certain aspects, pointing to a collaborative approach where AI systems complement and support clinicians rather than replace them. This collaborative model could offer a path forward to augmenting healthcare capabilities.

Finally, six main research gaps were found throughout the review. First, although common cancers were extensively studied in adults, metastases, rare tumors, and different age groups were only investigated in five [106–109], three [78, 100, 106], and two [92, 102] papers, respectively. For example, an evident instance of the limited research focus on rare tumors can be observed in the absence of studies examining breast cancers in men. This paucity might be attributable to insufficient publicly available data, the high cost of collecting new data in bulk [133], and scarce interaction between medical centers. Second, most models were developed for diagnosis, outcome predictions, or risk stratification, while studies on optimal treatment and drug administration options are still lacking. Third, most studies were retrospective with small sample sizes, thus requiring further prospective validations in diverse patient populations to ensure generalizability. Fourth, none of the image-based studies addressed low-quality images; this is essential for real-world clinical applications, as not all images obtained in practice may be optimal. Fifth, no study assessed utility on patient outcomes, which is not only the ultimate goal but also required by insurance coverage [42] and crucial for determining actual clinical utility and effectiveness. Sixth and last, the absence of studies involving digital twins – even during abstract inspection – is worth mentioning. Further exploration of ML models with these virtual replica technologies could provide meaningful contributions to their application in clinical practice. Likewise, these gaps could be bridged by encouraging and implementing collaboration in healthcare, as merging – ideally, at an international level – information from several institutes would result in more comprehensive data, less bias from country-specific patients and treatment recommendations [129] and larger sample sizes, and consequently, in a higher generalization capacity, and even faster and more accurate diagnoses and treatment decisions.

This research stands out for its inclusivity, encompassing diverse patient populations, ML algorithms, and hospital settings, enhancing the applicability of its findings. Its contributions lie in systematically exploring external validation and clinical utility evaluation for ML algorithms, bridging the gap between AI researchers and medical professionals. Lastly, this work highlights the paramount significance of the synergy between AI researchers and medical practitioners. Interdisciplinary collaboration is foundational for promoting the adoption of AI technologies in healthcare and enhancing their scientific and clinical contributions. It ensures that research is translated into innovative, hands-on solutions that align with clinical needs and standards, disease management, and clinical decision-making and positively impact patient care.

### Study limitations

Despite the valuable insights gained from this study, it is essential to acknowledge its limitations. First, relevant studies might have been missed despite efforts to design a comprehensive search strategy and the inclusion of databases from different research fields. For example, sequencing, omics, and molecular biomarker discovery studies were excluded from this review. Notwithstanding their critical role in advancing personalized medicine, genomic, transcriptomic, and proteomic approaches still face obstacles to widespread clinical adoption due to their complexity, the specialized analytical skills required, the need for substantial adjustments in clinical workflows, and significant regulatory challenges [54, 134]. Given these constraints, this review emphasized machine learning algorithms immediately employable in clinical operations, ensuring research is relevant and actionable within healthcare settings. However, this selection reflects a limitation. While narrowing the focus to technologies with broader immediate applicability, not incorporating genetics and omics studies may have inadvertently excluded a subset of literature that explicitly investigates the interplay between genetic factors, treatment regimens, and therapeutic responses, offering a potential explanation for the absence of papers exploring drug and treatment responses and digital twin approaches.

Second, this review did not extensively cover the emerging challenges and opportunities of stringent data protection laws, notably the potential for synthetic data in research. While this exclusion aimed at evaluating model performance in genuine patient data, thereby accounting for the complexities and variabilities inherent in healthcare, synthetic data offers a promising avenue for navigating privacy concerns and enhancing dataset diversity [53]. Hence, its absence marks a limitation, reflecting areas beyond the immediate scope of this review yet critical for the future of ML applications in oncology.

Third, although the review revealed mostly positive results highlighting ML’s promise, the risk of publication bias cannot be discarded, as studies with positive or significant findings are more likely to be published than those with unfavorable or nonsignificant verdicts [135]. Similarly, the emphasis on SJR as a quality measure, while aiming to ensure the inclusion of high-impact research, acknowledges the potential oversight of specialized, significant studies that might not yet have achieved wide recognition but contribute meaningfully to the field. Furthermore, the selection process did not extend to evaluating the methodological quality or risk of bias within the included studies, potentially limiting the ability to characterize the overall strength of the evidence.

Fourth, a significant portion of the studies was retrospective, which increases susceptibility to selection bias and data quality concerns compared to prospective analyses, which may affect the conclusions' robustness [38, 136]. Small sample sizes and the lack of diversity within study populations further challenge the findings' generalizability, emphasizing the need for broader testing of machine learning models across diverse clinical contexts [137]. Additionally, external validation and clinical utility evaluations, often conducted within restricted scopes, may fail to represent the complexities encountered in real-world healthcare settings fully. This limitation suggests that the current body of research may not adequately reflect the potential challenges and applicability of machine learning solutions across the healthcare spectrum, restricting extrapolations.

Lastly, a notable methodological concern within the broader field, rather than this review alone, is the variability in performance metrics and a lack of standardized reporting practices across studies. This inconsistency hinders direct comparisons between research outcomes, urging standardized reporting guidelines to facilitate a more effective synthesis of research findings and accurately evaluate the progress of ML-based applications in oncology.

### Conclusions

Although facing challenges primarily tied to data availability and quality, machine learning models, with CNNs in the forefront, have consistently demonstrated substantial potential to revolutionize modern medicine and ultimately improve overall healthcare quality. These models have been especially impactful in lung, colorectal, gastric, bone, and breast cancers, offering a promising pathway for clinicians to make more accurate and personalized clinical decisions and reducing the need for invasive procedures. For instance, in lung cancer, CNNs have enhanced lesion detection, while in colorectal cancers, they have improved early neoplasm detection. Gastric cancer research has also benefited from AI’s ability to diagnose and predict treatment responses, offering new avenues for patient care. Similarly, using ML in breast cancer resulted in streamlined screening processes, and in bone cancer, these algorithms assisted in distinguishing benign from malignant lesions, allowing for earlier detection and treatment.

However, the path to fully leveraging ML in oncology highlights a pronounced need for model sensitivity and specificity refinement. Minimizing false positives and negatives is critical, particularly for cancers with intricate presentation patterns. Furthermore, our findings reveal a substantial gap in addressing less common and rare cancers, rising an imperative for the research community to extend its investigative efforts. By broadening the application of ML technologies to encompass these lesser-studied cancers, there is an opportunity to deepen their understanding and craft more inclusive and precise diagnostic and therapeutic approaches, thereby maximizing AI's impact across the full spectrum of oncological patient care.

Moving forward, we propose a comprehensive roadmap to guide the implementation of AI in clinical settings. The initial step involves standardized data collection and curation, emphasizing the creation of diverse, well-annotated datasets that accurately represent the complexity of real-world clinical scenarios. These datasets ensure consistency and reliability in model performance across various studies and healthcare institutions. The subsequent stages are centered around the rigorous development, external validation, and utility testing of AI models, placing a premium on homogeneity in discrimination and calibration metrics, robustness, and generalizability. The developed models should be integrated into clinical workflows in close collaboration with healthcare professionals, and ongoing training programs should be implemented to enhance their understanding of AI concepts. Simultaneously, establishing frameworks that address ethical governance, privacy protection, and regulatory compliance is crucial for navigating the legal and ethical considerations associated with AI implementation and promoting data sharing. Finally, fostering a culture of continuous improvement is essential, where AI models are regularly updated and refined based on feedback from clinicians, new data, and advancements in the field.

In conclusion, this review issues a resounding call for collective action from oncology stakeholders – clinicians, researchers, policymakers, and healthcare institutions. The findings reinforce the pressing need to fully embrace machine learning as an asset for patient-centered cancer research and decision-making. In this cooperative endeavor, it is imperative to ensure equitable access to high-quality data, engage in large-scale prospective studies, and foster international collaboration for the robust validation of AI models across diverse patient populations. Furthermore, prioritizing investments in transparency, explainability, and the ongoing refinement of AI algorithms is paramount to achieving clinical utility. The dawn of realizing the full potential of medical AI is upon us, and this journey mandates an unwavering commitment to ethics and an unceasing quest for progress. The future of cancer care beckons, and it's our collective responsibility to answer that call.

## Supplementary Information

Additional file 1. Protocol. This document presents the protocol developed for the scoping review.

Additional file 2. PRISMA 2020 Checklist. This file contains the completed PRISMA 2020 checklist documenting the reporting of the scoping review methodology and findings.

Additional file 3. Search Strategy. This document details the complete search strategy and database-specific filters applied in the scoping review.

Additional file 4. Ranking Filter. This document contains the Python-based ranking filter developed to filter journals based on SCImago Journal Rank metrics.

Additional file 5. Data Charting. This spreadsheet presents the comprehensive data extraction and charting results from the articles selected for inclusion in the scoping review.

## Data Availability

The data required and generated by this study are provided as supplementary materials.

## Abbreviations

A-HGD: Adenoma with High-Grade Dysplasia
A-LGD: Adenoma with Low-Grade Dysplasia
ACC: Accuracy
ACT: Atypical Cartilaginous Tumor
AdaBoost: Adaptive Boosting
advMRI: Advanced MRI
AI: Artificial Intelligence
AJCC: American Joint Committee on Cancer
ANN: Artificial Neural Network
AUC: Area Under the ROC Curve
BIRCH: Balanced Iterative Reducing and Clustering
BLT: Benign Laryngeal Tumors
CADe: Computer-Aided Detection
CADx: Computer-Aided Diagnosis
CECT: Contrast-Enhanced CT
CDS: Clinical Decision Support
cMRI: Conventional MRI
CNN: Convolutional Neural Network
CNS: Central Nervous System
CS: Chondrosarcoma
CSPCa: Clinically Significant Prostate Cancer
CT: Computed Tomography
DBSCAN: Density-Based Spatial Clustering of Applications with Noise
DL: Deep Learning
DNN: Deep Neural Network
DSC: Dynamic Susceptibility Contrast
DT: Digital Twin
DWI: Diffusion-Weighted Imaging
EIN: Endometrial Intraepithelial Neoplasia
EHR: Electronic Health Record
EUS: Endoscopic Ultrasonography
ExtraTrees: Extremely Randomized Trees
FLAIR: Fluid-Attenuated Inversion Recovery
FPR: False Positive Rate
FRAX: Fracture Risk Assessment Tool
Grad-CAM: Gradient-weighted Class Activation Mapping
GB: Gradient Boosting
GBDT: Gradient Boosted Decision Tree
GE-DSC: General Electric-Dynamic Susceptibility Contrast
H&E: Hematoxylin and Eosin
HRT: How-Risk Thymoma
JAFROC: Jackknife Alternative Free-Response ROC
JBI: Joanna Briggs Institute
kNN: K-Nearest Neighbors
LCA: Laryngeal Cancer
LCSGJ: Liver Cancer Study Group of Japan
LightGBM: Light Gradient-Boosting Machine
LRT: Low-Risk Thymoma
MAE: Mean Average Error
MDA: Mixture Discriminant Analysis
MDT: Mean Diagnostic Time
mGPS: Modified Glasgow Prognostic Score
ML: Machine Learning
MLP: Multi-Layer Perceptron
mpMRI: Multiparametric MRI
MRI: Magnetic Resonance Imaging
MRS: Magnetic Resonance Spectroscopy
N-MTLR: Neural Multitask Logistic Regression
NB: Naïve Bayes
NCI: National Cancer Institute
NCRT: Neoadjuvant Chemotherapy
NECT: Non-Constrast CT
NHGRP: National Human Genetic Resources Sharing Service Platform
NLST: National Lung Screening Trial
NPV: Negative Predictive Value
NR: Not Reported
NSCLC: Non-Small Cell Lung Cancer
OSTA: Osteoporosis Self-Assessment Tool for Asians
PA: Pennsylvania
PCA: Principal Component Analysis
PCC: Population/Concept/Context
PET-CT: Positron Emission Tomography – Computed Tomography
PI-RADS: Prostate Imaging Reporting and Data System
PKU: Peking University
PLCO: Prostate, Lung, Colorectal, and Ovarian Cancer Screening Trial
PPV: Positive Predictive Value
PRELCA: Precancerous Laryngeal Lesions
PRISMA-ScR: Preferred Reporting Items for Systematic Reviews and Meta-Analysis extension for Scoping Reviews
qBOLD: Quantitative Blood-Oxygenation-Level-Dependent
QSVM: Quadratic SVM
RECIST: Response Evaluation Criteria in Solid Tumors
RF: Random Forest
RNN: Recurrent Neural Network
ROC: Receiver Operating Characteristic
RWD: Real World Data
SEN: Sensitivity
SEER: Surveillance, Epidemiology, and End Results
SHAP: SHapley Additive exPlanations
SJR: Scientific Journal Rankings
Sos: Softspot
SPEC: Specificity
SVC: Support Vector Classifier
SVM: Support Vector Machine
Sws: Sweetspot
TC: Thymic Carcinoma
TCGA: The Cancer Genome Atlas
TCIA: The Cance Imaging Archive
TET: Thymic Epithelial Tumor
TNM: Tumor, Node, and Metastasis staging system
TNM-7: Seventh edition of the TNM staging system
TNM-8: Eighth edition of the TNM staging system
TRG: Tumor Regression Grade
UK: United Kingdom
USA: United States of America
VA: Veterans Affairs
VAM: Vascular Architecture Mapping
WHO: World Health Organization
WSI: Whole-Slide Image
XAI: Explainable AI
XGBoost: Extreme Gradient Boosting
Y: Years

## Footnotes

1 The number of patients was not reported in two documents [62, 63].

2 The number of women and men was not described in 8 articles [71, 99, 73, 109, 100, 62, 63, 96].

3 Not reported in [62, 63].

4 Not reported in [71, 99, 73, 100, 62, 63].

5 Not reported in [109].

6 Not reported in [96].
