# Supplementary material for: Externally validated and clinically useful machine learning algorithms to support patient-related decision-making in oncology: a scoping review
Source: BMC Med Res Methodol. 2025 Feb 21;25:45. doi: 10.1186/s12874-025-02463-y (PMC11843972; doi:10.1186/s12874-025-02463-y)
Supplement: Supplementary file 1 — Additional file 1. Protocol. This document presents the protocol developed for the scoping review. [file 12874_2025_2463_MOESM1_ESM.pdf]

EXTERNALLY VALIDATED AND CLINICALLY USEFUL MACHINE LEARNING ALGORITHMS TO SUPPORT PATIENT-RELATED DECISION-MAKING IN ONCOLOGY: A SCOPING REVIEW PROTOCOL

Authors

Catarina Sousa Santos[1], Mário Amorim-Lopes[1]
[1]INESC-TEC, Faculdade de Engenharia, Universidade do Porto

Abstract

[412/500 words]

Objective: This scoping review aims to systematically map externally validated machine learning-based models developed for patient care in oncology, quantify their performance and clinical utility, and associate specific models with particular types of cancer and decisions to be made, as well as reveal research gaps in these areas.

Introduction: Although numerous algorithms are developed for cancer-related decisions, the overwhelming majority has yet to reach oncology practice, mainly due to subpar methodological reporting and validation standards. When presenting a new or updated machine learning (ML) method, demonstrating its performance on the patients used for development (internal validation) is unsatisfactory, especially for small sample sizes, as this does not ensure clinical validity. However, although several systematic and scoping reviews concerning patient-related decision-making in oncology do exist, these are either context-specific, not approached from a machine-learning perspective, not focused on external (or clinical) validation, or focused on the lack of reporting standards and risk of bias. Furthermore, none of these reviews gather which machine learning algorithms were used, how performance was assessed, and if any correlations could be found between cancer types and specific ML methods.

Inclusion criteria: Quantitative primary research papers written in English and published in peer-reviewed journals will be considered. The primary concept is the use of externally validated and clinically useful machine learning algorithms to assist in clinical decision-making. The population consisted of human cancer patients (with no restriction for age groups, demographics, or cancer types). The records' main focus must be on clinical outcomes for cancer patients. Machine or deep learning models had to be externally validated with reporting performance metrics, and clinical utility must have been assessed. The context is any cancer-patient-related settings and assessments (e.g., diagnosis, outcome prediction, and surveillance) as long as data is commonly available in most clinical settings. Thus, genetic studies (e.g., omics, genetics, molecular biomarkers) will not be included.

Methods: The proposed scoping review will follow the Joanna Briggs Institute's (JBI) methodology for scoping reviews and the Preferred Reporting Items for Systematic reviews and Meta-Analyses extension for Scoping Reviews (PRISMA-ScR). The Population, Concept and Context (PCC) mnemonic will guide the review. It will cover quantitative scientific literature published in journals since January 1, 2014 whose Scimago Journal and Country Ranking is higher than one and whose best quartile is Q1. The following databases will be searched: Embase, IEEE Xplore, PubMED, Scopus, and Web of Science. Results will be summarized in diagrammatic, tabular and graphical form, accompanied by descriptive statistics and narrative synopses.

Objectives

The presented scoping review will systematically gather publications regarding externally validated machine (and deep) learning models developed **with commonly collected data** to provide clinical decision support to oncologists and other health professionals concerning cancer-related outcomes for individual patients. Our specific goals are to:

- Map these models' focus, contents, and implementation status;
- Quantify their predictive performance;
- Determine how their clinical utility was established;
- Associate specific models with particular types of cancer and decisions to be made;
- Unveil research gaps in this field.

Review questions

The outlined research questions and sub-questions were defined as follows:

- What externally validated machine learning algorithms have been developed to assist patient-related decision-making in oncology practice?
    - For what types of cancer variants and clinical outcomes were these models developed?
    - How were the validation studies designed?
    - Which populations and types of inputs were used?
    - Have these methods been tested on real-world data?
    - Have the models been implemented in clinical practice?
    - How was performance assessed during external validation?
- How was clinical utility established for these methods?
    - Which comparators and metrics have been used?
- Which machine learning algorithms show the best performance depending on the type of cancer, clinical modalities, and the decision(s) to be made?
    - What are the reported effects of these ML-based models on decision-making and outcomes?
- What are the research gaps in this field?

Keywords

Clinical decision support; Deep learning; Electronic Health Records; Machine learning; Prediction models

Eligibility criteria

Population

As we intend to explore machine learning methods for all patient-related decision-making in oncology, we will not define age groups, demographics, medical specialties, specific tumor sites, organs, or patient cohorts (e.g., children with glioma). Samples can consist of human patients or lesions (for image classification), provided that the focus is on cancer patient outcomes.

Concepts

The central concept is new or updated externally validated machine or deep learning algorithms using routinely collected data to assist decision-making regarding clinical outcomes for cancer patients. Specifically, the focus is on quantifying their performance during external validation and clinical utility. Since genetic information is not generally available in EHRs, studies concerning sequencing, omics, and molecular biomarker discovery will not be considered. Animal studies and papers using synthetic patients will be discarded.

Machine learning models are broadly defined as having the ability to learn from and identify patterns in the available data without being explicitly programmed. These can be supervised or unsupervised and regression or non-regression based. **Deep learning** refers to subsets of artificial neural networks with two or more hidden layers. All commonly known machine learning algorithms will be considered, including, for example, boosting, random forests, neural networks, and support vector machines. Nonetheless, since the line between traditional statistical and machine learning models can be tenuous for homonymous methods (e.g., logistic regression, naïve Bayes), these will only be included if explicitly described as an ML model. These algorithms can either be qualified as clinical decision support systems (CDSS), computer-aided diagnosis or detection (CADx or CADe), or provided as a standalone tool. If aimed at replicating cancer patients, **digital twin** approaches will also be included, as these align with clinical prediction models. Algorithms developed for anything other than patient care (such as medical education, structured data collection, text classification, cohort-specific assessments, or EHR dashboards) will be excluded. Articles whose primary focus is not oncology will also be disregarded.

External validation refers to evaluating the model's performance on a dataset not used for development [13]. **Model performance** should be reported concerning calibration (using a calibration plot, for example), discrimination (AUC or C-index), and classification (e.g., accuracy, sensitivity, and specificity). Articles not stating which algorithms were used, not presenting those quantitative metrics, or only internally validated will be excluded. Randomly splitting the patient dataset or cohort – whichever the sample size – into training and test sets

will be perceived as internal validation [13] and therefore excluded. Although not unanimously qualified as an external assessment, papers reporting model performance on temporally different datasets (temporal validation) will also be included.

Clinical validation involves testing the model on real-world data collected from routine care (e.g., EHRs) rather than synthetic patients or pre-existing datasets. However, models externally validated on pre-existing datasets will also be considered as long as they are composed of actual patients (not necessarily collected in routine care). Animal studies and synthetic patients will not be considered.

Clinical utility entails quantifying the impact of the model on decision-making and health outcomes through comparative assessments (e.g., clinicians performing the same task with and without assistance or health outcomes before and after implementation). We will report on how clinical utility was established, i.e., how the model influenced decision-making or compared against standard care (or other control groups). Non-comparative articles will not be included.

Context

The context is any cancer-patient-related setting, including primary, secondary, tertiary, and quaternary oncology care. Possible cancer-related assessments include surveillance, diagnosis, survival, staging, and anticipation of the response to specific treatments, medications, and surgeries, among many others.

Types of Sources

This scoping review will consider quantitative experimental, quasi-experimental, and observational study designs and any additional relevant quantitative and comparative research frameworks. Qualitative studies, conference abstracts, and other secondary research designs (such as reviews, editorials, letters, and book chapters) will not be considered due to not typically reporting individual (if any) performance metrics, thus impeding quantitative analyses. Grey literature will also not be included. To limit the scope of this review and allow its reproducibility, it will only encompass peer-reviewed journal articles with institutional or open full-text access. Furthermore, to ensure quality and reliable reporting, papers will only be assessed for eligibility if published in journals whose SJR (Scimago Journal and Country Rank, an indicator of scientific journal prestige) is higher than one and whose best quartile is Q1 [24].

Methods

The proposed scoping review will follow the updated Joanna Briggs Institute (JBI) methodology for scoping reviews [25,26]. It will also follow the Preferred Reporting Items for

Systematic Reviews and Meta-Analysis extension for Scoping Reviews (PRISMA-ScR) [27] checklist, adapted to encompass the PRISMA 2020 statement [28].

As recommended by JBI [25,26], the Population/Concept/Context (PCC) mnemonic guided the identification of the main concepts, research questions, and search strategy in this review. Accordingly, keywords were divided into three categories: **machine-learning-based decision-making** ("machine learning" OR "deep learning" OR "classification" OR "regression" OR "clinical decision support" OR "computer-aided diagnosis" OR "computer-aided detection" OR "digital twin(s)" OR "decision-making"), cancer ("cancer" OR "oncology" OR "tumor(s)" OR "neoplasm(s)"), and **evaluation** ("comparison" OR "performance" OR "valid*").

Search strategy

The search strategy will aim to locate primary research papers published in peer-reviewed journals. An initial limited search of PubMed was undertaken to identify articles on the topic. The text words in the titles, abstracts, and keywords of relevant articles were used to develop a complete search strategy for the EMBASE, IEEE Xplore, PubMed, Scopus, and Web of Science databases, with the search terms adapted to each database (see Appendix I). This study selected IEEE Xplore to address computing articles, PubMed and EMBASE to include biomedical literature, and Scopus and Web of Science to cover multidisciplinary reports. Due to being part of the first author's Ph.D., a one-year timeframe was outlined. Therefore, the reference list of all included sources of evidence will not be screened for additional studies unless related to the original article, and only publications written in English will be considered for inclusion. Studies published from January 1, 2014, will be included as this year aligns with when deep learning became mainstream [15].

Study selection

Following the search, all identified citations will be collated in RIS format and uploaded into EndNote 20.4.1 /2022 (Clarivate Analytics, PA, USA) and deduplicated (first electronically, followed by a manual sweep). A Python script will then be used to filter publications by SJR ranking. The remaining citations will be imported into a spreadsheet, and titles and abstracts will be screened for assessment against the inclusion criteria for the review. To generate the Excel file, the EndNote citations will be exported as a tab-delimited text file, pasted into Excel, and saved as a workbook. Full-text inspection of the potentially relevant sources will then be assessed against the inclusion criteria by both authors. Reasons for excluding sources of evidence at a full-text level that do not meet the inclusion criteria will be recorded and reported in the scoping review. Disagreements at each stage of the selection process will be resolved through discussion among the authors. The results of the search and study inclusion process will be reported in full in the final scoping review. These will be

presented in a Preferred Reporting Items for Systematic Reviews and Meta-analyses extension for scoping review (PRISMA-ScR) flow diagram [27] updated per the PRISMA 2020 statement [28].

Data Extraction (Data Charting)

Data will be extracted from papers included in the scoping review by the first author and confirmed by the second author using a data extraction form developed by the first author. These data will be stored in Excel spreadsheets and include specific details about the participants, concept, context, study methods, and critical findings relevant to the review questions. A draft extraction form is provided (see Appendix II). The draft data extraction form will be iteratively modified and revised as necessary while extracting data from each included evidence source. Modifications will be detailed in the scoping review. Any disagreements between the reviewers will be resolved through discussion.

Data Analysis and Presentation

The data are to be presented in tabular and graphical form. A narrative summary will accompany the tabulated and charted results and describe how the results relate to the review's objective and questions.

Acknowledgments

This review was undertaken as part of the first author's (CS.) Ph.D. No other relevant information.

Funding

Portuguese National Funds financed this work through the funding agency FCT - Fundação para a Ciência e a Tecnologia, within project LA/P/0063/2020.

Conflicts of interest

There are no conflicts of interest in this project.

References

1. Kourou, K., Exarchos, T. P., Exarchos, K. P., Karamouzis, M. V. & Fotiadis, D. I. Machine learning applications in cancer prognosis and prediction. *Computational and Structural Biotechnology Journal* vol. 13 8–17 (2015).
2. Walsh, S. *et al.* Decision Support Systems in Oncology. *JCO Clin. Cancer Informatics* 1–9 (2019).
3. Wang, C. *et al.* Deep Learning to Predict EGFR Mutation and PD-L1 Expression Status in Non-Small-Cell Lung Cancer on Computed Tomography Images. *J. Oncol.* **2021**, (2021).
4. Sarkiss, C. A. & Germano, I. M. Machine Learning in Neuro-Oncology: Can Data

Analysis From 5346 Patients Change Decision-Making Paradigms? *World Neurosurg.* **124**, 287–294 (2019).

5. Vallières, M. *et al.* Responsible radiomics research for faster clinical translation. *Journal of Nuclear Medicine* vol. 59 189–193 (2018).
6. Hernandez-Boussard, T. *et al.* Digital twins for predictive oncology will be a paradigm shift for precision cancer care. *Nat. Med. 2021 2712* **27**, 2065–2066 (2021).
7. Björnsson, B. *et al.* Digital twins to personalize medicine. *Genome Med.* **12**, 10–13 (2019).
8. Rasheed, A., San, O. & Kvamsdal, T. Digital twin: Values, challenges and enablers from a modeling perspective. *IEEE Access* **8**, 21980–22012 (2020).
9. Fagherazzi, G. Deep Digital Phenotyping and Digital Twins for Precision Health: Time to Dig Deeper. *J. Med. Internet Res.* **22**, e16770 (2020).
10. Hassani, H., Huang, X. & MacFeely, S. Impactful Digital Twin in the Healthcare Revolution. *Big Data Cogn. Comput.* **6**, 83 (2022).
11. Bruynseels, K., Santoni de Sio, F. & van den Hoven, J. Digital Twins in Health Care: Ethical Implications of an Emerging Engineering Paradigm. *Front. Genet.* **9**, 31 (2018).
12. Dhiman, P. *et al.* Methodological conduct of prognostic prediction models developed using machine learning in oncology: a systematic review. *BMC Med. Res. Methodol.* **22**, 101 (2022).
13. Ramspek, C. L., Jager, K. J., Dekker, F. W., Zoccali, C. & Van DIepen, M. External validation of prognostic models: What, why, how, when and where? *Clinical Kidney Journal* vol. 14 49–58 (2021).
14. Dhiman, P. *et al.* Reporting of prognostic clinical prediction models based on machine learning methods in oncology needs to be improved. *J. Clin. Epidemiol.* **138**, 60–72 (2021).
15. Nagendran, M. *et al.* Artificial intelligence versus clinicians: Systematic review of design, reporting standards, and claims of deep learning studies in medical imaging. *BMJ* **368**, (2020).
16. Riley, R. D. *et al.* Minimum sample size for external validation of a clinical prediction model with a binary outcome. *Stat. Med.* **40**, 4230–4251 (2021).
17. Dhiman, P. *et al.* Risk of bias of prognostic models developed using machine learning: a systematic review in oncology. *Diagnostic Progn. Res.* **6**, (2022).
18. Weissler, E. H. *et al.* The role of machine learning in clinical research: transforming the future of evidence generation. *Trials* vol. 22 1–15 (2021).
19. Moons, K. G. M. *et al.* Risk prediction models: II. External validation, model updating, and impact assessment. *Heart* vol. 98 691–698 (2012).
20. Lambin, P. *et al.* Radiomics: The bridge between medical imaging and personalized medicine. *Nature Reviews Clinical Oncology* vol. 14 749–762 (2017).
21. Karhade, A. V. *et al.* Development of Machine Learning Algorithms for Prediction of 5-Year Spinal Chordoma Survival. *World Neurosurg.* **119**, e842–e847 (2018).
22. Pawloski, P. A., Brooks, G. A., Nielsen, M. E. & Olson-Bullis, B. A. A systematic review of clinical decision support systems for clinical oncology practice. *JNCCN Journal of the National Comprehensive Cancer Network* vol. 17 331–338 (2019).
23. Esteva, A. *et al.* Dermatologist-level classification of skin cancer with deep neural networks. *Nature* **542**, 115–118 (2017).

24. Guerrero-Bote, V. P. & Moya-Anegón, F. A further step forward in measuring journals' scientific prestige: The SJR2 indicator. *J. Informetr.* **6**, 674–688 (2012).

25. Peters, M. *et al.* Chapter 11: Scoping Reviews. in *JBI Manual for Evidence Synthesis* (JBI, 2020). doi:10.46658/jbimes-20-12.

26. Peters, M. D. J. *et al.* Updated methodological guidance for the conduct of scoping reviews. *JBI Evid. Synth.* **18**, 2119–2126 (2020).

27. Tricco, A. C. *et al.* PRISMA Extension for Scoping Reviews (PRISMA-ScR): Checklist and Explanation. *Ann. Intern. Med.* **169**, 467–473 (2018).

28. Page, M. J. *et al.* The PRISMA 2020 statement: An updated guideline for reporting systematic reviews. *The BMJ* vol. 372 (2021).

Appendices

Appendix I: Search strategy

PUBMED

(((((cancer*[Title] OR oncolog*[Title] OR tumor*[Title] OR neoplas*[Title] OR malign*[Title])) AND (("digital twin*"[Title/Abstract] OR "machine learning"[Title/Abstract] OR "deep learning"[Title/Abstract] OR "artificial* intelligen*"[Title/Abstract] OR "predict* model*"[Title/Abstract]))) AND (("precision medicine"[Title/Abstract] OR "personalized medicine"[Title/Abstract] OR "computer aided diagnosis"[Title/Abstract] OR "computer aided detection"[Title/Abstract] OR "prognos*"[Title/Abstract] OR "decision making"[Title/Abstract] OR "decision support"[Title/Abstract] OR "classification"[Title/Abstract] OR "regression"[Title/Abstract]))) AND ((valid* OR performance OR compar*))) AND (((((((((((("english"[Language]) AND (ffrft[Filter])) AND ("journal article"[Publication Type])) AND ((2014/1/1:2022/09/30[pdat]))) AND (excludepreprints[Filter])) NOT (comment[Filter])) NOT (systematicreview[Filter])) NOT (review[Filter])) NOT (dataset[Filter])) NOT (englishabstract[Filter])) NOT (retractionofpublication[Filter])) NOT (retractedpublication[Filter])) NOT (introductoryjournalarticle[Filter])) NOT (booksdocs[Filter]))

Appendix II: Data extraction instrument

General data

Authors	Year	Journal	Title	Citation	Study Type	Randomized data?	Study Design	Institutional Design	Cancer Type (General)	Cancer Type (Specific)	Uses_EHR?

Methods Information

method_stated_in_abstract?	Focus (General)	Focus (Specific)	relevant_non_ml_methods	best_ML_method	method_details/architecture	_single_vs_ensemble	compared_with_other_methods?	deep_learning?	explainable_model?

uses_transfer_learning?	_supervision	task	type_of_implementation	Interface	system_classification	tool_name	url	processing_time	software

External validation

mentioned_in_abstract?	real_world_data?	routinely_available_data?	clinical_setting?	IMPLEMENTED_IN_PRACTICE?	international_validation?	independent_validation?

number_of_institutions_in_validation	data_availability	public_databases_in_validation	validation_type	data_source	population_details	population_age_group

population_size	female_count	male_count	sample_size	sample_type	auc	auc_95CI	accuracy	accuracy_95CI

sensitivity_recall	sensitivity_95CI	specificity_True_Neg_Rate	specificity_95CI	PPV_precision	PPV_precision_95CI	NPV	NPV_95CI	f1_score

brier_score	C_index	C_index_95CI	reports_threshold?	reports_calibration_graphically

Clinical utility

mentioned_in_abstract?	comparators	comparators_details	metrics	results

Other information

limitations_stated?	limitations_description	Notes	Summary
