# Supplementary material for: Externally validated and clinically useful machine learning algorithms to support patient-related decision-making in oncology: a scoping review
Source: BMC Med Res Methodol. 2025 Feb 21;25:45. doi: 10.1186/s12874-025-02463-y (PMC11843972; doi:10.1186/s12874-025-02463-y)
Supplement: Supplementary file 3 — Additional file 3. Search Strategy. This document details the complete search strategy and database-specific filters applied in the scoping review. [file 12874_2025_2463_MOESM3_ESM.pdf]

Externally Validated and Clinically Useful Machine Learning Algorithms to Support Patient-Related Decision-Making in Oncology: A Scoping Review

Search strategy

This document presents the complete search strategy and filters applied in the scoping review. As recommended by the Joanna Briggs Institute's (JBI) guidelines [1,2], the Population/Concept/Context (PCC) mnemonic served as the basis to develop the search approach, which was customized for the EMBASE, IEEE Xplore, PubMed, Scopus, and Web of Science databases.

EMBASE

english:la AND (cancer*:ti OR oncolog*:ti OR tumor*:ti OR neoplas*:ti OR malign*:ti) AND ('digital twin*':ti,ab,kw OR 'machine learning':ti,ab,kw OR 'deep learning':ti,ab,kw OR 'artificial* intelligen*':ti,ab,kw OR 'predict* model*':ti,ab,kw) AND ('precision medicine':ti,ab,kw OR 'personalized medicine':ti,ab,kw OR 'computer aided diagnosis':ti,ab,kw OR 'computer aided detection':ti,ab,kw OR' prognos*':ti,ab,kw OR 'decision making':ti,ab,kw OR 'decision support':ti,ab,kw OR 'classification':ti,ab,kw OR 'regression':ti,ab,kw) AND (validat* OR performance OR compar*) AND [2014-2022]/py AND [01-01-2014]/sd NOT [01-10-2022]/sd AND 'article'/it

IEEE XPLORE

Filters applied: open-access; journals; 2014-2022

("Document Title":cancer* OR "Document Title":oncolog* OR "Document Title":tumor* OR "Document Title":neoplas* OR "Document Title":malign*) AND ("All Metadata": "digital twin*" OR "All Metadata": "machine learning" or "deep learning" OR "All Metadata": "artificial* intelligen*" OR "All Metadata": "predict* model*") AND ("All Metadata": "precision medicine" OR "All Metadata": "personalized medicine" OR "All Metadata": "computer aided diagnosis" OR "All Metadata": "computer aided detection" OR "All Metadata": "prognos*" OR "All Metadata": "decision making" OR "All Metadata": "decision support" OR "All Metadata": "classification" OR "All Metadata": "regression") AND ("Full Text & Metadata":valid* OR "Full Text & Metadata":performance OR "Full Text & Metadata":compar*)

PUBMED

(((((cancer*[Title] OR oncolog*[Title] OR tumor*[Title] OR neoplas*[Title] OR malign*[Title])) AND (("digital twin*"[Title/Abstract] OR "machine learning"[Title/Abstract] OR "deep learning"[Title/Abstract] OR "artificial* intelligen*"[Title/Abstract] OR "predict* model*"[Title/Abstract]))) AND (("precision medicine"[Title/Abstract] OR "personalized medicine"[Title/Abstract] OR "computer aided diagnosis"[Title/Abstract] OR "computer aided detection"[Title/Abstract] OR "prognos*"[Title/Abstract] OR "decision making"[Title/Abstract] OR "decision support"[Title/Abstract] OR "classification"[Title/Abstract] OR "regression"[Title/Abstract])))

AND ((valid* OR performance OR compar*))) AND ((((((((((((("english"[Language]) AND (ffrft[Filter])) AND ("journal article"[Publication Type])) AND ((2014/1/1:2022/09/30[pdat]))) AND (excludepreprints[Filter])) NOT (comment[Filter])) NOT (systematicreview[Filter])) NOT (review[Filter])) NOT (dataset[Filter])) NOT (englishabstract[Filter])) NOT (retractionofpublication[Filter])) NOT (retractedpublication[Filter])) NOT (introductoryjournalarticle[Filter])) NOT (booksdocs[Filter]))

SCOPUS

(TITLE (cancer* OR oncolog* OR tumor* OR neoplas* OR malign*) AND TITLE-ABS-KEY ("digital twin*" OR "machine learning" OR "deep learning" OR "artificial* intelligen*" OR "predict* model*") AND TITLE-ABS-KEY ("precision medicine" OR "personalized medicine" OR "computer aided diagnosis" OR "computer aided detection" OR "prognos*" OR "decision making" OR "decision support" OR "classification" OR "regression") AND ALL (valid* OR performance OR compar*) AND LANGUAGE (english)) AND PUBYEAR > 2013 AND PUBYEAR < 2023 AND (LIMIT-TO (DOCTYPE , "ar")) AND (LIMIT-TO (LANGUAGE , "English")) AND (LIMIT-TO (OA , "all")) AND (LIMIT-TO (SRCTYPE , "j")) AND (LIMIT-TO (PUBSTAGE , "final"))

WEB OF SCIENCE

FILTERS: NOT Document Types: Review Article; NOT Document Types: Early Access. DATES: 2014-01-01: 2022-09-30; Document Types: Article; Languages: English.

((((((((LA=(English)) AND DT=(Article)) AND TI=(cancer* OR oncolog* OR tumor* OR neoplas* OR malign*)) AND TI=("digital twin*" OR "machine learning" or "deep learning" OR "artificial* intelligen*" OR "predict* model*")) OR AB=("digital twin*" OR "machine learning" or "deep learning" OR "artificial* intelligen*" OR "predict* model*")) AND AK=("digital twin*" OR "machine learning" or "deep learning" OR "artificial* intelligen*" OR "predict* model*")) AND TI=("precision medicine" OR "personalized medicine" OR "computer aided diagnosis" OR "computer aided detection" OR "prognos*" OR "decision making" OR "decision support" OR "classification" OR "regression")) AND AB=("precision medicine" OR "personalized medicine" OR "computer aided diagnosis" OR "computer aided detection" OR "prognos*" OR "decision making" OR "decision support" OR "classification" OR "regression")) AND AK=("precision medicine" OR "personalized medicine" OR "computer aided diagnosis" OR "computer aided detection" OR "prognos*" OR "decision making" OR "decision support" OR "classification" OR "regression")) AND ALL=(validat* OR performance OR compar*)

REFERENCES

1. Peters, M. *et al.* Chapter 11: Scoping Reviews. in *JBI Manual for Evidence Synthesis* (JBI, 2020). doi:10.46658/jbimes-20-12.
2. Peters, M. D. J. *et al.* Updated methodological guidance for the conduct of scoping reviews. *JBI Evid. Synth.* **18**, 2119–2126 (2020).
