# Supplementary material for: Externally validated and clinically useful machine learning algorithms to support patient-related decision-making in oncology: a scoping review
Source: BMC Med Res Methodol. 2025 Feb 21;25:45. doi: 10.1186/s12874-025-02463-y (PMC11843972; doi:10.1186/s12874-025-02463-y)
Supplement: Supplementary file 4 — Additional file 4. Ranking Filter. This document contains the Python-based ranking filter developed to filter journals based on SCImago Journal Rank metrics. [file 12874_2025_2463_MOESM4_ESM.pdf]

EXTERNALLY VALIDATED AND CLINICALLY USEFUL MACHINE LEARNING ALGORITHMS TO SUPPORT PATIENT-RELATED DECISION-MAKING IN ONCOLOGY: A SCOPING REVIEW

Filter deduplicated EndNote20 results by SJR Ranking

Legend: This jupyter notebook contains a Python script to filter publications by Scimago Journal & Country Rank (SJR) ranking. To generate the CSV file, the EndNote citations are exported as a tab-delimited text file, pasted into Excel, and saved as a workbook. The remaining citations are imported into a spreadsheet, and titles and abstracts screened for assessment against the inclusion criteria for the review.

In [ ]:
```
import pandas as pd
from IPython.display import Markdown as md
```

Load scimago rankings

In [ ]:
```
rankings = pd.read_csv('files/rankings/scimagojr2021_extraEntries.csv', sep =';')[['Title','SJR','SJR Best Quartile']]
ranking_titles = [i.lower() for i in rankings.Title.unique().tolist()]
```

Load deduplicated EndNote file

In [ ]:
```
sources = pd.read_excel('files/references/ML_deduplicated.xlsx')
```

Assign each journal to its respective SJR

In [ ]:
```
rankings = rankings.rename(columns={'Title':'Journal_Title'})
rankings['Journal_Title'] = rankings.Journal_Title.str.lower()
```

In [ ]:
```
ranked_results = pd.merge(sources, rankings, left_on=sources['Journal'].str.lower(),
                          right_on=rankings['Journal_Title'].str.lower(), how = 'left')
```

Filter data by ranking (remove nulls and rankings below 0.9)

In [ ]:
```
min_sjr = 1
```

Keep journals with a high impact factor (that don't have SJR)

In [ ]:
```
high_impact = ['cancer and metabolism', 'complex and intelligent systems',
               'hepatobiliary surgery and nutrition', 'journal of hepatocellular carcinoma',
               'nar cancer', 'nar genomics and bioinformatics', 'npj precision oncology',
               'world journal of gastrointestinal surgery']
```

Remove rows without journal name

In [ ]:
```
ranked_results = ranked_results[-pd.isnull(ranked_results.Journal)].drop(['key_0', 'Journal_Title'],
                                                                         axis = 1).reset_index(drop = True)
len(ranked_results)
```

For journals with a high impact that don't have SJR, assign the minimum SJR ranking allowed and Q1 quartile so that they don't get erased

In [ ]:
```
ranked_results['Journal1'] = ranked_results['Journal'].str.lower()
ranked_results.loc[ranked_results['Journal1'].str.fullmatch('|'.join(high_impact)), 'SJR'] = str(min_sjr)
ranked_results.loc[ranked_results['Journal1'].str.fullmatch('|'.join(high_impact)), 'SJR Best Quartile'] = 'Q1'

# check if fixed
ranked_results[ranked_results['Journal1'].str.fullmatch('|'.join(high_impact))][['Journal', 'SJR', 'SJR Best Quartile']]
```

Drop rows with null rankings

In [ ]:
```
ranked_results = ranked_results[-pd.isnull(ranked_results.SJR)].reset_index(drop = True)
len(ranked_results)
```

Remove rows whose ranking is below the allowed minimum

Convert SJR to float

In [ ]:
```
ranked_results['SJR'] = [float(i.replace(',','.')) for i in ranked_results.SJR.tolist()]
ranked_results['SJR'].dtype
```

Remove SJR <= min_sjr

In [ ]:
```
ranked_results = ranked_results[(ranked_results.SJR > min_sjr) & (ranked_results['SJR Best Quartile'] == 'Q1')]
len(ranked_results)
```

Save file

In [ ]:
```
ranked_results.to_csv('files/Results_after_RankingFilter.csv', sep = ';', index = False)
```
